# Spread of Gamma (P.1) Sub-Lineages Carrying Spike Mutations Close to the Furin Cleavage Site and Deletions in the N-Terminal Domain Drives Ongoing Transmission of SARS-CoV-2 in Amazonas, Brazil

**DOI:** 10.1128/spectrum.02366-21

**Published:** 2022-02-23

**Authors:** Felipe Gomes Naveca, Valdinete Nascimento, Victor Souza, André de Lima Corado, Fernanda Nascimento, George Silva, Matilde Contreras Mejía, Maria Júlia Brandão, Ágatha Costa, Débora Duarte, Karina Pessoa, Michele Jesus, Luciana Gonçalves, Cristiano Fernandes, Tirza Mattos, Ligia Abdalla, João Hugo Santos, Alex Martins, Fabiola Mendonça Chui, Fernando Fonseca Val, Gisely Cardoso de Melo, Mariana Simão Xavier, Vanderson de Souza Sampaio, Maria Paula Mourão, Marcus Vinícius Lacerda, Érika Lopes Rocha Batista, Alessandro Leonardo Álvares Magalhães, Nathânia Dábilla, Lucas Carlos Gomes Pereira, Fernando Vinhal, Fabio Miyajima, Fernando Braga Stehling Dias, Eduardo Ruback dos Santos, Danilo Coêlho, Matheus Ferraz, Roberto Lins, Gabriel Luz Wallau, Edson Delatorre, Tiago Gräf, Marilda Mendonça Siqueira, Paola Cristina Resende, Gonzalo Bello

**Affiliations:** a Laboratório de Ecologia de Doenças Transmissíveis na Amazônia, Instituto Leônidas e Maria Deane, Fiocruz, Manaus, Amazonas, Brazil; b Laboratório de Flavivírus, Instituto Oswaldo Cruz, Fiocruz, Rio de Janeiro, Rio de Janeiro, Brazil; c Fundação Centro de Controle de Oncologia do Estado do Amazonas, Manaus, Amazonas, Brazil; d Laboratório de Diversidade Microbiana da Amazônia com Importância para a Saúde, Instituto Leônidas e Maria Deane, Fiocruz, Manaus, Amazonas, Brazil; e Fundação de Vigilância em Saúde do Amazonas - Dra. Rosemary Costa Pinto, Manaus, Amazonas, Brazil; f Laboratório Central de Saúde Pública do Amazonas, Manaus, Amazonas, Brazil; g Universidade do Estado do Amazonas, Manaus, Amazonas, Brazil; h Hospital Adventista de Manaus, Manaus, Amazonas, Brazil; i Fundação de Medicina Tropical Doutor Heitor Vieira Dourado, Manaus, Amazonas, Brazil; j Instituto Nacional de Infectologia Evandro Chagas, Fiocruz, Rio de Janeiro, Rio de Janeiro, Brazil; k Laboratório de Diagnóstico e Controle e Doenças Infecciosas da Amazônia, Instituto Leônidas e Maria Deane, Fiocruz, Manaus, Amazonas, Brazil; l Secretaria de Saúde de Aparecida de Goiânia, Goiás, Brazil; m Laboratório de Virologia e Cultivo Celular, Instituto de Patologia Tropical e Saúde Pública, Universidade Federal de Goiás, Goiânia, Goiás, Brazil; n HLAGYN-Laboratório de Imunologia de Transplantes de Goiás, Aparecida de Goiânia, Goiás, Brazil; o Laboratório Analitico de Competências Moleculares e Epidemiológicas, Fundação Oswaldo Cruz Ceará, Fiocruz, Eusébio, Ceará, Brazil; p Unidade de Apoio Diagnóstico à COVID-19, Fundação Oswaldo Cruz Ceará, Fiocruz, Eusébio, Ceará, Brazil; q Departamento de Virologia, Instituto Aggeu Magalhães, Fiocruz, Recife, Pernambuco, Brazil; r Departamento de Entomologia e Núcleo de Bioinformática, Instituto Aggeu Magalhães, Fiocruz, Recife, Pernambuco, Brazil; s Departamento de Biologia, Centro de Ciências Exatas, Naturais e da Saúde, Universidade Federal do Espírito Santo, Alegre, Espírito Santo, Brazil; t Instituto Gonçalo Moniz, Fiocruz, Salvador, Bahia, Brazil; u Laboratório de Vírus Respiratórios e do Sarampo (LVRS), Instituto Oswaldo Cruz, Fiocruz, Rio de Janeiro, Rio de Janeiro, Brazil; v Laboratório de AIDS e Imunologia Molecular, Instituto Oswaldo Cruz, Fiocruz, Rio de Janeiro, Rio de Janeiro, Brazil; Wuhan Institute of Virology

**Keywords:** COVID-19, SARS-CoV-2, coronavirus, virus evolution, Brazil, variant gamma

## Abstract

The Amazonas was one of the most heavily affected Brazilian states by the COVID-19 epidemic. Despite a large number of infected people, particularly during the second wave associated with the spread of the Variant of Concern (VOC) Gamma (lineage P.1), SARS-CoV-2 continues to circulate in the Amazonas. To understand how SARS-CoV-2 persisted in a human population with a high immunity barrier, we generated 1,188 SARS-CoV-2 whole-genome sequences from individuals diagnosed in the Amazonas state from 1st January to 6th July 2021, of which 38 were vaccine breakthrough infections. Our study reveals a sharp increase in the relative prevalence of Gamma plus (P.1+) variants, designated Pango Lineages P.1.3 to P.1.6, harboring two types of additional Spike changes: deletions in the N-terminal (NTD) domain (particularly Δ144 or Δ141-144) associated with resistance to anti-NTD neutralizing antibodies or mutations at the S1/S2 junction (N679K or P681H) that probably enhance the binding affinity to the furin cleavage site, as suggested by our molecular dynamics simulations. As lineages P.1.4 (S:N679K) and P.1.6 (S:P681H) expanded (Re > 1) from March to July 2021, the lineage P.1 declined (Re < 1) and the median Ct value of SARS-CoV-2 positive cases in Amazonas significantly decreases. Still, we did not find an increased incidence of P.1+ variants among breakthrough cases of fully vaccinated patients (71%) in comparison to unvaccinated individuals (93%). This evidence supports that the ongoing endemic transmission of SARS-CoV-2 in the Amazonas is driven by the spread of new local Gamma/P.1 sublineages that are more transmissible, although not more efficient to evade vaccine-elicited immunity than the parental VOC. Finally, as SARS-CoV-2 continues to spread in human populations with a declining density of susceptible hosts, the risk of selecting more infectious variants or antibody evasion mutations is expected to increase.

**IMPORTANCE** The continuous evolution of SARS-CoV-2 is an expected phenomenon that will continue to happen due to the high number of cases worldwide. The present study analyzed how a Variant of Concern (VOC) could still circulate in a population hardly affected by two COVID-19 waves and with vaccination in progress. Our results showed that the answer behind that was a new generation of Gamma-like viruses, which emerged locally carrying mutations that made it more transmissible and more capable of spreading, partially evading prior immunity triggered by natural infections or vaccines. With thousands of new cases daily, the current pandemics scenario suggests that SARS-CoV-2 will continue to evolve and efforts to reduce the number of infected subjects, including global equitable access to COVID-19 vaccines, are mandatory. Thus, until the end of pandemics, the SARS-CoV-2 genomic surveillance will be an essential tool to better understand the drivers of the viral evolutionary process.

## INTRODUCTION

**FIG 1:**
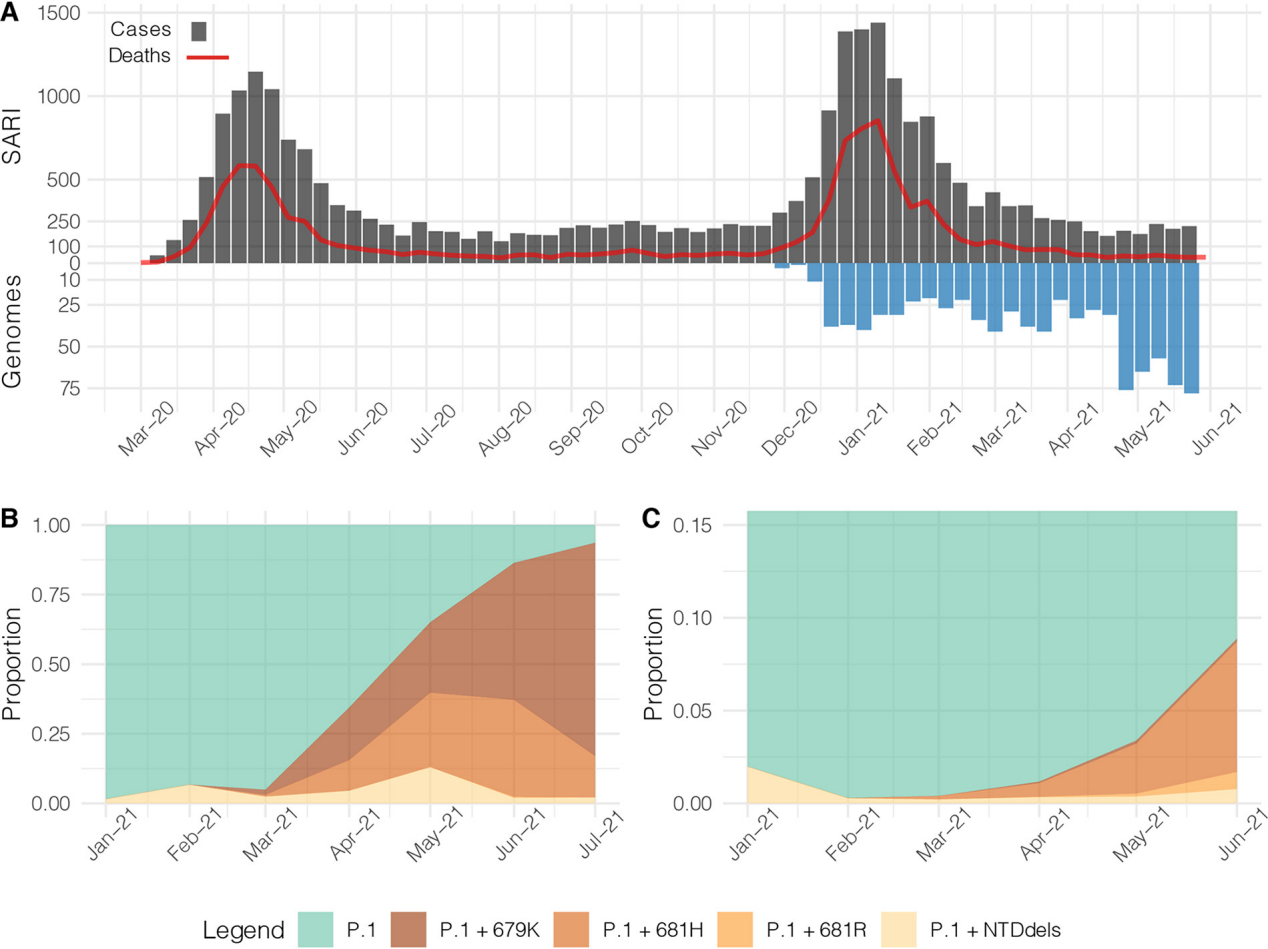
Temporal distribution and genetic diversity of SARS-CoV-2-positive samples from the Amazonas state during 2021. (A) Graph depicting the temporal evolution of SARI cases and SARI deaths based on the date of symptom onset (source, http://info.gripe.fiocruz.br) as a proxy for the COVID-19 epidemic curve in Amazonas state, along with the number of SARS-CoV-2 whole-genome sequences generated between January and July 2021. (B) Relative frequency of different P.1 lineage variants among SARS-CoV-2 positive cases sequenced in the Amazonas. (C) Relative frequency of different P.1 lineage variants among SARS-CoV-2 P.1 Brazilian sequences detected outside the Amazonas states.

The Amazonas was one of the most heavily affected Brazilian states by the COVID-19 epidemic, and by 31st July 2021, 13,531 deaths had been reported (1). The COVID-19 epidemic in Amazonas was characterized by two waves of exponential growth (Fig. 1A). The first one started in March 2020 and peaked around early May 2020 and was primarily associated with the introduction and dissemination of lineages B.1.195 and B.1.1.28 (2). The second one started in December 2020 and peaked around early February 2021 and was associated with the local emergence and rapid spread of the Gamma/P.1 lineage, a more transmissible SARS-CoV-2 Variant of Concern (VOC) first detected in Japanese travelers returning from the Amazonas State, Brazil (2–4). Since mid-February 2021, the number of SARS-CoV-2 deaths dropped and then remained roughly stable (7-day average <20) between May and July 2021.

Despite many infected people in the two waves of the COVID-19 pandemic and 32% of partial and 18% of fully vaccinated individuals in the Amazonas state on 31st July 2021 (5), the virus continues to circulate at a roughly steady-state level of ∼500 SARS-CoV-2 positive cases per day (7-day rolling average) from early May to mid-July 2021 (6). This endemic pattern of virus transmission supports the assumption that population immunity acquired in Amazonas, either through vaccinations or natural SARS-CoV-2 infection, is sufficient to prevent new exponential growth but not to stop the spread of the virus. We hypothesize that the endemic transmission of the VOC Gamma/P.1 in the Amazonas state allowed the rise of second-generation variants presenting a higher herd-immunity threshold than the parental virus.

To test this hypothesis, we combined phylodynamic approaches with epidemiological data to track mutations accumulated in SARS-CoV-2 whole-genomes recovered from individuals living in the Amazonas state diagnosed between January and July 2021. We demonstrate that persistent SARS-CoV-2 circulation in Amazonas was associated with a sharp increase in the relative prevalence of local P.1 plus (P.1+) variants harboring deletions in the N-terminal domain (NTD) or mutations at the S1/S2 junction of the Spike protein, while the parental P.1 lineage has been through a progressive extinction process since May 2021. New P.1+ lineages are more transmissible than the parental one, but natural and vaccine derived immunity are contributing to limit the spread of these lineages in the Amazonas state.

## RESULTS

### The ongoing evolution of lineage P.1 in the Amazonas state, Brazil.

To better understand the recent evolution of VOC gamma spreading in the Amazonas state, we sequenced the virus genome from 1,137 patients representing a random sample of SARS-CoV-2 positive cases diagnosed from 1st January to 6th July 2021. The Amazonas state health surveillance foundation sent SARS-CoV-2 positive samples from different municipalities for sequencing at FIOCRUZ Amazônia, part of the local health genomics network (REGESAM) and the consortium FIOCRUZ COVID-19 Genomics Surveillance Network of the Brazilian Ministry of Health (http://www.genomahcov.fiocruz.br/). Furthermore, we also sequenced the virus from 51 individuals living in Manaus who were part of a cohort of patients receiving the CoronaVac vaccine (CovacManaus https://www.ipccb.org/covacmanaus). This resulted in a total of 1,188 SARS-CoV-2 high-quality (<1% of undetermined “N” bases) whole-genome (>98% coverage) sequences, representing 0.6% of all laboratory-confirmed SARS-CoV-2 cases in the Amazonas state from 1st January to 6th July 2021 (*n *= 202,773) (Fig. 1A).

Our genomic survey confirms that VOC Gamma was the most prevalent lineage representing 99.7% (1,185 of 1,188 genomes) of all samples sequenced in the Amazonas state across the study period. We identified over 127 distinct amino acid (AA) substitutions and deletions in the S protein in addition to those that define lineage P.1, most of them present in less than 10 samples (Table S1). AA deletions covering in the NTD region (Δ144, Δ141-144 and Δ138-143) and substitutions at the S1/S2 junction (N679K, P681H and P681R), however, were particularly prevalent and sharply increase from January to May 2021 (Fig. 1B). The P.1+NTDdel variants increased from 1.6% in January to 12.4% in May 2021, while the P.1+N679K and P.1+P681H variants increased from 0% to 25.4% and 17.6%, respectively, in the same period. During June-July 2021, the proportion of P.1+N679K genomes continued to increase up to 76.9%, the relative frequency of P.1+P681H genomes increased up to 37.1% in June and then decrease to 13.5% in July, while the frequency of variants P.1+NTDdel dropped to 2.1%. Inspection of P.1 sequences available at EpiCoV database in the GISAID (https://www.gisaid.org/ 4) on July 22nd, 2021, also detected an increased frequency of variants P.1+NTDdel, P.1+N679K, P.1+P681H/R in other Brazilian states, but in a much lower prevalence than in the Amazonas (Fig. 1C).

### Identification of major P.1+ lineages in Amazonas and other Brazilian states.

**FIG 2:**
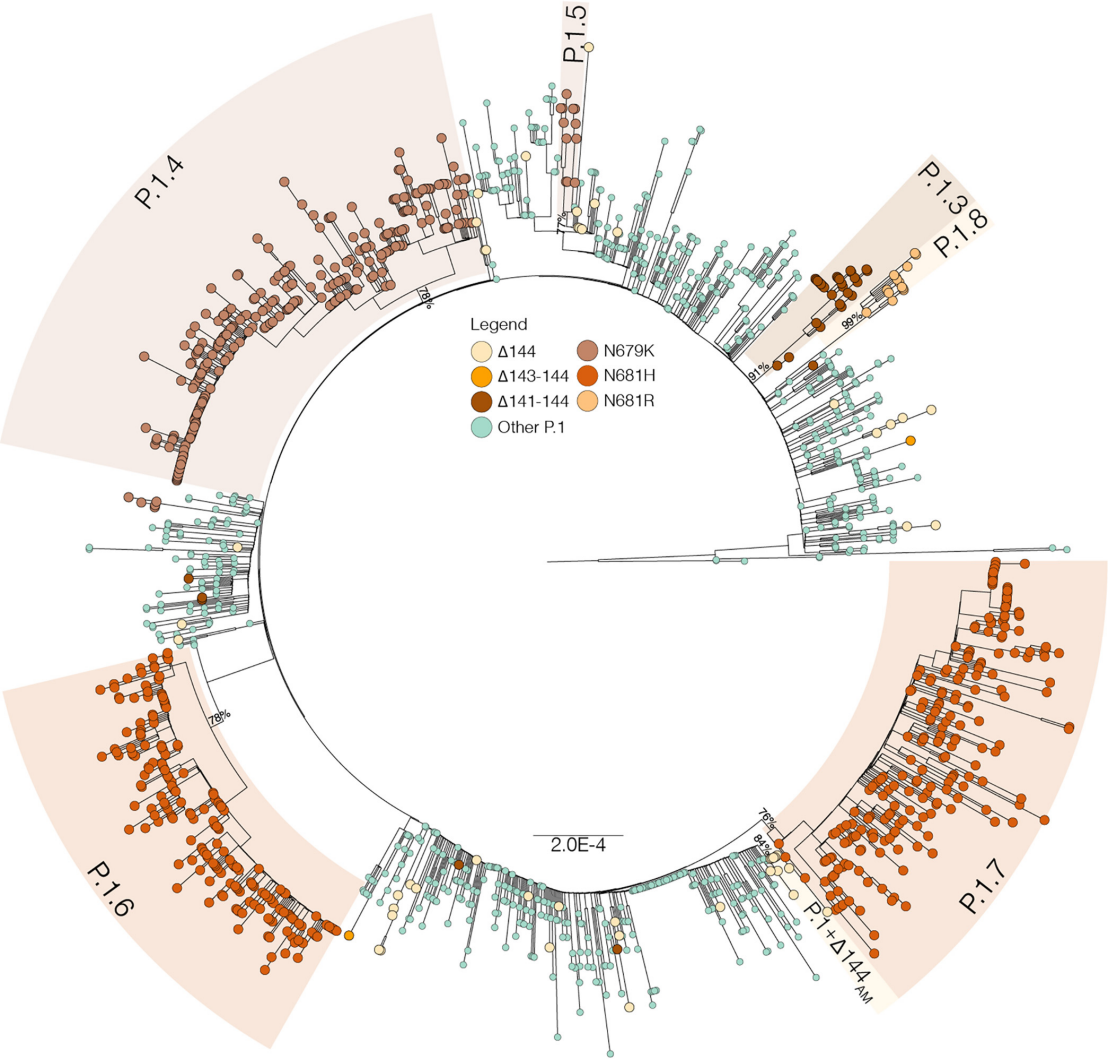
Maximum likelihood phylogenetic tree of P.1 Amazonian sequences and P.1+NTDdel, P.1+N679K, and P.1+P681H sequences detected outside Amazonas. Tips were colored according to the S mutations as indicated in the legend. Major P.1 sublineages carrying additional mutations/deletions in the S protein were highlighted with colored boxes. The aLRT support values are indicated in key branches, and branch lengths are drawn to scale with the bar indicating nucleotide substitutions per site.

To determine if the most frequent P.1 mutations detected in Brazil resulted from independent convergent mutations events, we combined the Amazonian P.1 sequences generated in this and previous studies with P.1+NTDdel (Δ144, Δ143-144, and Δ141-144), P.1+N679K and P.1+P681H/R sequences detected in other Brazilian states that were available at the EpiCoV database in GISAID (https://www.gisaid.org/). The Maximum Likelihood (ML) phylogenetic analysis supports that NTD deletions around position Y144 arose multiple (*n* > 20) times during the evolution of lineage P.1 in Brazil, in agreement with our previous observations (7), as well as mutations S:N679K (*n* > 5), S:P681H (*n* > 4) and S:P681R (*n* > 3) (Fig. 2). This analysis further revealed four well-supported (aLRT = 76–99%) monophyletic P.1+ sub-clades in Amazonas; some of which received the Pango Lineage designation P.1.3 to P.1.6 (Fig. 2). Lineage P.1.3 comprises most P.1+Δ141-144 sequences from the Amazonas state (*n* = 29/33, 88%). Lineage P.1.4 comprises most P.1+N679K sequences from the Amazonas state (*n* = 187/197, 95%) and three P.1+N679K sequences from Rio de Janeiro. Lineage P.1.5 comprises the remaining P.1+N679K sequences from the Amazonas state (*n* = 10/197, 5%) and two P.1+N679K sequences from Roraima and São Paulo states. Lineage P.1.6 comprises most P.1+P681H sequences from the Amazonas state (*n* = 208/209, 99%) and one P.1+P681H sequence from Rio de Janeiro. The fourth lineage, designated P.1+Δ144_AM_, comprises about half of P.1+Δ144 sequences from Amazonas (*n* = 12/29, 41%). Our analyses also revealed two major well-supported (aLRT = 76–99%) P.1+ lineages designated P.1.7 and P.1.8 that comprises most P.1+P681H (*n* = 227/234, 97%) and P.1+P681R (*n* = 13/20, 65%) sequences detected outside the Amazonas state, respectively. Analysis of the mutational profile reveals a variable number of lineage-defining mutations ranging from one in P.1.4 to nine in P.1.8 (Table 1 and Fig. S1). Interestingly, lineages P.1.4 and P.1.5 displayed the same lineage-defining AA substitution (N679K) but different nucleotide mutations (T23599G and T23599A). Most P.1+ sub-clades displayed only one AA lineage-defining mutation in the S protein, except P.1.8 that displayed three mutations (T470N, P681R, and C1235F).

**TABLE 1:** P.1+ lineage-defining mutations present in >95% of sequences

P.1 Sub-lineage	First detected	Nucleotide	Amino acid
P.1+Δ144_AM_	28th Apr 2021	C12513T Δ21992-21994	ORF1a:T4083M S:Δ141-144
P.1.3	12th Mar 2021	C5526T C16193T A21979T Δ21983-21994 T27826C C27942T	ORF1a:T1754I ORF1b:P909L -^*a*^ S:Δ141-144 ORF7b:M24T ORF8:H17Y
P.1.4	19th Mar 2021	T23599G	S:N679K
P.1.5	3rd Apr 2021	C3117T A18945G T23599A	ORF1a:T951I - S:N679K
P.1.6	26th Mar 2021	C10615T C15714T C23604A	- - S:P681H
P.1.7	12th Mar 2021	C1912T C16293T C23604A	- - S:P681H
P.1.8	24th May 2021	T592C A4040G C9891T A20055G C22971A C23604G G25266T C26039T G27915T	- ORF1a:I1259V ORF1a:A3209V - S:T470N S:P681R S:C1235F ORF3a:S261L ORF8:G8stop

a -, Represent silent mutations.

### Onset date and geographic spread of major Brazilian P.1+ lineages.

**FIG 3:**
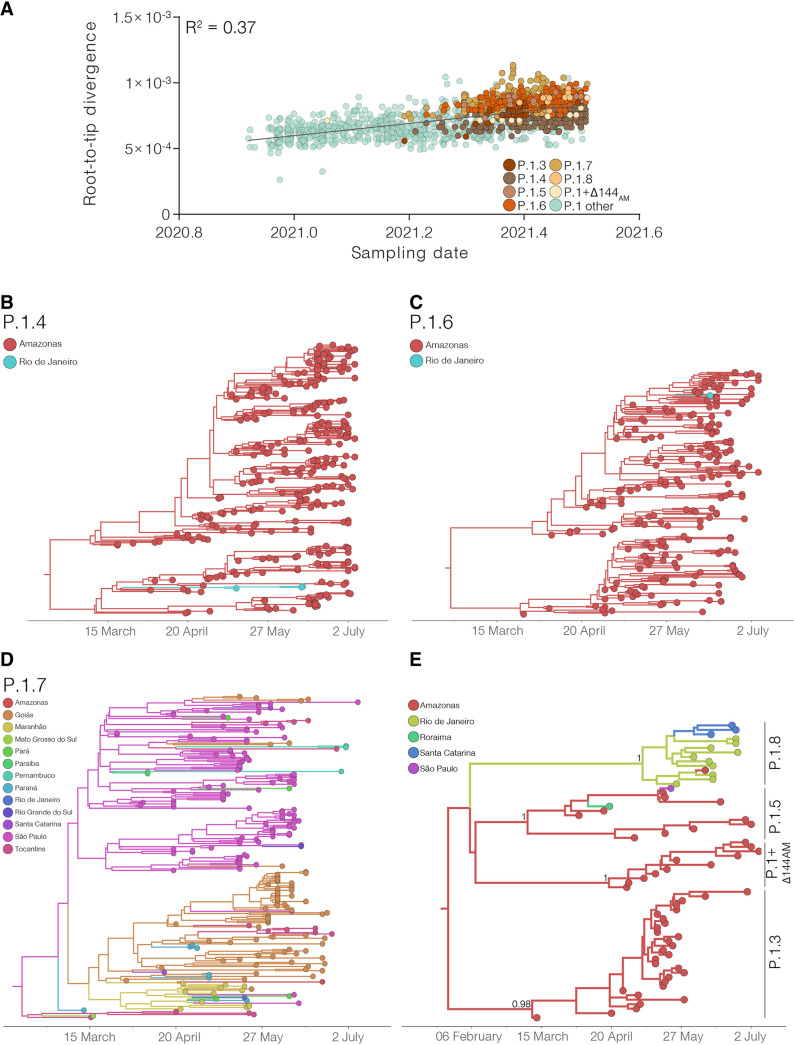
Temporal structure and phylogeographic reconstruction of the P.1+NTDdel, P.1+N679K, and P.1+P681H clades. (A) Root-to-tip regression of genetic divergence against dates of sample collection. P.1 sequences were colored green, while each P.1 subclade carrying deletions or additional mutations in S protein was colored following the legend. Time-resolved maximum clade credibility phylogenies of each P.1 subclade defined in the ML analysis: (B) P.1.4; (C) P.1.6; (D) P.1.7; (E) minor clades P.1.3, P.1.5, P.1.8 and P.1+Δ141-144AM. Tips and branches colors indicate the sampling state and the most probable inferred state of the nodes, respectively, as indicated in the legend for each tree. Bayesian posterior probabilities are indicated in key branches. All horizontal branch lengths are time-scaled, and the tree was automatically rooted under the assumption of the molecular clock model.

The analysis of temporal structure revealed that the overall divergence of the new P.1+ lineages is comparable with the divergence of other contemporaneous P.1 genomes circulating in the Amazonas, thus supporting a homogenous evolutionary rate among lineages (Fig. 3A). Bayesian time-scaled reconstruction estimated the emergence of the lineages P.1.3 at 7th Mar 2021 (95% HPD: 21st Feb – 12th Mar 2021), P.1.4 at 13th Feb 2021 (95% HPD: 9th Jan – 10th Mar 2021), P.1.5 at 15th Mar 2021 (95% HPD: 21st Feb – 30th Mar 2021), P.1.6 at 3rd March 2021 (95% HPD: 3rd Feb – 24th Mar 2021), P.1.7 on 16th Feb 2021 (95% HPD: 19th Jan – 5th Mar 2021), P.1.8 at 11th May (95% HPD: 1st May – 19th May 2021), and P.1+Δ144_AM_ at 21st Apr 2021 (95% HPD: 6th Apr – 28th Apr 2021) (Fig. 3B to E). Of note, the estimated median tMRCA of most P.1+ lineages circulating in Amazonas (13th Feb - 15th Mar) coincided with the declining phase of the second COVID-19 epidemic wave in the state.

As expected, phylogeographic reconstructions traced the Amazonas state as the most probable source location (PSP = 1) of lineages P.1.3, P.1.4, P.1.5, P.1.6, and P.1 +Δ144_AM_. These P.1 sublineages remained mostly restricted to the Amazonas state, except for a few sporadic disseminations to the Rio de Janeiro (lineages P.1.4 and P.1.6), Roraima (lineage P.1.5) and São Paulo (lineage P.1.5) states (Fig. 3B, C, and E). The state of São Paulo was pointed as the most probable (PSP = 0.68) epicenter of lineage P.1.7 dissemination to several states from Southern (Paraná, Rio Grande do Sul and Santa Catarina), Southeastern (Rio de Janeiro), Central-Western (Goiás and Mato Grosso do Sul), Northeastern (Alagoas, Maranhão, Paraíba, and Pernambuco), and Northern (Pará and Tocantins) Brazilian regions (Fig. 3D). We found evidence of large local transmission clusters of lineage P.1.7 in Goiás and Maranhão and of secondary disseminations from Goiás to Maranhão, Tocantins, Pará, Paraná and Santa Catarina, and from Maranhão to Amazonas, Pará, Paraná, Goiás and Rio de Janeiro. The state of Rio de Janeiro was pointed as the most probable epicenter (*PSP *= 1) of lineage P.1.8. Despite this variant's overall low estimated prevalence, we found evidence of its dissemination to the Amazonas and Santa Catarina states and of subsequent local transmission in Santa Catarina (Fig. 3E).

### Epidemic expansion of lineages P.1.4 and P.1.6.

**FIG 4:**
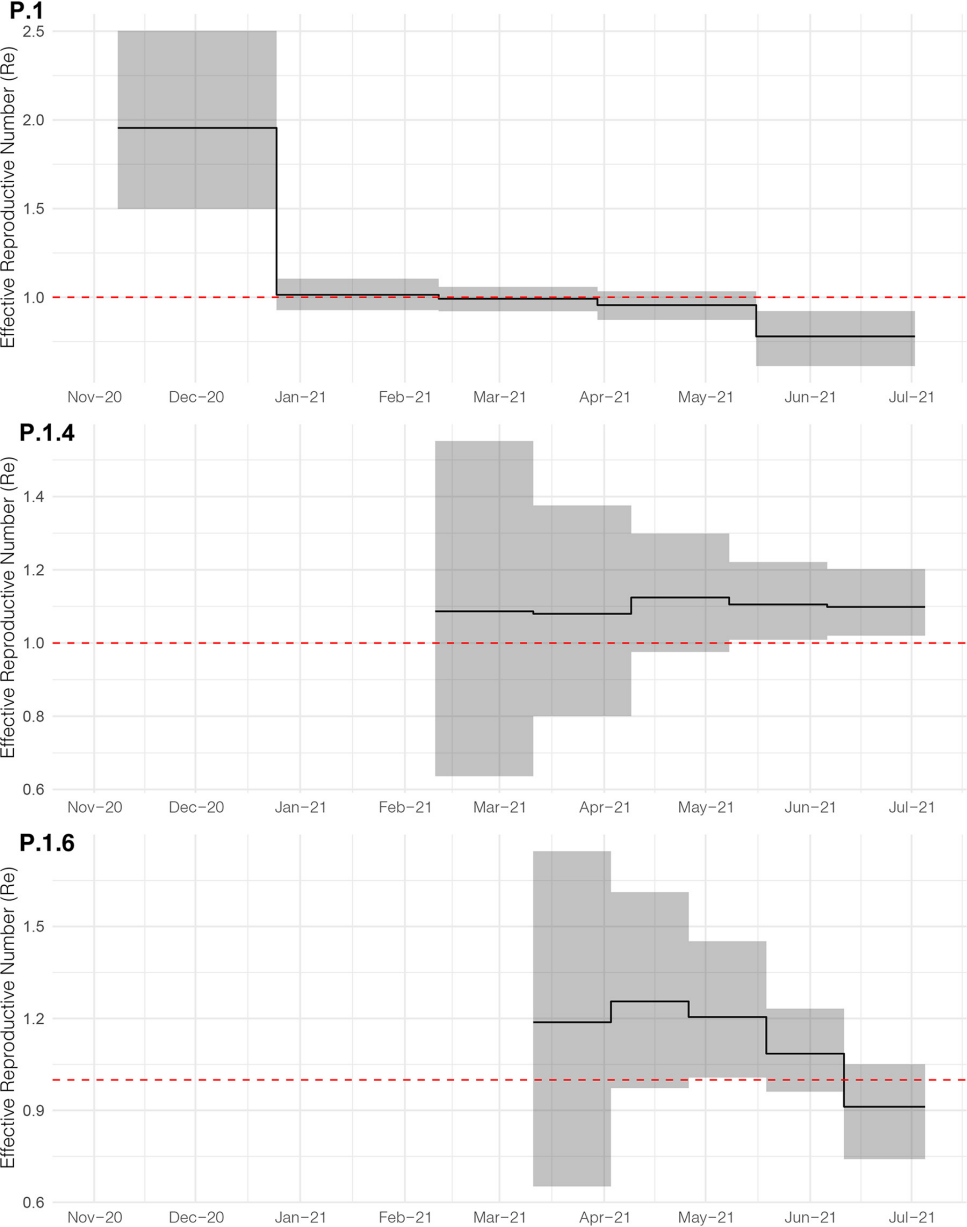
Epidemic trajectories of major SARS-CoV-2 lineages circulating in Amazonas in 2021. Graphs depicting the temporal variation in Re (median and 95% HPD) of Amazonian lineages P.1, P.1.4 and P.1.6 were estimated using the BDSKY approach.

The increasing relative frequency of lineages P.1.4 and P.1.6 suggest that those variants displayed an effective reproduction number (Re) >1 in the Amazonas state between March and July 2021. To confirm this hypothesis, we used the birth-death skyline (BDSKY) model to estimate the Re of lineages P.1, P.1.4, and P.1.6. Although the high posterior density (HPD) intervals were relatively large, the trajectory of the median Re values was consistent with each lineage's relative prevalence changes (Fig. 4). Lineage P.1.4 displayed a roughly constant median Re > 1 (1.08–1.12) overall the study period; while lineage P.1.6 displayed a median Re > 1 (1.09–1.25) from February to early June, but decreased to 0.91 in June-July, coinciding with the drop of its relative frequency from 37.1% in June to 13.5% in early July. Lineage P.1 displayed a median Re > 1 (1.96) during November-December 2020, a Re ∼1 during January-March 2021, and a Re < 1 (0.77 to 0.95) from April to July 2021. In June-July 2021, the ratio Re(P.1.4)/Re(P.1) was 1.41–1.42 and the ratio Re(P.1.6)/Re(P.1) was 1.17–1.38.

### The spread of P.1+ variants was associated with increasing community viral load.

**FIG 5:**
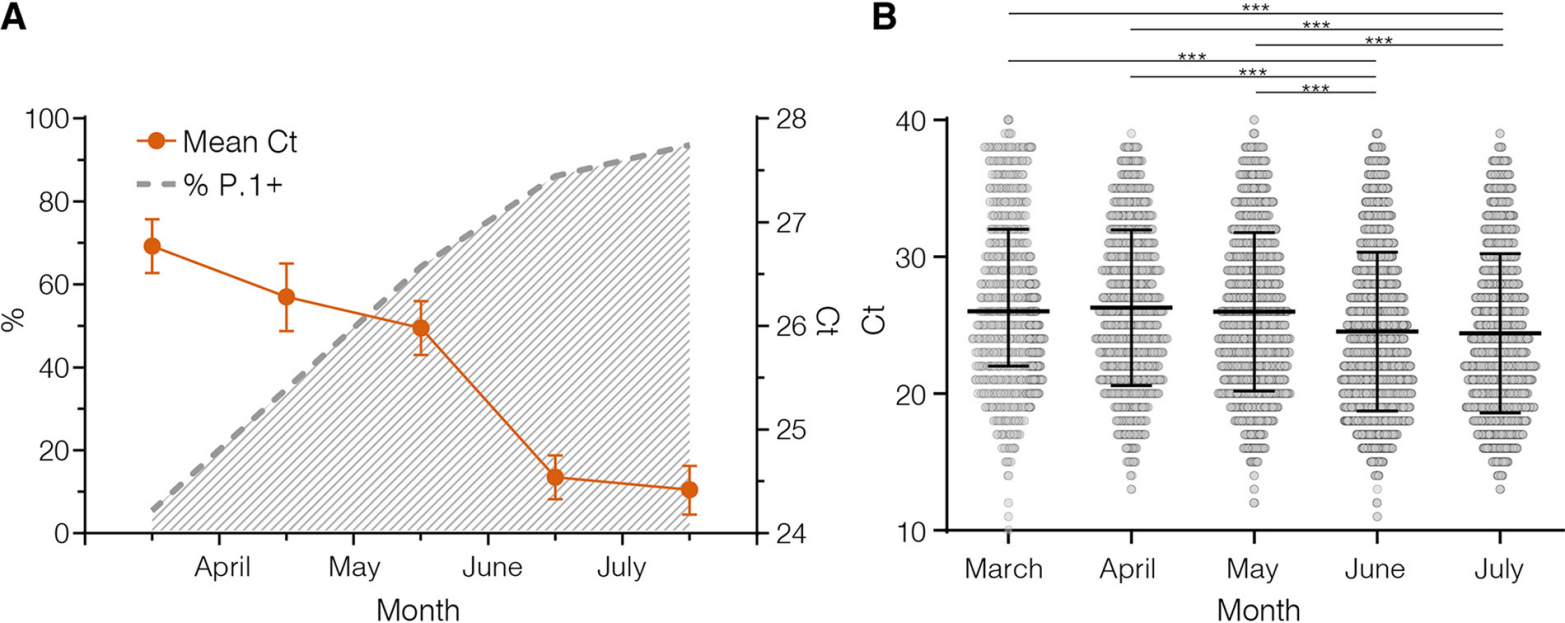
Estimation by RT–PCR of viral load in the upper respiratory tract of SARS-CoV-2 infected patients in Amazonas. (A) Graph depicting the relative prevalence of P.1+ lineages estimated from whole-genome sequencing (dashed gray line) and the Ct (mean and 95% Confidence Interval) among SARS-CoV-2 positive cases (solid line) in Amazonas between March and July 2021. (B) Comparison of Ct values from March to July 2021. Horizontal bars represent Ct medians and IQR. Two-sided *P* values for the nonparametric Mann–Whitney test are shown for each group. Two-sided *P*-values <0.05 were considered statistically significant.

The distribution of real-time RT–PCR cycle threshold (Ct) scores from single or successive cross-sectional samples is a consistent proxy for virus load in the underlying population and may be shaped by changes in both the infecting virus variant and the epidemic trajectory (8). To test if individuals infected with P.1+ variants might have a higher virus load in the upper respiratory tract than those infected with P.1, we compared the Ct values among SARS-CoV-2 cases diagnosed in Amazonas during the endemic phase of transmission (March to July 2021) when the number of SARS-CoV-2 cases remained roughly stable. Our analyses revealed that as the relative prevalence of P.1+ lineages increase, the mean Ct value of SARS-CoV-2 positive cases progressively reduced from 26.8 (95% CI:26.5–27.0) in March to 24.4 (95% CI:24.2–24.7) in July, which correspond to an ∼5-fold increase in the mean viral load of infected subjects (Fig. 5A). Median Ct values in June-July 2021 were significantly (*P* < 0.0001) lower than in March-May 2021 (Fig. 5B).

### The potential impact of S1/S2 mutations on Spike cleavability.

**FIG 6:**
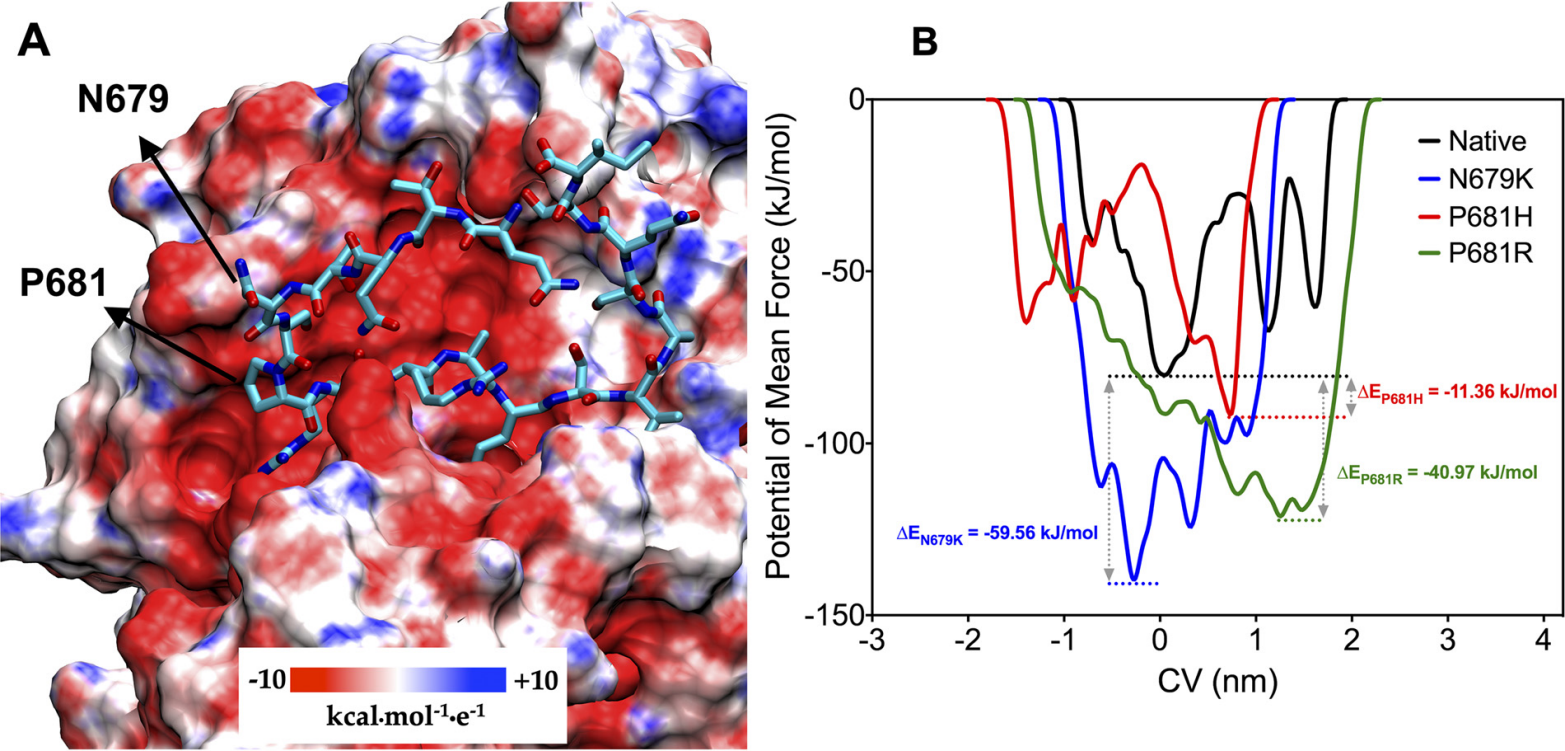
Binding between furin enzyme and the structural motif 679NSPRRARS686 of the SARS-CoV-2 Spike protein. (A) Representation of furin as electrostatic potential surface, showing the negative charge distribution around the loop substrate (represented in licorice cyan). Arrows indicate the native position of variant mutations N679K and P681H/R. (B) Free energy surface landscape of dissociation of modeled loops from furin enzyme depicted through the potential of mean force (PMF) as a function of the chosen CV. Dashed lines represent the lowest energy basin for the dissociation of the peptides.

Furin is an endoproteinase whose substrate is a polybasic structural motif of the type R-X-K/R-R↓ (where ↓ represents the peptide bond to be cleaved) (9, 10). In the S protein of SARS-CoV-2, furin recognizes the motif 681-PRRAR↓S-686 which is found in a long loop region between residues 675–692. The S1/S2 furin cleavage site (681PRRAR|S686) is of great importance for SARS-CoV-2 Spike protein structural changes during the initial steps of host cell infection and influence the invasion success and transmission of the virus (11, 12). The specificity of furin for a polybasic substrate is partly due to the high negative charge distributed close to the active site (Fig. 6A) and mutations S:P681H/R, that exchange neutral/nonpolar residues with basic amino acids close to the cleavage site, enhance the S1/S2 cleavability (13–17). We hypothesize that mutation S:N679K may also benefit the enzyme-substrate coupling.

To test this hypothesis, metadynamics simulations that modeled the dissociation of the substrate-enzyme complex were used to estimate the relative binding affinity between the wild-type (WT)/mutant peptides mimicking the S protein loop and the furin enzyme. The dissociation was freely conducted along the CVs-defined pathway, providing the potential of mean force (PMF) barrier between the WT complex and the pre-complex states. For metadynamics simulations in which the employed CV is protein – peptide distance, the PMF is equivalent to the free energy of binding (Δ*G*). The free energy landscape (FEL) was described through the PMF, depicting the phase space sampled from the CVs. It is worth noting that these values may differ quantitatively if the full S protein head was used. Additionally, the calculated values refer to energies obtained within the determined Gaussian parameters to the set of compounds under study. Thus, these energies should be used as a measure to compare the binding free energies among them, and not as absolute free energies. As it can be seen, the three PMFs display a well-defined energy minimum and metastable states during the dissociation process (Fig. 6B). From the PMF it is inferred that all mutants present more favorable binding energy, and therefore, higher affinity, compared to the WT peptide. Our analyses predict that replacement of a nonpolar residue (P) by a polar one (H) provides a modest increase in affinity compared to the WT peptide, whereas having a positively charged residue at either position 679 (K) or 681 (R) leads to a dramatic enhancement in binding affinity to the furin binding site (Fig. 6B).

### Frequency of P.1+ lineages among vaccine breakthrough cases.

**FIG 7:**
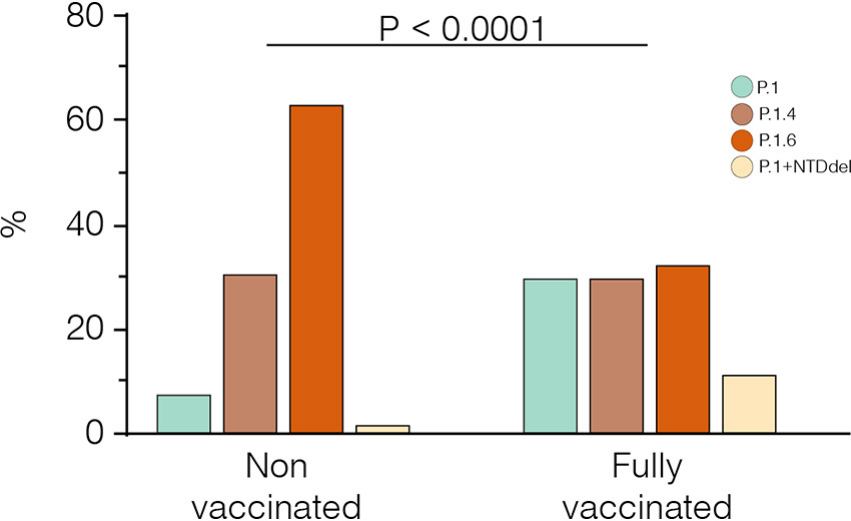
Relative prevalence of P.1 and P.1+ lineages among unvaccinated and fully vaccinated groups. All breakthrough cases were detected among individuals fully vaccinated with CoronaVac. The *P*-value for the Chi–Square test is shown. *P*-values <0.05 were considered statistically significant.

The expansion of P.1+ lineages in the Amazonas state may be partly associated with their higher ability to infect individuals who acquired immunity through vaccinations. To test this hypothesis, we compared the frequency of distinct P.1 variants in 38 fully vaccinated breakthrough cases (documented infection occurring 14 days after the second dose) and 104 unvaccinated individuals diagnosed in the Amazonas between April and July 2021. Our analyses revealed that P.1+ lineages were not overrepresented among breakthrough SARS-CoV-2 infections (71%) compared with unvaccinated controls (93%) infected in the same period (Fig. 7). Significant difference (*P* < 0.0001) in the overall distribution of P.1+ lineages among groups was mainly explained by the much higher frequency of lineage P.1.6 in the unvaccinated (63%) than in the fully vaccinated (32%) group. Although this may indicate a higher susceptibility of unvaccinated individuals to infection with lineage P.1.6 with respect to the fully vaccinated ones, this is a limited sample size and observations should be interpreted with caution.

## DISCUSSION

Understanding how SARS-CoV-2 persists in settings where a high proportion of the population have developed immunity against SARS-CoV-2, due to natural infection or vaccination, has important public health implications in the management of SARS-CoV-2 infections. In this study, we demonstrate that the endemic transmission of SARS-CoV-2 after the second COVID-19 epidemic wave in Amazonas in 2021 was associated with the continuous evolution of the VOC gamma through the acquisition of NTD deletions (mainly Δ144 and Δ141-44) or, more frequently, of S1/S2 mutations (P681H and N679K) in the Spike protein. The stable number of SARS-CoV-2 cases registered in the Amazonas between May and July 2021 resulted from two divergent underlying viral dynamics, the rise of the P.1+ variants and the concurrent extinction of the original lineage P.1.

Evidence supports that new P.1+ lineages emerging in Amazonas are more transmissible than the parental P.1 lineage. First, the main P.1+ lineages probably arose in the Amazonas state between mid-February and late April 2021, and their combined prevalence among SARS-CoV-2 positive cases that underwent genomic sequencing increased from 6% in March to >85% in June-July 2021. Second, while lineages P.1.4 and P.1.6 expanded (median Re >1) from March to July 2021, the parental lineage P.1 declined (median Re < 1) in the Amazonian population. Third, the increasing prevalence of P.1+ variants between March and June/July 2021, and particularly of lineages P.1.4 and P.1.6, was correlated with a ∼5-fold increase in the level of SARS-CoV-2 RNA estimated from the median Ct of positive samples in the Amazonas, suggesting that P.1+-infected adult individuals could be more infectious than those harboring the parental P.1 viruses.

Unfortunately, we have only detailed clinical data for 51 vaccine breakthrough patients who have undergone the COVACManaus study, an investigation to address if CoronaVac will protect subjects with comorbidities from severe illness. Among the 51 patients analyzed here, two infected with the parental P.1 lineage were hospitalized with moderate COVID-19 (one in the control group), with no need for respiratory support or oxygen therapy. Currently, all 51 patients were discharged without sequelae. Notwithstanding, it will be essential to determine if these P.1+ lineages are also associated with a distinct clinical evolution.

P.1+ lineages with convergent mutations/deletions also appeared independently in other Brazilian states. A previous study conducted by our group detected the concurrent emergence of multiple P.1+ lineages bearing NTD deletions (including Δ144 and Δ141-44) in several Brazilian states (7) and the present molecular survey identified the emergence of P.1 lineages with mutations S:N679K and S:P681H/R outside the Amazonas. The major P.1+ lineages circulating outside the Amazonas state were P.1.7 (P.1+P681H), which probably arose in the state of São Paulo around mid-February 2021 and is currently detected in at least 10 different states from all country regions; and P.1.8 (P.1+P681R), that probably arose in the state of Rio de Janeiro in early May 2021 and spread to the Southern (Santa Catarina) and Northern (Amazonas) regions. Although it is too early to determine whether these P.1+ lineages will become dominant outside the Amazonas state, their recurrent emergence suggests some transmissibility advantages over the P.1 original variant.

Most genetic changes identified in P.1+ lineages also appear in other VOCs and are predicted or known to affect virus infectivity, immune escape, or both. Different NTD deletions present in VOCs Alpha (Δ144), Beta (Δ241-243), Delta (Δ157-158) and Omicron (Δ143-145) confer resistance to neutralizing antibodies (NAb) directed against the NTD antigenic supersite; while the parental VOC Gamma that lack NTD deletions is more sensitive to those Nab (18–20). The two most common NTD deletions in P.1+ Amazonian lineages (Δ144 and Δ141-144) have been shown to emerge *in vivo* in long-term infections following therapy with convalescent plasma (21, 22) and during acute infections following production of autologous anti-NTD antibodies (23). Interestingly, NTD deletions Δ141 and Δ142 were among the selected forecasted mutations that may contribute to evolution of VOCs according to a recent study (24). Other S deletions outside the NTD antigenic supersite, like Δ69-70 that increases the viral infectivity (25), were not detected in our study suggesting that NTD deletions among the VOC Gamma evolving in the Amazonas state probably represent an adaptive mechanism of further immune evasion.

Several P.1 Brazilian sublineages (P.1.6, P.1.7 and P.1.8) have independently acquired mutations S:P681H/R at the multibasic furin motif that were also characteristic of other VOCs (Alpha, Delta, and Omicron) and VOIs (AV.1, B.1.1.318, B.1.617.1, B.1.617.3, and P.3). Both mutations have been shown to enhance the S1/S2 cleavability by furin-like proteases and mutation S:P681R, but not S:P681H, also enhances viral replication, viral fusion, and cell-cell viral spread *in vitro* when it occurs in the background of other S mutations (13–17). Furthermore, cleavage at S1/S2 junction is not only mediated by furin-like proteases, but also by cathepsins and other host proteases (26, 27), and mutations S:P681H/R may thus affect affinity to one or more different proteases and contribute to the high replication and transmissibility rate of different SARS-CoV-2 lineages.

The most prevalent Gamma variant in the Amazonas state by July 2021 was the lineage P.1.4 that harbors the mutation S:N679K, a genetic change that was recently observed in the VOC Omicron (28). Notably, the VOCs Gamma and Omicron also share mutation S:H655Y that was described to enhance S cleavage (29) and might thus function synergistically with mutations S:N679K and S:P681H/R. Our metadynamics simulations predicted that mutation S:N679K might lead to a dramatic enhancement in binding affinity to the furin binding site that was even higher than that predicted for mutations S:P681H/R, supporting that this mutation could contribute to the high replication and transmissibility of lineage P.1.4. Metadynamics simulations, however, should be interpreted with caution and need experimental validation. Nevertheless, recent experimental data demonstrate that when introduced individually into a plasmid expressing the wild-type Spike (D614G), mutation N679K undoubtedly increases S1/S2 cleavage (30). On the other hand, although the Omicron S has mutations that individually enhance furin cleavage (H655Y, N679K, and P681H), experimental evidence revealed that Omicron S is relatively poorly cleaved and exhibits reduced fusogenicity compared with other S variants (30–32). Altogether, these findings suggest that jointly these mutations may have an antagonistic effect on furin cleavage.

Two critical findings support that endemic transmission of lineage P.1 in the Amazonian population selected more infectious variants rather than variants that are more able to evade prior immunity triggered by natural infections or vaccines. First, we found no evidence that new P.1+ lineages circulating in the Amazonas were overrepresented among postvaccination breakthrough SARS-CoV-2 infections compared with infections among unvaccinated control. Second, the relative prevalence of P.1+ lineages harboring immune-escape NTD deletions increased to 12.4% in May but later decreased in June and July 2021, indicating that those variants displayed a transient transmission advantage despite the continuously growing number of immunized individuals in the Amazonas. This pattern is consistent with the hypothesis that the proportion of immune individuals in the Amazonas probably already exceeded the herd immunity threshold of most P.1 variants, except for those more transmissible lineages P.1.4 and P.1.6.

The evolutionary pattern of the VOC Gamma in Brazil has been different from the pattern that prompted the emergence of the immune evasive VOC Omicron in South Africa (28). The analysis of Gamma and Gamma-like sequences sampled in Brazil that were submitted to the GISAID until 9th December 2021 revealed that 31 out of the 37 Omicron S amino acid mutations have been individually observed across all Gamma diversity, including: all mutations at the NTD, 12 out of 15 mutations at the RBD, all mutations at the SD1/SD2, and three out of six mutations at the S2 subunit (Table S2). None of the Brazilian Gamma sequences observed, however, contained all those mutations combined. One of the most divergent Gamma sequences detected in Brazil was a P.1.4 lineage variant sampled in the Amazonas in October 2021 (EPI_ISL_5621224) that harbors 20 S mutations (eight more than the parental P.1 lineage), including several in common with (Δ144, Q498R, N501Y, H655Y, and N679K) or like (T95N, K417T, and E484K) those detected in Omicron. This suggests that the ecological conditions that fueled the huge number of S mutations detected in Omicron were probably not reproduced during evolution of Gamma in Brazil, despite the very high number of people naturally infected.

In summary, our study confirms that endemic transmission of SARS-CoV-2 after the second COVID-19 epidemic wave in the Amazonas state has been associated with the continuous evolution of the VOC gamma through the acquisition of either Spike mutations at the S1/S2 junction (N679K or P681H) or NTD deletions that probably increased viral infectivity or resistance against antiviral immunity. The steady-state level of SARS-CoV-2 cases observed in Amazonas between May and July 2021 resulted from the concurrent expansion of lineages P.1+ and extinction of parental lineage P.1. The spread of lineages P.1+ in the Amazonas state was probably limited by the increasing number of immunized (naturally infected and vaccinated) individuals. These findings highlight the importance of closely monitoring the evolution of SARS-CoV-2 VOCs as they continue to spread in human populations with decreasing density of susceptible hosts and the urgent need to accelerate the vaccination roll-out to reduce further viral transmission and evolution.

## MATERIALS AND METHODS

### SARS-CoV-2 samples and ethical aspects.

We analyzed nasopharyngeal and pharyngeal swabs (NPS) collected between 01st January and 06th July 2021 from residents in the Amazonas state positively tested by real-time RT-PCR as a routine diagnostic for SARS-CoV-2. In total, 1,188 samples were submitted to nucleotide sequencing at the Fiocruz/ILMD under the auspices of the Fiocruz COVID-19 Genomic Surveillance Network, the Amazonas State Health Foundation – Dra. Rosemary Costa Pinto (FVS-RCP), and the Brazilian Ministry of Health. This study was approved by the Ethics Committee of the Amazonas State University, which waived signed informed consent (CAAE:25430719.6.0000.5016).

### SARS-CoV-2 amplification and sequencing.

The SARS-CoV-2 genomes were recovered using a previously described sequencing protocol (33) or a commercial kit Illumina COVIDSeq Test (Illumina), with the following modifications in the original manufacturer instructions. The reverse transcription step was optimized to use half of the volume described in the Illumina COVIDSEQ Test protocol. We also included a longer incubation time (65°C for 5 min) and increasing sequential temperature steps 25°C 15’/37°C 15’/45°C 15’/50°C 15’/70°C 15’ and 4°C/∞. With these modifications we were able to sequence samples with Ct higher than 25, around 28–30. We also included custom primers at a final concentration of 20 nM to minimize the dropout observed in the primers target regions when only standard Illumina COVIDSeq Test primer set was used. (COVIDSEQ_3732_FNF 5′ - GTTGTTAATGCAGCCAATGTTTACCTTAAA, COVIDSEQ_4186_FNR CAACTTGCTTTTCACTCTTCATTTCCAAA, COVIDSEQ_20883_FNF 5′-TGCTAATTCCATTGTTTGTAGATTTGACACTA, COVIDSEQ_21285_FNR 5′ - CTGAAGTCTTGTAAAAGTGTTCCAGAGG, COVIDSEQ_24259_FNF 5′ - AACATCACTAGGTTTCAAACTTTACTTGCT, COVIDSEQ_24604_FNR 5′ - ATGCAAATCTGGTGGCGTTAAAAAC).

The new generation sequencing libraries were clustered with MiSeq reagent kit v3 (600-cycles) on 2 × 150 cycles paired-end runs (Illumina) or with NextSeq 1000 on 2 × 50 cycles. FASTQ reads were generated by the Illumina pipeline at BaseSpace (https://basespace.illumina.com). Consensus sequences were generated using DRAGEN COVID LINEAGE 3.5.1 to 3.5.3, according to the most up-to-date version of this app on each sequencing run. Subsequently, we evaluated the consensus files for quality using Nextclade tool v1.5.2 (https://clades.nextstrain.org), those with more than 1% of ambiguities “Ns” had the FASTQ files imported into Geneious Prime 2021 for trimming and assembling using a customized workflow employing BBDuk and BBMap tools (v38.84) and the NC_045512.2 RefSeq as a template with carefully visually inspection. Using both approaches, we generated consensus sequences with mean depth coverage higher than 800X, excluding duplicate reads. Whole-genome SARS-CoV-2 consensus sequences were initially assigned to viral lineages according to the nomenclature proposed by Rambaut et al. (34), using the Pango Lineage web application (https://pangolin.cog-uk.io) and later confirmed using phylogenetic analyses as explained below.

### Maximum likelihood phylogenetic analysis.

SARS-CoV-2 P.1 sequences obtained here were aligned with high quality (<1% of N) and complete (>29 kb) lineage P.1 Amazonian sequences that were available in the EpiCoV database in the GISAID (https://www.gisaid.org/) on May 31st, 2021. Sequences were aligned using MAFFT v7.475 (35) and then subjected to maximum-likelihood (ML) phylogenetic analysis using IQ-TREE v2.1.2 (36) under the general time-reversible (GTR) model of nucleotide substitution with a gamma-distributed rate variation among sites, four rate categories (G4), a proportion of invariable sites (I) and empirical base frequencies (F) nucleotide substitution model, as selected by the ModelFinder application (37). The branch support was assessed by the approximate likelihood-ratio test based on the Shimodaira–Hasegawa-like procedure (SH-aLRT) with 1,000 replicates. The temporal signal of the P.1 and P.1+ sequences was assessed from the ML tree by performing a regression analysis of the root-to-tip divergence against sampling time using TempEst (38).

### Bayesian phylogeography analysis.

We performed a time-scaled Bayesian phylogeographic analysis for SARS-CoV-2 genomes of lineages P.1.3 to P.1.8 plus P.1+Δ144 sampled in Brazil using the Bayesian Markov Chain Monte Carlo (MCMC) approach implemented in BEAST 1.10.4 (39) with BEAGLE library v3 (40) to improve computational time. The Bayesian tree was reconstructed using the GTR+F+I+G4 nucleotide substitution model, the nonparametric Bayesian skyline (BSKL) model as the coalescent tree prior (41), a strict molecular clock model with a uniform substitution rate prior (8 × 10^−4^ substitutions/site/year) and a reversible discrete phylogeographic model (42) with a continuous-time Markov chain (CTMC) rate reference prior (43). MCMC was run sufficiently long to ensure convergence (effective sample size [ESS] > 200) in all parameters estimates as assessed in TRACER v1.7 (44). The maximum clade credibility (MCC) tree was summarized with TreeAnnotator v1.10 and visualized using FigTree v1.4.4 (http://tree.bio.ed.ac.uk/software/figtree/).

### Effective reproductive number (re) estimation.

To estimate the Re trajectories of lineages P.1, P.1.4, and P.1.6 through time in the Amazonas, we used the birth-death skyline (BDSKY) model (45) implemented within BEAST 2 v2.6.5 (46). The sampling rate (d) was set to zero for the period before the oldest sample and estimated from the data afterward. The BDSKY prior settings were as follows: Become Uninfectious Rate (exponential, mean = 36); Reproductive Number (log-normal, mean = 0.8, sd = 0.5); Sampling Proportion (beta, alpha = 1, beta = 100). Origin parameter was conditioned to the root height, and the Re was estimated piecewise over five-time intervals. The molecular clock was as in the time-scaled trees analysis, and the HKY+G4+F substitution model was used. MCMC chains were run until all relevant parameters reached ESS > 200, as explained above.

### Modeling the relative binding strength of N679K, P681H and P681R variants to furin.

The furin cleavage site in the Spike protein of SARS-CoV-2 is found in a disordered region, exposed to the solvent. Due to the high flexibility of this loop, the experimentally resolved structures lack atomic coordinates in this region, even when mutations are introduced to express the protein in its pre-fusion conformation. Starting from the crystallographic structure of an inhibitor bound to human furin (PDB ID: 6HLB) (47), the 679-NSPRRARS-686 loop region was built analogously in the enzyme-interacting conformation (Fig. S2A). To mimic the complete loop linked to the Spike protein, the modeled motif was used as a folding seed (rotamers kept fixed) and the remaining loop residues were modeled using the Rosetta Remodel protocol (48) (Fig. S2B). The final model comprising the residues 675-QTQTNSPRRARSVASQSI-692 (Fig. S2C) was minimized using the FastRelax protocol of Rosetta (49) using the REF2015 energy function and maintaining the catalytic residues of furin fixed. Constraints to Ca2+ ion and its coordinating residues were applied with SetupMetalsMover (50). The variants N679K, P681H and P681R loops were built by using Point mutant (“pmut”) scan application of Rosetta onto a modeled native loop (51). The resultant mutant structures were then geometry-optimized following the same protocol with the FastRelax application. The relative binding free energies between furin and the peptides mimicking the Spike furin-loop variants were estimated by metadynamics simulations in an explicitly aqueous environment (52) (as detailed in the Supplemental Material).

### Statistical analysis.

Descriptive statistics, tests for normal distribution (D'Agostino & Pearson and Anderson-Darling), and the nonparametric Mann-Whitney test were used to compare the Ct of SARS-CoV-2 RT-PCR positive samples from the upper respiratory tract of patients over time. Only Ct values from samples analyzed with the same RT-PCR diagnostic assay were compared. The Chi-Square test was used for testing the association between the vaccination status and frequency of P.1 variants. The threshold for statistical significance was set to *P* < 0.05. Graphics and statistical analyses were performed using GraphPad v9.02 (Prism Software, United States).

### Data availability.

All the SARS-CoV-2 genomes generated and analyzed in this study are available at the EpiCov database in GISAID (https://www.gisaid.org/). The list of accession IDs may be found in the attached supplemental material file.
