# Supplementary material for: Spread of Gamma (P.1) Sub-Lineages Carrying Spike Mutations Close to the Furin Cleavage Site and Deletions in the N-Terminal Domain Drives Ongoing Transmission of SARS-CoV-2 in Amazonas, Brazil
Source: Microbiol Spectr. 2022 Feb 23;10(1):e02366-21. doi: 10.1128/spectrum.02366-21 (PMC8865440; doi:10.1128/spectrum.02366-21)
Supplement: SUPPLEMENTAL FILE 1 — Supplemental material. Download SPECTRUM02366-21_Supp_1_seq11.pdf, PDF file, 1.8 MB [file spectrum02366-21_supp_1_seq11.pdf]

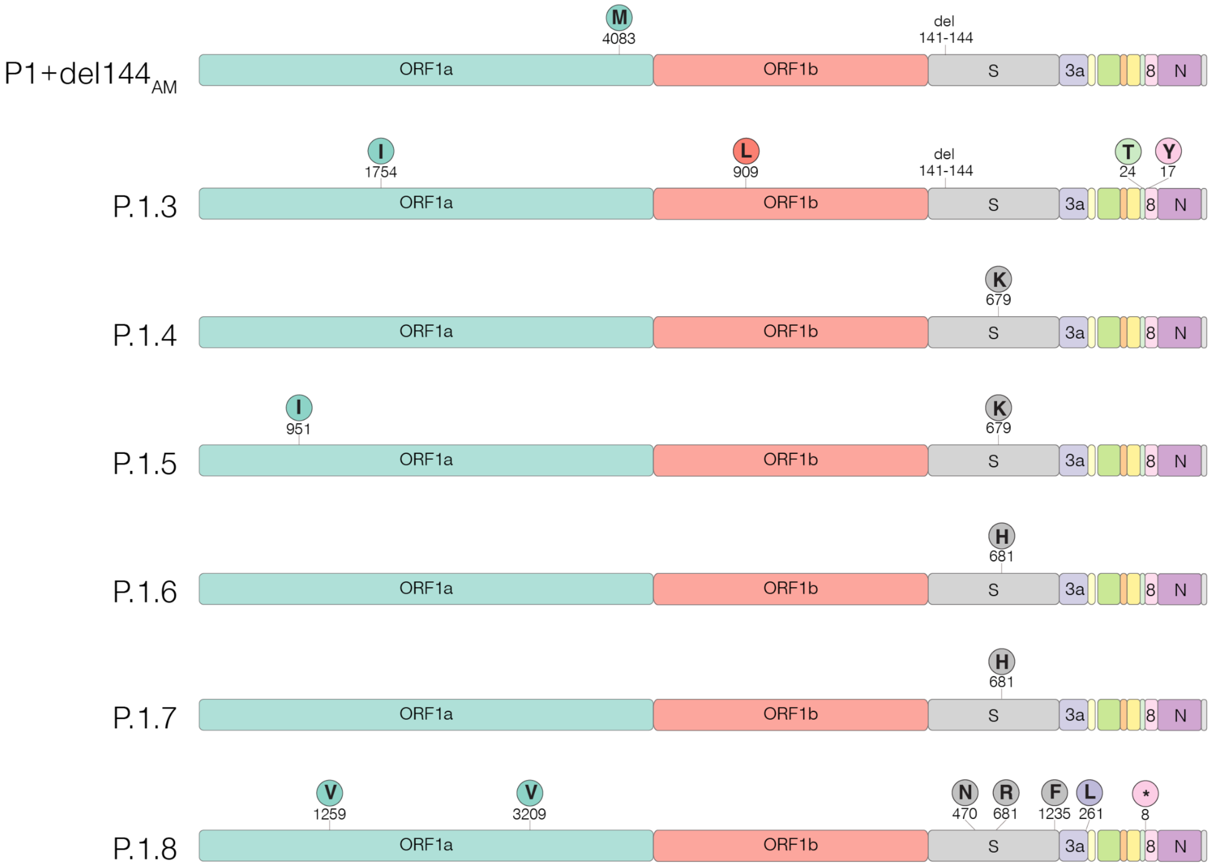

Supplementary Figure 1. Lineage defining mutations of P.1+ lineages. For each lineage, the schematic representation of the genomic organization of SARS-CoV-2 is shown. Mutations were depicted as circles with one-letter amino acid code and the mutation position. Only non-silent mutations are shown, since all mutations are described table 1.

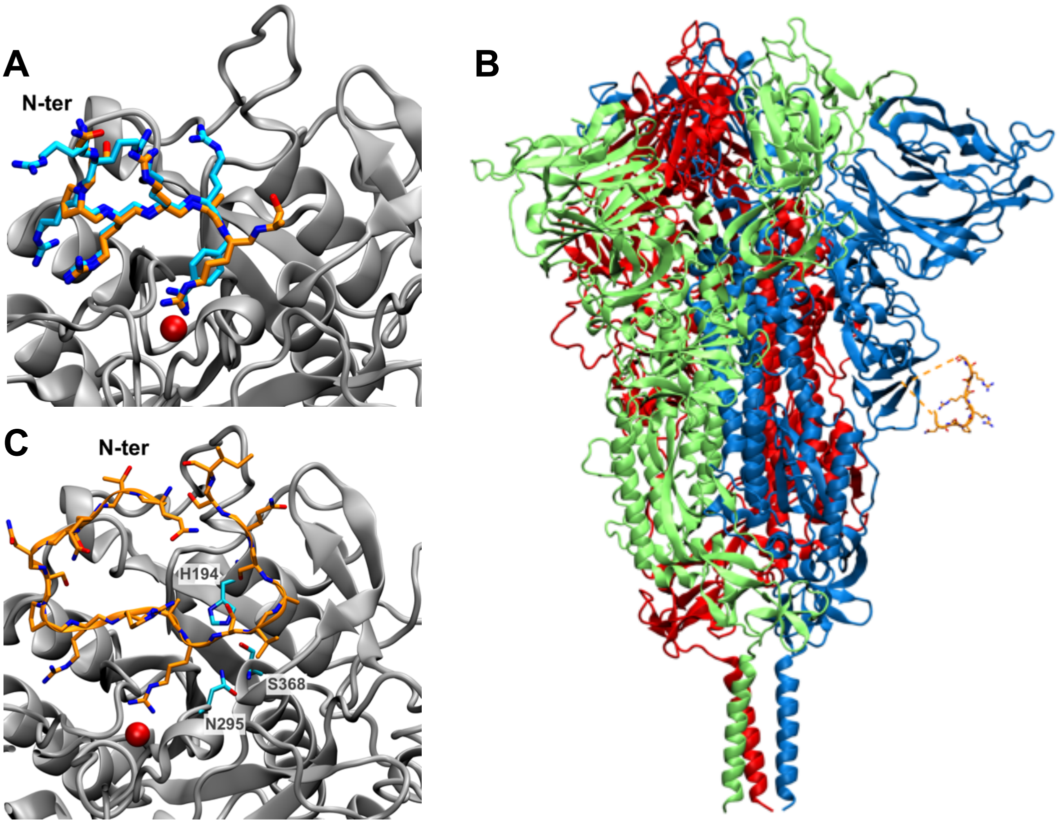

Supplementary Figure 2. A) Structural motif 679-NSPRRARS-686 (orange, licorice representation) of protein S modeled based on an inhibitor (cyan, licorice representation) that was co-crystallized with the furin enzyme (silver, cartoon representation, Ca^{2+} ion in red) (PDB 6HLB). **B)** The modeled 679-NSPRRARS-686 motif (orange, licorice representation) was aligned to the full-Spike homotrimer (chains A, B and C represented in cartoon colors in blue, red and green, respectively) and the remaining residues were modeled to close the loop. **C)** Initial input used in calculations with the loop comprising native residues 675-QTQTNSPRRARSVASQSI-692 (orange, licorice representation) in the conformation of interaction with the furin enzyme (cartoon representation, silver). Active site residues His194, Ser368 and Asn295 are shown in licorice representation (nitrogen atoms in blue, carbon atoms in cyan and oxygen atoms in red) and Ca^{2+} ion is represented as a red sphere.

Supplementary Table 1. Spike Mutations in 1,188 genomes of Gamma or Gamma-like lineages from Amazonas, Brazil (1st January to 6th July 2021).

	JANUARY N = 96				FEBRUARY N = 104				MARCH N = 164				APRIL N = 167				MAY N = 286				JUNE N = 319				JULY N = 52		Total N = 1188	Total
	1-15		16-31		1-15		16-28		1-15		16-31		1-15		16-30		1-15		16-31		1-15		16-30		1-6			
**AA**	**n**	**%**	**n**	**%**	**n**	**%**	**n**	**%**	**n**	**%**	**n**	**%**	**n**	**%**	**n**	**%**	**n**	**%**	**n**	**%**	**n**	**%**	**n**	**%**	**n**	**%**	**n**	**%**
**V3G**	0	0.0	0	0.0	0	0.0	0	0.0	0	0.0	0	0.0	0	0.0	1	0.9	0	0.0	0	0.0	0	0.0	0	0.0	0	0.0	1	0.1
**L5F**	1	1.8	1	2.4	0	0.0	1	2.0	0	0.0	1	1.1	0	0.0	2	1.9	0	0.0	2	1.2	2	1.5	0	0.0	1	1.9	11	0.9
**S12F**	0	0.0	0	0.0	0	0.0	0	0.0	1	1.3	0	0.0	0	0.0	0	0.0	0	0.0	0	0.0	0	0.0	2	1.1	0	0.0	3	0.3
**Q14H**	0	0.0	0	0.0	0	0.0	0	0.0	1	1.3	1	1.1	0	0.0	0	0.0	0	0.0	1	0.6	0	0.0	0	0.0	0	0.0	3	0.3
**L18F**	55	100.0	41	100.0	54	100.0	50	100.0	75	100.0	89	100.0	59	100.0	108	100.0	122	100.0	164	100.0	132	100.0	187	100.0	52	100.0	1188	100.0
**T19I**	0	0.0	0	0.0	0	0.0	0	0.0	0	0.0	1	1.1	0	0.0	0	0.0	0	0.0	0	0.0	0	0.0	0	0.0	0	0.0	1	0.1
**T20N**	55	100.0	40	97.6	54	100.0	50	100.0	75	100.0	87	97.8	59	100.0	108	100.0	122	100.0	164	100.0	132	100.0	187	100.0	52	100.0	1185	99.7
**T22I**	0	0.0	0	0.0	0	0.0	0	0.0	0	0.0	1	1.1	0	0.0	0	0.0	0	0.0	0	0.0	0	0.0	0	0.0	0	0.0	1	0.1
**P25L**	0	0.0	0	0.0	0	0.0	0	0.0	0	0.0	0	0.0	0	0.0	0	0.0	1	0.8	0	0.0	0	0.0	0	0.0	0	0.0	1	0.1
**P25S**	0	0.0	0	0.0	0	0.0	0	0.0	0	0.0	0	0.0	1	1.7	0	0.0	0	0.0	0	0.0	0	0.0	0	0.0	0	0.0	1	0.1
**P26S**	55	100.0	41	100.0	54	100.0	50	100.0	75	100.0	89	100.0	59	100.0	108	100.0	122	100.0	164	100.0	132	100.0	187	100.0	52	100.0	1188	100.0
**T29I**	0	0.0	0	0.0	0	0.0	0	0.0	0	0.0	0	0.0	0	0.0	0	0.0	0	0.0	0	0.0	0	0.0	1	0.5	0	0.0	1	0.1
**V42I**	0	0.0	0	0.0	0	0.0	0	0.0	0	0.0	0	0.0	0	0.0	0	0.0	0	0.0	1	0.6	0	0.0	0	0.0	0	0.0	1	0.1
**H49Y**	0	0.0	0	0.0	0	0.0	0	0.0	1	1.3	3	3.4	2	3.4	8	7.4	9	7.4	1	0.6	0	0.0	0	0.0	0	0.0	24	2.0
**H49L**	0	0.0	0	0.0	0	0.0	0	0.0	0	0.0	1	1.1	0	0.0	0	0.0	0	0.0	0	0.0	0	0.0	0	0.0	0	0.0	1	0.1
**A67S**	0	0.0	0	0.0	0	0.0	0	0.0	0	0.0	0	0.0	0	0.0	2	1.9	2	1.6	1	0.6	3	2.3	3	1.6	1	1.9	12	1.0
**A67T**	0	0.0	0	0.0	0	0.0	0	0.0	0	0.0	0	0.0	0	0.0	1	0.9	0	0.0	0	0.0	0	0.0	0	0.0	0	0.0	1	0.1
**I68V**	0	0.0	0	0.0	0	0.0	0	0.0	0	0.0	0	0.0	0	0.0	0	0.0	2	1.6	0	0.0	0	0.0	0	0.0	0	0.0	2	0.2

**T95I**	0	0.0	0	0.0	1	1.9	0	0.0	0	0.0	0	0.0	0	0.0	0	0.0	0	0.0	0	0.0	0	0.0	0	0.0	0	0.0	1	0.1
**S98F**	0	0.0	0	0.0	0	0.0	0	0.0	0	0.0	0	0.0	0	0.0	0	0.0	1	0.8	0	0.0	0	0.0	0	0.0	0	0.0	1	0.1
**D138Y**	55	100.0	41	100.0	51	94.4	49	98.0	75	100.0	89	100.0	59	100.0	107	99.1	121	99.2	163	99.4	132	100.0	187	100.0	52	100.0	1181	99.4
**L141F**	0	0.0	0	0.0	0	0.0	0	0.0	0	0.0	0	0.0	0	0.0	0	0.0	2	1.6	0	0.0	0	0.0	0	0.0	0	0.0	2	0.2
**Y144D**	0	0.0	0	0.0	1	1.9	0	0.0	0	0.0	0	0.0	0	0.0	0	0.0	0	0.0	0	0.0	0	0.0	0	0.0	0	0.0	1	0.1
**H146Y**	0	0.0	0	0.0	0	0.0	0	0.0	0	0.0	0	0.0	0	0.0	0	0.0	0	0.0	0	0.0	0	0.0	1	0.5	0	0.0	1	0.1
**W152L**	0	0.0	0	0.0	5	9.3	0	0.0	0	0.0	0	0.0	0	0.0	0	0.0	0	0.0	0	0.0	0	0.0	0	0.0	0	0.0	5	0.4
**W152C**	0	0.0	0	0.0	0	0.0	0	0.0	0	0.0	0	0.0	0	0.0	0	0.0	1	0.8	0	0.0	0	0.0	0	0.0	0	0.0	1	0.1
**M153I**	0	0.0	0	0.0	0	0.0	0	0.0	0	0.0	0	0.0	0	0.0	1	0.9	0	0.0	0	0.0	0	0.0	0	0.0	0	0.0	1	0.1
**N165D**	0	0.0	0	0.0	0	0.0	0	0.0	0	0.0	0	0.0	0	0.0	0	0.0	0	0.0	1	0.6	0	0.0	0	0.0	0	0.0	1	0.1
**P174S**	0	0.0	0	0.0	0	0.0	0	0.0	0	0.0	0	0.0	0	0.0	0	0.0	0	0.0	0	0.0	0	0.0	1	0.5	0	0.0	1	0.1
**L176F**	0	0.0	0	0.0	0	0.0	0	0.0	0	0.0	0	0.0	1	1.7	0	0.0	0	0.0	0	0.0	0	0.0	0	0.0	0	0.0	1	0.1
**K182R**	0	0.0	0	0.0	0	0.0	0	0.0	0	0.0	1	1.1	0	0.0	0	0.0	0	0.0	0	0.0	0	0.0	0	0.0	0	0.0	1	0.1
**R190S**	53	96.4	40	97.6	54	100.0	50	100.0	75	100.0	89	100.0	59	100.0	108	100.0	122	100.0	163	99.4	132	100.0	187	100.0	52	100.0	1184	99.7
**V193L**	0	0.0	0	0.0	0	0.0	0	0.0	0	0.0	0	0.0	0	0.0	0	0.0	1	0.8	0	0.0	0	0.0	0	0.0	0	0.0	1	0.1
**N211S**	0	0.0	0	0.0	0	0.0	0	0.0	0	0.0	0	0.0	1	1.7	1	0.9	1	0.8	0	0.0	0	0.0	0	0.0	0	0.0	3	0.3
**D215G**	0	0.0	0	0.0	0	0.0	0	0.0	1	1.3	1	1.1	0	0.0	4	3.7	2	1.6	1	0.6	0	0.0	0	0.0	0	0.0	9	0.8
**D215H**	0	0.0	0	0.0	0	0.0	0	0.0	0	0.0	0	0.0	0	0.0	0	0.0	0	0.0	0	0.0	0	0.0	1	0.5	0	0.0	1	0.1
**L216F**	0	0.0	0	0.0	0	0.0	1	2.0	1	1.3	0	0.0	0	0.0	0	0.0	0	0.0	0	0.0	0	0.0	0	0.0	0	0.0	2	0.2
**G232V**	0	0.0	0	0.0	0	0.0	0	0.0	1	1.3	0	0.0	0	0.0	0	0.0	0	0.0	0	0.0	0	0.0	0	0.0	0	0.0	1	0.1
**N234K**	0	0.0	0	0.0	0	0.0	0	0.0	0	0.0	0	0.0	0	0.0	0	0.0	0	0.0	1	0.6	0	0.0	0	0.0	0	0.0	1	0.1
**A243S**	0	0.0	0	0.0	0	0.0	0	0.0	0	0.0	1	1.1	3	5.1	0	0.0	0	0.0	0	0.0	0	0.0	0	0.0	0	0.0	4	0.3
**H245Y**	0	0.0	0	0.0	0	0.0	0	0.0	0	0.0	0	0.0	0	0.0	0	0.0	0	0.0	0	0.0	1	0.8	0	0.0	0	0.0	1	0.1
**P251L**	0	0.0	0	0.0	0	0.0	0	0.0	0	0.0	0	0.0	0	0.0	0	0.0	0	0.0	0	0.0	0	0.0	0	0.0	1	1.9	1	0.1

**S256L**	0	0.0	0	0.0	0	0.0	0	0.0	1	1.3	0	0.0	0	0.0	0	0.0	0	0.0	0	0.0	0	0.0	0	0.0	0	0.0	1	0.1
**G257D**	0	0.0	0	0.0	0	0.0	0	0.0	0	0.0	0	0.0	1	1.7	0	0.0	0	0.0	0	0.0	0	0.0	1	0.5	0	0.0	2	0.2
**T259I**	0	0.0	0	0.0	0	0.0	0	0.0	1	1.3	0	0.0	0	0.0	0	0.0	0	0.0	0	0.0	0	0.0	0	0.0	0	0.0	1	0.1
**T259A**	0	0.0	0	0.0	0	0.0	0	0.0	0	0.0	0	0.0	0	0.0	0	0.0	2	1.6	0	0.0	2	1.5	0	0.0	0	0.0	4	0.3
**L303F**	0	0.0	0	0.0	0	0.0	0	0.0	0	0.0	0	0.0	0	0.0	0	0.0	0	0.0	0	0.0	1	0.8	0	0.0	0	0.0	1	0.1
**T323I**	0	0.0	0	0.0	1	1.9	0	0.0	0	0.0	0	0.0	0	0.0	0	0.0	0	0.0	0	0.0	0	0.0	0	0.0	0	0.0	1	0.1
**A363V**	0	0.0	0	0.0	0	0.0	0	0.0	0	0.0	0	0.0	0	0.0	0	0.0	0	0.0	0	0.0	0	0.0	0	0.0	1	1.9	1	0.1
**V367F**	0	0.0	0	0.0	0	0.0	1	2.0	0	0.0	0	0.0	0	0.0	0	0.0	0	0.0	0	0.0	0	0.0	0	0.0	0	0.0	1	0.1
**G413V**	0	0.0	0	0.0	1	1.9	0	0.0	0	0.0	0	0.0	0	0.0	0	0.0	0	0.0	0	0.0	0	0.0	0	0.0	0	0.0	1	0.1
**K417T**	55	100.0	41	100.0	54	100.0	50	100.0	75	100.0	89	100.0	59	100.0	108	100.0	122	100.0	164	100.0	132	100.0	187	100.0	52	100.0	1188	100.0
**K444N**	0	0.0	0	0.0	0	0.0	0	0.0	1	1.3	0	0.0	0	0.0	0	0.0	0	0.0	0	0.0	0	0.0	0	0.0	0	0.0	1	0.1
**T470N**	0	0.0	0	0.0	0	0.0	0	0.0	0	0.0	0	0.0	0	0.0	0	0.0	0	0.0	0	0.0	1	0.8	0	0.0	0	0.0	1	0.1
**E484K**	54	98.2	41	100.0	54	100.0	50	100.0	75	100.0	89	100.0	59	100.0	108	100.0	122	100.0	164	100.0	132	100.0	187	100.0	52	100.0	1187	99.9
**S494P**	0	0.0	0	0.0	0	0.0	0	0.0	0	0.0	0	0.0	0	0.0	0	0.0	0	0.0	0	0.0	0	0.0	1	0.5	0	0.0	1	0.1
**N501Y**	54	98.2	41	100.0	54	100.0	50	100.0	75	100.0	89	100.0	59	100.0	108	100.0	122	100.0	164	100.0	132	100.0	187	100.0	52	100.0	1187	99.9
**Y508H**	0	0.0	0	0.0	0	0.0	0	0.0	0	0.0	0	0.0	0	0.0	1	0.9	0	0.0	0	0.0	0	0.0	0	0.0	0	0.0	1	0.1
**K537N**	0	0.0	0	0.0	0	0.0	0	0.0	0	0.0	0	0.0	0	0.0	0	0.0	0	0.0	2	1.2	1	0.8	1	0.5	0	0.0	4	0.3
**N540S**	0	0.0	0	0.0	0	0.0	0	0.0	0	0.0	1	1.1	0	0.0	0	0.0	0	0.0	0	0.0	0	0.0	0	0.0	0	0.0	1	0.1
**F543L**	0	0.0	0	0.0	0	0.0	0	0.0	0	0.0	0	0.0	0	0.0	0	0.0	2	1.6	0	0.0	0	0.0	0	0.0	0	0.0	2	0.2
**T547K**	0	0.0	0	0.0	0	0.0	0	0.0	1	1.3	0	0.0	0	0.0	0	0.0	0	0.0	0	0.0	0	0.0	2	1.1	0	0.0	3	0.3
**T549I**	0	0.0	0	0.0	0	0.0	0	0.0	1	1.3	0	0.0	0	0.0	0	0.0	0	0.0	0	0.0	0	0.0	0	0.0	0	0.0	1	0.1
**T553I**	1	1.8	0	0.0	0	0.0	0	0.0	0	0.0	1	1.1	0	0.0	0	0.0	0	0.0	0	0.0	0	0.0	0	0.0	0	0.0	2	0.2
**E554D**	0	0.0	0	0.0	0	0.0	0	0.0	0	0.0	0	0.0	0	0.0	0	0.0	0	0.0	0	0.0	2	1.5	0	0.0	0	0.0	2	0.2
**A570V**	0	0.0	0	0.0	0	0.0	1	2.0	0	0.0	0	0.0	0	0.0	0	0.0	0	0.0	0	0.0	0	0.0	0	0.0	0	0.0	1	0.1

**T572I**	0	0.0	0	0.0	0	0.0	0	0.0	0	0.0	0	0.0	0	0.0	0	0.0	0	0.0	0	0.0	0	0.0	1	0.5	0	0.0	1	0.1
**E583G**	0	0.0	0	0.0	0	0.0	0	0.0	0	0.0	0	0.0	0	0.0	0	0.0	0	0.0	0	0.0	1	0.8	0	0.0	0	0.0	1	0.1
**P589Q**	0	0.0	0	0.0	0	0.0	0	0.0	0	0.0	0	0.0	0	0.0	0	0.0	0	0.0	0	0.0	0	0.0	1	0.5	0	0.0	1	0.1
**Q607H**	0	0.0	0	0.0	0	0.0	0	0.0	0	0.0	0	0.0	0	0.0	0	0.0	1	0.8	0	0.0	0	0.0	0	0.0	0	0.0	1	0.1
**Q613H**	0	0.0	0	0.0	0	0.0	0	0.0	0	0.0	0	0.0	0	0.0	1	0.9	0	0.0	0	0.0	0	0.0	0	0.0	0	0.0	1	0.1
**Q613E**	0	0.0	0	0.0	0	0.0	0	0.0	0	0.0	0	0.0	0	0.0	0	0.0	0	0.0	0	0.0	0	0.0	0	0.0	2	3.8	2	0.2
**D614G**	55	100.0	41	100.0	54	100.0	50	100.0	75	100.0	89	100.0	59	100.0	108	100.0	122	100.0	164	100.0	132	100.0	187	100.0	52	100.0	1188	100.0
**E619K**	0	0.0	0	0.0	0	0.0	0	0.0	0	0.0	0	0.0	1	1.7	0	0.0	0	0.0	0	0.0	0	0.0	0	0.0	0	0.0	1	0.1
**V622F**	0	0.0	0	0.0	0	0.0	0	0.0	0	0.0	0	0.0	0	0.0	0	0.0	0	0.0	0	0.0	0	0.0	1	0.5	0	0.0	1	0.1
**D627Y**	0	0.0	0	0.0	0	0.0	0	0.0	0	0.0	0	0.0	0	0.0	0	0.0	0	0.0	0	0.0	2	1.5	0	0.0	0	0.0	2	0.2
**A653V**	0	0.0	0	0.0	0	0.0	0	0.0	0	0.0	1	1.1	0	0.0	0	0.0	0	0.0	0	0.0	0	0.0	1	0.5	0	0.0	2	0.2
**E654A**	0	0.0	0	0.0	0	0.0	0	0.0	0	0.0	0	0.0	0	0.0	0	0.0	0	0.0	0	0.0	0	0.0	0	0.0	1	1.9	1	0.1
**H655Y**	55	100.0	40	97.6	54	100.0	50	100.0	75	100.0	89	100.0	59	100.0	108	100.0	122	100.0	164	100.0	132	100.0	187	100.0	52	100.0	1187	99.9
**S659L**	0	0.0	0	0.0	1	1.9	0	0.0	0	0.0	0	0.0	0	0.0	0	0.0	0	0.0	0	0.0	0	0.0	0	0.0	0	0.0	1	0.1
**Q675H**	0	0.0	0	0.0	0	0.0	0	0.0	0	0.0	0	0.0	0	0.0	2	1.9	0	0.0	0	0.0	2	1.5	0	0.0	0	0.0	4	0.3
**N679K**	0	0.0	0	0.0	0	0.0	0	0.0	0	0.0	3	3.4	7	11.9	24	22.2	21	17.2	53	32.3	54	40.9	100	53.5	40	76.9	302	25.4
**P681H**	0	0.0	0	0.0	0	0.0	0	0.0	0	0.0	1	1.1	4	6.8	14	13.0	25	20.5	48	29.3	49	37.1	61	32.6	7	13.5	209	17.6
**P681T**	0	0.0	0	0.0	0	0.0	0	0.0	0	0.0	0	0.0	0	0.0	1	0.9	0	0.0	0	0.0	0	0.0	0	0.0	0	0.0	1	0.1
**P681R**	0	0.0	0	0.0	0	0.0	0	0.0	0	0.0	0	0.0	0	0.0	0	0.0	0	0.0	0	0.0	1	0.8	0	0.0	0	0.0	1	0.1
**T719I**	0	0.0	0	0.0	0	0.0	0	0.0	0	0.0	0	0.0	0	0.0	0	0.0	0	0.0	0	0.0	0	0.0	1	0.5	0	0.0	1	0.1
**E725D**	0	0.0	0	0.0	0	0.0	0	0.0	0	0.0	0	0.0	0	0.0	2	1.9	0	0.0	0	0.0	0	0.0	0	0.0	0	0.0	2	0.2
**T732I**	0	0.0	0	0.0	0	0.0	0	0.0	0	0.0	0	0.0	0	0.0	0	0.0	0	0.0	0	0.0	0	0.0	1	0.5	0	0.0	1	0.1
**I770V**	0	0.0	0	0.0	0	0.0	0	0.0	0	0.0	0	0.0	0	0.0	0	0.0	0	0.0	1	0.6	0	0.0	0	0.0	0	0.0	1	0.1
**Q779K**	0	0.0	0	0.0	0	0.0	0	0.0	0	0.0	0	0.0	0	0.0	1	0.9	0	0.0	0	0.0	0	0.0	0	0.0	0	0.0	1	0.1

**E780D**	1	1.8	0	0.0	0	0.0	0	0.0	0	0.0	0	0.0	0	0.0	0	0.0	0	0.0	0	0.0	0	0.0	0	0.0	0	0.0	1	0.1
**P809L**	0	0.0	0	0.0	0	0.0	0	0.0	0	0.0	0	0.0	0	0.0	0	0.0	0	0.0	0	0.0	0	0.0	1	0.5	0	0.0	1	0.1
**P812L**	0	0.0	0	0.0	1	1.9	0	0.0	0	0.0	0	0.0	0	0.0	0	0.0	0	0.0	0	0.0	0	0.0	0	0.0	0	0.0	1	0.1
**D839G**	0	0.0	0	0.0	0	0.0	1	2.0	0	0.0	0	0.0	0	0.0	0	0.0	0	0.0	0	0.0	0	0.0	0	0.0	0	0.0	1	0.1
**A852V**	0	0.0	0	0.0	0	0.0	0	0.0	0	0.0	0	0.0	0	0.0	0	0.0	1	0.8	1	0.6	0	0.0	0	0.0	0	0.0	2	0.2
**T859I**	0	0.0	0	0.0	0	0.0	0	0.0	0	0.0	1	1.1	0	0.0	0	0.0	0	0.0	0	0.0	0	0.0	0	0.0	0	0.0	1	0.1
**V860L**	0	0.0	0	0.0	0	0.0	0	0.0	0	0.0	1	1.1	0	0.0	0	0.0	0	0.0	0	0.0	0	0.0	0	0.0	0	0.0	1	0.1
**I870V**	0	0.0	0	0.0	0	0.0	0	0.0	2	2.7	0	0.0	0	0.0	0	0.0	0	0.0	0	0.0	0	0.0	0	0.0	0	0.0	2	0.2
**A871V**	0	0.0	0	0.0	0	0.0	1	2.0	0	0.0	0	0.0	0	0.0	0	0.0	0	0.0	0	0.0	0	0.0	0	0.0	0	0.0	1	0.1
**T883I**	0	0.0	0	0.0	0	0.0	1	2.0	0	0.0	0	0.0	0	0.0	0	0.0	0	0.0	0	0.0	0	0.0	0	0.0	0	0.0	1	0.1
**S939F**	0	0.0	0	0.0	0	0.0	0	0.0	0	0.0	0	0.0	0	0.0	0	0.0	0	0.0	0	0.0	0	0.0	1	0.5	0	0.0	1	0.1
**A942V**	0	0.0	0	0.0	0	0.0	0	0.0	0	0.0	0	0.0	0	0.0	0	0.0	0	0.0	0	0.0	0	0.0	1	0.5	0	0.0	1	0.1
**Q954L**	0	0.0	0	0.0	0	0.0	0	0.0	0	0.0	0	0.0	0	0.0	0	0.0	0	0.0	0	0.0	0	0.0	1	0.5	1	1.9	2	0.2
**A1020V**	0	0.0	0	0.0	0	0.0	0	0.0	0	0.0	0	0.0	0	0.0	0	0.0	0	0.0	0	0.0	1	0.8	0	0.0	1	1.9	2	0.2
**T1027I**	55	100.0	40	97.6	54	100.0	50	100.0	75	100.0	89	100.0	59	100.0	108	100.0	122	100.0	164	100.0	132	100.0	187	100.0	52	100.0	1187	99.9
**A1078S**	0	0.0	0	0.0	0	0.0	1	2.0	0	0.0	0	0.0	0	0.0	0	0.0	0	0.0	0	0.0	0	0.0	0	0.0	0	0.0	1	0.1
**D1084Y**	0	0.0	0	0.0	0	0.0	1	2.0	0	0.0	0	0.0	0	0.0	0	0.0	0	0.0	0	0.0	0	0.0	0	0.0	0	0.0	1	0.1
**R1091H**	0	0.0	0	0.0	0	0.0	0	0.0	0	0.0	0	0.0	0	0.0	0	0.0	1	0.8	1	0.6	0	0.0	0	0.0	0	0.0	2	0.2
**T1117I**	0	0.0	0	0.0	0	0.0	0	0.0	1	1.3	0	0.0	0	0.0	0	0.0	0	0.0	0	0.0	0	0.0	0	0.0	0	0.0	1	0.1
**D1139H**	0	0.0	1	2.4	0	0.0	0	0.0	0	0.0	0	0.0	0	0.0	0	0.0	0	0.0	0	0.0	0	0.0	0	0.0	0	0.0	1	0.1
**D1153Y**	0	0.0	2	4.9	0	0.0	0	0.0	0	0.0	0	0.0	0	0.0	0	0.0	0	0.0	0	0.0	0	0.0	0	0.0	0	0.0	2	0.2
**D1168Y**	0	0.0	0	0.0	0	0.0	0	0.0	1	1.3	0	0.0	0	0.0	0	0.0	0	0.0	0	0.0	0	0.0	0	0.0	0	0.0	1	0.1
**G1171V**	0	0.0	0	0.0	0	0.0	0	0.0	0	0.0	0	0.0	0	0.0	0	0.0	1	0.8	2	1.2	0	0.0	0	0.0	0	0.0	3	0.3
**V1176F**	55	100.0	41	100.0	54	100.0	50	100.0	75	100.0	89	100.0	59	100.0	108	100.0	122	100.0	164	100.0	132	100.0	187	100.0	52	100.0	1188	100.0

**E1182Q**	0	0.0	0	0.0	0	0.0	0	0.0	0	0.0	0	0.0	0	0.0	0	0.0	0	0.0	0	0.0	0	0.0	0	0.0	1	1.9	1	0.1
**L1234F**	0	0.0	1	2.4	1	1.9	0	0.0	0	0.0	0	0.0	0	0.0	0	0.0	0	0.0	0	0.0	0	0.0	0	0.0	0	0.0	2	0.2
**C1235F**	0	0.0	0	0.0	0	0.0	0	0.0	0	0.0	0	0.0	0	0.0	0	0.0	0	0.0	0	0.0	1	0.8	0	0.0	0	0.0	1	0.1
**S1252F**	0	0.0	0	0.0	0	0.0	0	0.0	0	0.0	1	1.1	0	0.0	0	0.0	0	0.0	0	0.0	0	0.0	0	0.0	0	0.0	1	0.1
**E1258D**	0	0.0	0	0.0	0	0.0	0	0.0	0	0.0	0	0.0	2	3.4	0	0.0	0	0.0	0	0.0	0	0.0	0	0.0	0	0.0	2	0.2
**D1259Y**	0	0.0	0	0.0	0	0.0	0	0.0	0	0.0	1	1.1	0	0.0	0	0.0	0	0.0	0	0.0	0	0.0	0	0.0	0	0.0	1	0.1
**S1261F**	0	0.0	0	0.0	0	0.0	0	0.0	0	0.0	0	0.0	0	0.0	0	0.0	0	0.0	1	0.6	0	0.0	0	0.0	0	0.0	1	0.1

Legend. Spike mutations (AA) are represented by the wild-type amino acid (1-letter code) - residue position - and the mutant amino acid. The total number (N) of genomes generated each month. as well as the number (n) and the percentage (%) of each mutation at different time intervals. are shown. More than one mutant residue was observed in eight positions (P25L or P25S. H49Y or H49L. A67S or A67T. W152L or W152C. D215G or D215H. T259I or T259A. Q613H or Q613E. and P681H or P681T or P681R). Four mutations at the furin cleavage site were observed. N679K in 302 genomes. P681H in 209 genomes. P681R and P681T found in one genome each. The spike mutations N679K and P681H showed a sharp increase from 3.4% (N679K) and 1.1% (P681H) in late March to 76.9% (N679K) and 13.5% (P681H) in early July. Together. genomes carrying furin site mutations (N679K or P681H) represent more than 90% of those sequenced in July.

Supplementary Table 2. Spike mutations of VOC Omicron among Gamma sequences in Brazil.

Mutation	Gamma AM	Gamma Other states	Lineage
A67V	-	15	P.1/P.1.14/P.1.16
H69-del	-	85	P.1/P.1.1/P.1.12/P.1.14
V70-del	-	86	P.1/P.1.1/P.1.12/P.1.14
T95I	1	50	P.1/P.1.7/P.1.12/P.1.14
G142-del<br>G142D	45<br>2	74<br>4	P.1/P.1.2/P.1.3/P.1.4/P.1.6/P.1.11/P.1.14<br>P.1/P.1.6/P.1.14
V143-del	47	79	P.1/P.1.2/P.1.3/P.1.4/P.1.6/P.1.11/P.1.14
Y144-del	80	277	P.1/P.1.1/P.1.2/P.1.3/P.1.4/P.1.6/P.1.7/ P.1.8/P.1.10/P.1.12/P.1.14
Y145D<br>Y145del	-<br>2	-<br>21	-<br>P.1/P.1.6/P.1.7/P.1.11/P.1.14
N211-del	-	1	P.1
L212I	-	2	P.1/P.1.7
ins214-EPE	3 (ANRN)	5 (ANRN)	P.1
G339D	-	7	P.1
S371L	-	-	-
S373P	-	13	P.1/P.1.7/P.1.14
S375F	-	-	-
K417N	K417T	K417T	All P.1 and P.1+
N440K	-	-	-
G446S	1	2	P.1
S477N	-	4	P.1/P.1.7/P.1.14
T478K	-	10	P.1/P.1.7
E484A	E484K	E484K	All P.1 and P.1+
Q493R	-	1	P.1
G496S	1	1	P.1/P.1.6
Q498R	1	-	P.1.4
N501Y	N501Y	N501Y	All P.1 and P.1+
Y505H	-	6	P.1/P.1.14
T547K	5	-	P.1.4
D614G	D614G	D614G	All P.1 and P.1+
H655Y	H655Y	H655Y	All P.1 and P.1+
N679K	943	108	P.1/P.1.4/P.1.5/P.1.10/P.1.12
P681H	451	3,253	P.1/P.1.1/P.1.6/P.1.7/ P.1.12/P.1.13/P.1.14/P.1.16
N764K	-	-	-
D796Y	-	11	P.1/P.1.7/P.1.14/P.1.15
N856K	-	1	P.1
Q954H	-	-	-
N969K	-	2	P.1
L981F	-	-	-

Total Gamma BR (09/12/2021) = 45,772

SUPPLEMENTARY INFORMATION MATERIAL

Atomistic Simulations Additional Information

Prior to the metadynamics simulations, all systems were equilibrated by means of a 5-ns molecular dynamics (MD) simulation. The classical atomistic simulations were performed for the furin enzyme complexed to the wild-type SARS-CoV-2 Spike protein loop, and N679K, P681H and P681R variants, modeled as described above and with the N/C-termini capped. The complexes were embedded in the center of an orthorhombic box with edge dimensions of 2.0 nm of distance from the center of the solute. The systems were solvated with explicit solvent molecules described by the SPC water model[1]. Sodium and chloride ions were added to neutralize each system's total charge while reproducing a buffer of saline solution at 150 nM. The systems were initially energy minimized using 10,000 steps of the steepest descent algorithm. Periodic boundary conditions in were applied in the x, y, and z directions. Holonomic constraints were applied to the bond lengths involving hydrogen atoms in the solute using the LINCS algorithm[2], allowing a 2.0 fs integration time step, in which the leap-frog algorithm was used to integrate the equations of motion. Short-range electrostatics and van der Waals interactions were calculated within the cutoff radius of 1.4 nm. Long-range electrostatics corrections were taken into account by using the reaction-field[3] method beyond a cutoff of 1.4 nm in conjunction with a permittivity dielectric constant of 66. All MD simulations were carried out using the GROMOS parameter set 54A7[4] for the protein, and the GROMOS 53A6[5] parameter set for the ions, within the GROMACS 4.6.7[6]. The equilibration was conducted in the NVT ensemble (constant number of particles, volume, and temperature). The reference temperature was kept at 310 K separately coupling separate v-rescale thermostats[7] for the solute and solvent (electrolytes included) with a relaxation time of 1.0 ps. The systems were previously thermalized by generating initial velocities from a Maxwell-Boltzmann distribution starting at 5 K and progressively increasing to the reference temperature. The simulations were carried out with a 1,000 $kJ.mol^{-1}$ force constant applied to the heavy-backbone atoms of the proteins, allowing only the rearrangement of the water molecules around the solute.

The last frame of the previously equilibrated restrained MD simulations was used as the starting point to the metadynamics. The peptides were steered from their binding sites by defining the collective variables (CV) as the distance between the center of mass of the peptide's α-carbons and the center of mass of the α-carbons of the furin residues interacting initially at 3 Å distant from the peptide. For metadynamics simulations the positional restraints were released, except for the coordinated-Ca^{2+} ions in the furin, in which a 1,000 $kJ.mol^{-1}$ force was applied to restrain their position to the coordinated residues. The

exploration of the CV phase space was accomplished by adding Gaussian potentials of height 0.05 kJ/mol and depth of 0.05 nm every 1.0 ps. CVs sampling and calculations were performed for 50 ns via the PLUMED 2.3.5 plugin[8] interfaced with the GROMACS v. 4.6.7. The simulations setup for the metadynamics was the same as described for the restrained MD simulations, save for the inclusion of the Parrinello-Rahman (PR) barostat scheme[9, 10] with a relaxation time of 2.0 ps, leading to simulations in the isothermical-isobaric ensemble (NpT). The choice of PR method for the metadynamics calculations, despite the second-order approach to equilibrium, relies on its correct reproduction of the exact NpT ensemble, which is more appropriate when obtaining thermodynamic quantities. The peptides were detached from the protein along the CV pathway, and the free energy surface (FES) of the process was recursively reconstructed using the sum_hills tool.

References

1. Berendsen, H. J. C.; Postma, J. P. M.; Van Gunsteren, W. F.; Hermans, a. J., Intermolecular Forces. Reidel, Dordrecht Jerusalem, Israel: 1981.
2. Hess, B.; Bekker, H.; Berendsen, H. J. C.; Fraaije, J. G. E. M., Lincs: A Linear Constraint Solver for Molecular Simulations. *Journal of computational chemistry* **1997,** *18*, 1463-1472.
3. Tironi, I. G.; Sperb, R.; Smith, P. E.; van Gunsteren, W. F., A Generalized Reaction Field Method for Molecular Dynamics Simulations. *The Journal of Chemical Physics* **1995,** *102*, 5451-5459.
4. Schmid, N.; Eichenberger, A. P.; Choutko, A.; Riniker, S.; Winger, M.; Mark, A. E.; van Gunsteren, W. F., Definition and Testing of the Gromos Force-Field Versions 54a7 and 54b7. *European biophysics journal : EBJ* **2011,** *40*, 843-856.
5. Oostenbrink, C.; Villa, A.; Mark, A. E.; van Gunsteren, W. F., A Biomolecular Force Field Based on the Free Enthalpy of Hydration and Solvation: The Gromos Force-Field Parameter Sets 53a5 and 53a6. *J Comput Chem* **2004,** *25*, 1656-1676.
6. Van Der Spoel, D.; Lindahl, E.; Hess, B.; Groenhof, G.; Mark, A. E.; Berendsen, H. J., Gromacs: Fast, Flexible, and Free. *J Comput Chem* **2005,** *26*, 1701-1718.
7. Bussi, G.; Donadio, D.; Parrinello, M., Canonical Sampling through Velocity Rescaling. *The Journal of chemical physics* **2007,** *126*, 014101.
8. Tribello, G. A.; Bonomi, M.; Branduardi, D.; Camilloni, C.; Bussi, G., Plumed 2: New Feathers for an Old Bird. *Computer Physics Communications* **2014,** *185*, 604-613.
9. Parrinello, M.; Rahman, A., Polymorphic Transitions in Single Crystals: A New Molecular Dynamics Method. *Journal of Applied Physics* **1981,** *52*, 7182-7190.
10. Parrinello, M.; Rahman, A., Crystal Structure and Pair Potentials: A Molecular-Dynamics Study. *Physical Review Letters* **1980,** *45*, 1196-1199.

SARS-COV-2 genomes generated in this study

EPI_ISL_3050301	EPI_ISL_2777407	EPI_ISL_2777474	EPI_ISL_2777510	EPI_ISL_2777599
EPI_ISL_2777325	EPI_ISL_2777408	EPI_ISL_2777475	EPI_ISL_2777238	EPI_ISL_2777600
EPI_ISL_2777615	EPI_ISL_2777409	EPI_ISL_2777476	EPI_ISL_2777511	EPI_ISL_2777601
EPI_ISL_2777433	EPI_ISL_1034304	EPI_ISL_2777477	EPI_ISL_2777512	EPI_ISL_2777603
EPI_ISL_2777434	EPI_ISL_2777410	EPI_ISL_2777478	EPI_ISL_2777513	EPI_ISL_2777604
EPI_ISL_2777436	EPI_ISL_2777411	EPI_ISL_2777479	EPI_ISL_2777357	EPI_ISL_2777605
EPI_ISL_2777437	EPI_ISL_2777412	EPI_ISL_2777480	EPI_ISL_2777358	EPI_ISL_2777606
EPI_ISL_2777438	EPI_ISL_2777413	EPI_ISL_2777481	EPI_ISL_2777515	EPI_ISL_2777609
EPI_ISL_2777439	EPI_ISL_2777414	EPI_ISL_2777482	EPI_ISL_2777516	EPI_ISL_2777248
EPI_ISL_2777440	EPI_ISL_2777415	EPI_ISL_2777483	EPI_ISL_2777517	EPI_ISL_2777612
EPI_ISL_2777441	EPI_ISL_1034306	EPI_ISL_2777484	EPI_ISL_2777518	EPI_ISL_2777616
EPI_ISL_2777443	EPI_ISL_2777416	EPI_ISL_2777485	EPI_ISL_2777989	EPI_ISL_2777618
EPI_ISL_2777444	EPI_ISL_2777417	EPI_ISL_2777486	EPI_ISL_2777990	EPI_ISL_2777620
EPI_ISL_2777445	EPI_ISL_2777418	EPI_ISL_2777487	EPI_ISL_2777519	EPI_ISL_2777621
EPI_ISL_2777446	EPI_ISL_2777419	EPI_ISL_2778003	EPI_ISL_2777520	EPI_ISL_2777622
EPI_ISL_2777448	EPI_ISL_2777420	EPI_ISL_2777488	EPI_ISL_2777521	EPI_ISL_2777624
EPI_ISL_2777449	EPI_ISL_2777421	EPI_ISL_2777489	EPI_ISL_2777522	EPI_ISL_2777627
EPI_ISL_2777450	EPI_ISL_2777422	EPI_ISL_2777491	EPI_ISL_2777992	EPI_ISL_2777980
EPI_ISL_2777451	EPI_ISL_2777423	EPI_ISL_2777491	EPI_ISL_1068291	EPI_ISL_2777628
EPI_ISL_2777452	EPI_ISL_2777424	EPI_ISL_2778001	EPI_ISL_2777524	EPI_ISL_2777630
EPI_ISL_2777490	EPI_ISL_2777425	EPI_ISL_2778001	EPI_ISL_2777525	EPI_ISL_2777631
EPI_ISL_2777311	EPI_ISL_2777426	EPI_ISL_2777492	EPI_ISL_2777526	EPI_ISL_2777632
EPI_ISL_2777314	EPI_ISL_2777427	EPI_ISL_2777492	EPI_ISL_2777527	EPI_ISL_2777634
EPI_ISL_2777547	EPI_ISL_2777428	EPI_ISL_2777493	EPI_ISL_2778002	EPI_ISL_2777635
EPI_ISL_2777626	EPI_ISL_2777429	EPI_ISL_2777494	EPI_ISL_2777528	EPI_ISL_2777636
EPI_ISL_2777650	EPI_ISL_2777430	EPI_ISL_1068279	EPI_ISL_2777530	EPI_ISL_2777637
EPI_ISL_2777678	EPI_ISL_2777430	EPI_ISL_1068280	EPI_ISL_1068292	EPI_ISL_2777638
EPI_ISL_2777689	EPI_ISL_2777431	EPI_ISL_1068281	EPI_ISL_2777532	EPI_ISL_2777639
EPI_ISL_2777741	EPI_ISL_2777431	EPI_ISL_1068282	EPI_ISL_2777533	EPI_ISL_2777641
EPI_ISL_2777851	EPI_ISL_2777432	EPI_ISL_1068283	EPI_ISL_2777534	EPI_ISL_2777642
EPI_ISL_2777937	EPI_ISL_2777432	EPI_ISL_1068284	EPI_ISL_2777535	EPI_ISL_2777643
EPI_ISL_2777947	EPI_ISL_2777995	EPI_ISL_1068285	EPI_ISL_2777536	EPI_ISL_2777645
EPI_ISL_3050601	EPI_ISL_2777996	EPI_ISL_2777495	EPI_ISL_2777537	EPI_ISL_2777646
EPI_ISL_1533609	EPI_ISL_2777997	EPI_ISL_1068286	EPI_ISL_2777538	EPI_ISL_2777647
EPI_ISL_2777509	EPI_ISL_2777998	EPI_ISL_2778004	EPI_ISL_2777539	EPI_ISL_2777651
EPI_ISL_2777356	EPI_ISL_2777999	EPI_ISL_2778005	EPI_ISL_2777540	EPI_ISL_2777654
EPI_ISL_2777557	EPI_ISL_2778000	EPI_ISL_2777239	EPI_ISL_2777541	EPI_ISL_2777655
EPI_ISL_2777562	EPI_ISL_2777435	EPI_ISL_2777236	EPI_ISL_2777542	EPI_ISL_2777657
EPI_ISL_2777564	EPI_ISL_2777453	EPI_ISL_2777301	EPI_ISL_2777543	EPI_ISL_2777658
EPI_ISL_2777591	EPI_ISL_1068258	EPI_ISL_2777240	EPI_ISL_2777544	EPI_ISL_2777659
EPI_ISL_2777669	EPI_ISL_1068260	EPI_ISL_1068287	EPI_ISL_2777545	EPI_ISL_2777662
EPI_ISL_2777690	EPI_ISL_1068261	EPI_ISL_1068288	EPI_ISL_2777546	EPI_ISL_2777668
EPI_ISL_2777704	EPI_ISL_1068262	EPI_ISL_2777241	EPI_ISL_2777549	EPI_ISL_2777670
EPI_ISL_2777327	EPI_ISL_1068263	EPI_ISL_2777246	EPI_ISL_2777551	EPI_ISL_2777675
EPI_ISL_2777765	EPI_ISL_1068264	EPI_ISL_2777242	EPI_ISL_2777553	EPI_ISL_2777676
EPI_ISL_2777875	EPI_ISL_1068266	EPI_ISL_1068289	EPI_ISL_2777554	EPI_ISL_2777683
EPI_ISL_2777892	EPI_ISL_1068267	EPI_ISL_2777496	EPI_ISL_2777556	EPI_ISL_2777686
EPI_ISL_2777898	EPI_ISL_1068268	EPI_ISL_2777496	EPI_ISL_2777558	EPI_ISL_2777687
EPI_ISL_2777934	EPI_ISL_1068269	EPI_ISL_2777496	EPI_ISL_2777559	EPI_ISL_2777691
EPI_ISL_2777963	EPI_ISL_1068270	EPI_ISL_1068290	EPI_ISL_2777560	EPI_ISL_2777692
EPI_ISL_2777299	EPI_ISL_1068271	EPI_ISL_2777498	EPI_ISL_2777561	EPI_ISL_2777693
EPI_ISL_3050363	EPI_ISL_1068272	EPI_ISL_2777499	EPI_ISL_2777563	EPI_ISL_2777290
EPI_ISL_3050565	EPI_ISL_1068273	EPI_ISL_2777237	EPI_ISL_2777565	EPI_ISL_2777250
EPI_ISL_3050558	EPI_ISL_1068274	EPI_ISL_2777302	EPI_ISL_2777285	EPI_ISL_2777251
EPI_ISL_3050501	EPI_ISL_1068275	EPI_ISL_2777304	EPI_ISL_2777566	EPI_ISL_2777252
EPI_ISL_3050624	EPI_ISL_1068276	EPI_ISL_2777501	EPI_ISL_2777567	EPI_ISL_2777253
EPI_ISL_3050387	EPI_ISL_1068278	EPI_ISL_2777502	EPI_ISL_2777569	EPI_ISL_2777320
EPI_ISL_2777994	EPI_ISL_2777455	EPI_ISL_2777503	EPI_ISL_2777570	EPI_ISL_2777696
EPI_ISL_2777922	EPI_ISL_2777456	EPI_ISL_2777993	EPI_ISL_2777571	EPI_ISL_2777254
EPI_ISL_1068268	EPI_ISL_2777457	EPI_ISL_2777504	EPI_ISL_2777572	EPI_ISL_2777255
EPI_ISL_1068273	EPI_ISL_2777457	EPI_ISL_2777505	EPI_ISL_2777573	EPI_ISL_2777256
EPI_ISL_2777396	EPI_ISL_2777458	EPI_ISL_2102018	EPI_ISL_2777574	EPI_ISL_2777697
EPI_ISL_2777397	EPI_ISL_2777458	EPI_ISL_2777306	EPI_ISL_2777575	EPI_ISL_2777698
EPI_ISL_2777398	EPI_ISL_2777459	EPI_ISL_2777506	EPI_ISL_2777576	EPI_ISL_2777699
EPI_ISL_2777399	EPI_ISL_2777459	EPI_ISL_2777309	EPI_ISL_2777581	EPI_ISL_2777257
EPI_ISL_2777401	EPI_ISL_2777469	EPI_ISL_2777986	EPI_ISL_2777584	EPI_ISL_2777700
EPI_ISL_2777402	EPI_ISL_2777469	EPI_ISL_2777987	EPI_ISL_2777585	EPI_ISL_2777258
EPI_ISL_2777403	EPI_ISL_2777470	EPI_ISL_2777507	EPI_ISL_2777586	EPI_ISL_2777259
EPI_ISL_2777404	EPI_ISL_2777471	EPI_ISL_2777988	EPI_ISL_2777588	EPI_ISL_2777260
EPI_ISL_2777405	EPI_ISL_2777472	EPI_ISL_2777313	EPI_ISL_2777589	EPI_ISL_2777701
EPI_ISL_2777406	EPI_ISL_2777473	EPI_ISL_2777508	EPI_ISL_2777593	EPI_ISL_2777292

EPI_ISL_2777702
EPI_ISL_2777703
EPI_ISL_2777261
EPI_ISL_2777262
EPI_ISL_2777263
EPI_ISL_2777264
EPI_ISL_2777705
EPI_ISL_2777321
EPI_ISL_2777706
EPI_ISL_2777265
EPI_ISL_2777266
EPI_ISL_2777267
EPI_ISL_2777707
EPI_ISL_2777708
EPI_ISL_2777268
EPI_ISL_2777709
EPI_ISL_2777269
EPI_ISL_2777270
EPI_ISL_2777710
EPI_ISL_2777324
EPI_ISL_2777711
EPI_ISL_2777271
EPI_ISL_2777272
EPI_ISL_2777273
EPI_ISL_2777274
EPI_ISL_2777275
EPI_ISL_2777328
EPI_ISL_2777713
EPI_ISL_2777714
EPI_ISL_2777715
EPI_ISL_2777276
EPI_ISL_2777277
EPI_ISL_2777278
EPI_ISL_2777716
EPI_ISL_2777717
EPI_ISL_2777279
EPI_ISL_2777280
EPI_ISL_2777281
EPI_ISL_2777718
EPI_ISL_2777719
EPI_ISL_2777282
EPI_ISL_2777283
EPI_ISL_2777720
EPI_ISL_2777721
EPI_ISL_2777722
EPI_ISL_2777723
EPI_ISL_2777330
EPI_ISL_2777331
EPI_ISL_2777333
EPI_ISL_2777724
EPI_ISL_2777725
EPI_ISL_2777726
EPI_ISL_2777727
EPI_ISL_2777728
EPI_ISL_2777729
EPI_ISL_2777334
EPI_ISL_2777730
EPI_ISL_2777731
EPI_ISL_2777732
EPI_ISL_2777733
EPI_ISL_2777734
EPI_ISL_2777735
EPI_ISL_2777736
EPI_ISL_2777737
EPI_ISL_2777738
EPI_ISL_2777739
EPI_ISL_2777740
EPI_ISL_2777742
EPI_ISL_2777743
EPI_ISL_2777744
EPI_ISL_2777745
EPI_ISL_2777746
EPI_ISL_2777747
EPI_ISL_2777748
EPI_ISL_2777749
EPI_ISL_2777750
EPI_ISL_2777751
EPI_ISL_2777752
EPI_ISL_2777754
EPI_ISL_2777755
EPI_ISL_2777757
EPI_ISL_2777758
EPI_ISL_2777759
EPI_ISL_2777762
EPI_ISL_2777763
EPI_ISL_2777764
EPI_ISL_2777766
EPI_ISL_2777767
EPI_ISL_2777768
EPI_ISL_2777340
EPI_ISL_2777769
EPI_ISL_2777771
EPI_ISL_2777772
EPI_ISL_2777775
EPI_ISL_2777776
EPI_ISL_2777779
EPI_ISL_2777780
EPI_ISL_2777781
EPI_ISL_2777782
EPI_ISL_2777783
EPI_ISL_2777784
EPI_ISL_2777785
EPI_ISL_2777786
EPI_ISL_2777787
EPI_ISL_2777788
EPI_ISL_2777789
EPI_ISL_2777790
EPI_ISL_2777791
EPI_ISL_2777792
EPI_ISL_2777793
EPI_ISL_2777794
EPI_ISL_2777795
EPI_ISL_2777796
EPI_ISL_2777797
EPI_ISL_2102063
EPI_ISL_2777798
EPI_ISL_2777247
EPI_ISL_2777799
EPI_ISL_2777800
EPI_ISL_2777801
EPI_ISL_2777802
EPI_ISL_2777803
EPI_ISL_2777804
EPI_ISL_2777805
EPI_ISL_2777806
EPI_ISL_2777807
EPI_ISL_2777808
EPI_ISL_2777811
EPI_ISL_2777812
EPI_ISL_2777815
EPI_ISL_2777816
EPI_ISL_2777817
EPI_ISL_2777818
EPI_ISL_2777819
EPI_ISL_2777820
EPI_ISL_2777821
EPI_ISL_2777823
EPI_ISL_2777827
EPI_ISL_2777828
EPI_ISL_2777829
EPI_ISL_2777830
EPI_ISL_2777833
EPI_ISL_2777834
EPI_ISL_2777837
EPI_ISL_2777838
EPI_ISL_2777839
EPI_ISL_2777840
EPI_ISL_2777841
EPI_ISL_2777842
EPI_ISL_2777843
EPI_ISL_2777844
EPI_ISL_2777845
EPI_ISL_2777847
EPI_ISL_2777848
EPI_ISL_2777849
EPI_ISL_2777850
EPI_ISL_2777855
EPI_ISL_2777857
EPI_ISL_2777858
EPI_ISL_2777859
EPI_ISL_2777861
EPI_ISL_2777862
EPI_ISL_2777863
EPI_ISL_2777864
EPI_ISL_2777867
EPI_ISL_2777868
EPI_ISL_2777293
EPI_ISL_2777870
EPI_ISL_2777871
EPI_ISL_2777872
EPI_ISL_2777873
EPI_ISL_2777235
EPI_ISL_2777874
EPI_ISL_2777876
EPI_ISL_2777877
EPI_ISL_2777878
EPI_ISL_2777879
EPI_ISL_2777880
EPI_ISL_2777881
EPI_ISL_2777882
EPI_ISL_2777883
EPI_ISL_2777884
EPI_ISL_2777885
EPI_ISL_2777886
EPI_ISL_2777888
EPI_ISL_2777889
EPI_ISL_2777894
EPI_ISL_2777896
EPI_ISL_2777897
EPI_ISL_2777899
EPI_ISL_2777900
EPI_ISL_2777284
EPI_ISL_2777903
EPI_ISL_2777911
EPI_ISL_2777913
EPI_ISL_2777915
EPI_ISL_2777916
EPI_ISL_2777917
EPI_ISL_2777919
EPI_ISL_2777923
EPI_ISL_2777932
EPI_ISL_2777935
EPI_ISL_2777936
EPI_ISL_2777938
EPI_ISL_2777939
EPI_ISL_2777940
EPI_ISL_2777948
EPI_ISL_2777950
EPI_ISL_2777295
EPI_ISL_2777345
EPI_ISL_2777952
EPI_ISL_2777954
EPI_ISL_2777955
EPI_ISL_2777962
EPI_ISL_2777964
EPI_ISL_2777965
EPI_ISL_2777243
EPI_ISL_2777244
EPI_ISL_2777245
EPI_ISL_2777297
EPI_ISL_2777318
EPI_ISL_2777346
EPI_ISL_2777347
EPI_ISL_2777348
EPI_ISL_4030383
EPI_ISL_4030319
EPI_ISL_3050307
EPI_ISL_3050308
EPI_ISL_3050502
EPI_ISL_3050503
EPI_ISL_3050337
EPI_ISL_3050344
EPI_ISL_3050401
EPI_ISL_3050519
EPI_ISL_3050434
EPI_ISL_3050315
EPI_ISL_3050354
EPI_ISL_3050403
EPI_ISL_3050506
EPI_ISL_4030320
EPI_ISL_3050636
EPI_ISL_4030318
EPI_ISL_4030305
EPI_ISL_3050566
EPI_ISL_3050302
EPI_ISL_3050303
EPI_ISL_3050332
EPI_ISL_3050464
EPI_ISL_3050309
EPI_ISL_4030306
EPI_ISL_3050593
EPI_ISL_3050319
EPI_ISL_3050406
EPI_ISL_3050605
EPI_ISL_4030321
EPI_ISL_3050490
EPI_ISL_4030322
EPI_ISL_3050407
EPI_ISL_4030323
EPI_ISL_3050499
EPI_ISL_3050312
EPI_ISL_3050367
EPI_ISL_3050470
EPI_ISL_3050471
EPI_ISL_3050627
EPI_ISL_3050538
EPI_ISL_3050570
EPI_ISL_3050384
EPI_ISL_3050391
EPI_ISL_3050394
EPI_ISL_3050436
EPI_ISL_4030307
EPI_ISL_4030312
EPI_ISL_3050298
EPI_ISL_3050375
EPI_ISL_3050376
EPI_ISL_4030313
EPI_ISL_4030311
EPI_ISL_3050322
EPI_ISL_3050551
EPI_ISL_3050381
EPI_ISL_3050415
EPI_ISL_3050431
EPI_ISL_3050529
EPI_ISL_4030368
EPI_ISL_4030369
EPI_ISL_4030370
EPI_ISL_4030371
EPI_ISL_4030372
EPI_ISL_4030373
EPI_ISL_4030374
EPI_ISL_4030375
EPI_ISL_4030376
EPI_ISL_4030377
EPI_ISL_4030378
EPI_ISL_4030379
EPI_ISL_4030380
EPI_ISL_4030381
EPI_ISL_4030382
EPI_ISL_4030324
EPI_ISL_4030325
EPI_ISL_4030326
EPI_ISL_4030314
EPI_ISL_4030327
EPI_ISL_4030328
EPI_ISL_4030310
EPI_ISL_4030329
EPI_ISL_4030330
EPI_ISL_4030331
EPI_ISL_4030332
EPI_ISL_4030333
EPI_ISL_4030334
EPI_ISL_4030335
EPI_ISL_4030336
EPI_ISL_4030337
EPI_ISL_4030338
EPI_ISL_4030339
EPI_ISL_4030340
EPI_ISL_4030341
EPI_ISL_4030342
EPI_ISL_4030343
EPI_ISL_4030344
EPI_ISL_4030345
EPI_ISL_4030346
EPI_ISL_4030347
EPI_ISL_4030348
EPI_ISL_4030349
EPI_ISL_4030350
EPI_ISL_4030351
EPI_ISL_4030352
EPI_ISL_4030353
EPI_ISL_4030354
EPI_ISL_4030315
EPI_ISL_4030355
EPI_ISL_4030317
EPI_ISL_4030308
EPI_ISL_4030309
EPI_ISL_4030356
EPI_ISL_4030316
EPI_ISL_4030357
EPI_ISL_4030358
EPI_ISL_4030359
EPI_ISL_4030360
EPI_ISL_4030361
EPI_ISL_4030362
EPI_ISL_4030363
EPI_ISL_4030364
EPI_ISL_4030365
EPI_ISL_4030366
EPI_ISL_4030367
EPI_ISL_1261123
EPI_ISL_2777969
EPI_ISL_2777970
EPI_ISL_2777971
EPI_ISL_2777972
EPI_ISL_2777523
EPI_ISL_2777531
EPI_ISL_2777548
EPI_ISL_2777973
EPI_ISL_2777974
EPI_ISL_2777975
EPI_ISL_2777976
EPI_ISL_2777568
EPI_ISL_2777578
EPI_ISL_2777579
EPI_ISL_2777287
EPI_ISL_2777582
EPI_ISL_2777583
EPI_ISL_2777587
EPI_ISL_2777590
EPI_ISL_2777597
EPI_ISL_2777602
EPI_ISL_2777608
EPI_ISL_2777611
EPI_ISL_2777977
EPI_ISL_2777614
EPI_ISL_2777978
EPI_ISL_2777617
EPI_ISL_2777625
EPI_ISL_2777979

EPI_ISL_2777981	EPI_ISL_3050351	EPI_ISL_3050599	EPI_ISL_3050528	EPI_ISL_2777846
EPI_ISL_2777982	EPI_ISL_3050530	EPI_ISL_3050342	EPI_ISL_3050380	EPI_ISL_2777853
EPI_ISL_2777633	EPI_ISL_3050531	EPI_ISL_3050395	EPI_ISL_3050438	EPI_ISL_2777856
EPI_ISL_2777983	EPI_ISL_3050352	EPI_ISL_3050606	EPI_ISL_3050361	EPI_ISL_2777860
EPI_ISL_2777984	EPI_ISL_3050323	EPI_ISL_3050607	EPI_ISL_3050362	EPI_ISL_2777343
EPI_ISL_2777640	EPI_ISL_3050432	EPI_ISL_3050608	EPI_ISL_3050440	EPI_ISL_2777869
EPI_ISL_2777649	EPI_ISL_3050575	EPI_ISL_3050609	EPI_ISL_3050452	EPI_ISL_2777890
EPI_ISL_2777652	EPI_ISL_3050518	EPI_ISL_3050423	EPI_ISL_3050486	EPI_ISL_2777893
EPI_ISL_2777656	EPI_ISL_3050642	EPI_ISL_3050396	EPI_ISL_3050487	EPI_ISL_2777895
EPI_ISL_2777660	EPI_ISL_3050297	EPI_ISL_3050397	EPI_ISL_3050645	EPI_ISL_2777901
EPI_ISL_2777661	EPI_ISL_3050353	EPI_ISL_3050491	EPI_ISL_3050646	EPI_ISL_2777902
EPI_ISL_2777929	EPI_ISL_3050497	EPI_ISL_3050390	EPI_ISL_3050453	EPI_ISL_2777906
EPI_ISL_2777663	EPI_ISL_3050505	EPI_ISL_3050408	EPI_ISL_3050454	EPI_ISL_2777907
EPI_ISL_2777666	EPI_ISL_3050584	EPI_ISL_3050424	EPI_ISL_3050455	EPI_ISL_2777344
EPI_ISL_2777667	EPI_ISL_3050416	EPI_ISL_3050533	EPI_ISL_3050456	EPI_ISL_2777912
EPI_ISL_2777671	EPI_ISL_3050418	EPI_ISL_3050469	EPI_ISL_3050495	EPI_ISL_2777918
EPI_ISL_2777673	EPI_ISL_3050402	EPI_ISL_3050498	EPI_ISL_3050496	EPI_ISL_2777920
EPI_ISL_2777674	EPI_ISL_3050545	EPI_ISL_3050493	EPI_ISL_3050632	EPI_ISL_2777921
EPI_ISL_2777679	EPI_ISL_3050638	EPI_ISL_3050494	EPI_ISL_3050633	EPI_ISL_2777926
EPI_ISL_2777316	EPI_ISL_3050521	EPI_ISL_3050536	EPI_ISL_3050634	EPI_ISL_2777927
EPI_ISL_2777680	EPI_ISL_3050441	EPI_ISL_3050537	EPI_ISL_3050649	EPI_ISL_2777930
EPI_ISL_2777681	EPI_ISL_3050592	EPI_ISL_3050523	EPI_ISL_3050514	EPI_ISL_2777933
EPI_ISL_2777682	EPI_ISL_3050442	EPI_ISL_3050524	EPI_ISL_3050543	EPI_ISL_2777945
EPI_ISL_2777685	EPI_ISL_3050435	EPI_ISL_3050525	EPI_ISL_3050544	EPI_ISL_2777949
EPI_ISL_2777695	EPI_ISL_3050554	EPI_ISL_3050509	EPI_ISL_3050552	EPI_ISL_2777951
EPI_ISL_2777359	EPI_ISL_3050459	EPI_ISL_3050527	EPI_ISL_2778008	EPI_ISL_2777953
EPI_ISL_2777332	EPI_ISL_3050460	EPI_ISL_3050328	EPI_ISL_2778009	EPI_ISL_2777957
EPI_ISL_2777753	EPI_ISL_3050461	EPI_ISL_3050588	EPI_ISL_3050386	EPI_ISL_2777960
EPI_ISL_2777756	EPI_ISL_3050580	EPI_ISL_3050589	EPI_ISL_3050546	EPI_ISL_2777288
EPI_ISL_2777760	EPI_ISL_3050581	EPI_ISL_3050590	EPI_ISL_2777442	EPI_ISL_2777349
EPI_ISL_2777773	EPI_ISL_3050316	EPI_ISL_3050383	EPI_ISL_2777447	EPI_ISL_2777350
EPI_ISL_2777774	EPI_ISL_3050547	EPI_ISL_3050569	EPI_ISL_2777514	EPI_ISL_2777351
EPI_ISL_2777778	EPI_ISL_3050317	EPI_ISL_3050600	EPI_ISL_2777529	EPI_ISL_2777352
EPI_ISL_2777810	EPI_ISL_3050331	EPI_ISL_3050602	EPI_ISL_2777550	EPI_ISL_2777353
EPI_ISL_2777813	EPI_ISL_3050557	EPI_ISL_3050472	EPI_ISL_2777555	EPI_ISL_2777354
EPI_ISL_2777814	EPI_ISL_3050559	EPI_ISL_3050611	EPI_ISL_2777577	EPI_ISL_2777355
EPI_ISL_2777824	EPI_ISL_3050560	EPI_ISL_3050500	EPI_ISL_2777580	EPI_ISL_2777968
EPI_ISL_2777825	EPI_ISL_3050356	EPI_ISL_3050612	EPI_ISL_2777592	EPI_ISL_3050616
EPI_ISL_2777852	EPI_ISL_3050346	EPI_ISL_3050603	EPI_ISL_2777594	EPI_ISL_3050574
EPI_ISL_2777854	EPI_ISL_3050329	EPI_ISL_3050625	EPI_ISL_2777596	EPI_ISL_3050458
EPI_ISL_2777865	EPI_ISL_3050365	EPI_ISL_3050626	EPI_ISL_2777598	EPI_ISL_3050488
EPI_ISL_2777866	EPI_ISL_3050388	EPI_ISL_3050628	EPI_ISL_2777607	EPI_ISL_3050504
EPI_ISL_2777887	EPI_ISL_3050358	EPI_ISL_3050629	EPI_ISL_2777610	EPI_ISL_3050572
EPI_ISL_2777891	EPI_ISL_3050404	EPI_ISL_3050637	EPI_ISL_2777613	EPI_ISL_3050573
EPI_ISL_2777904	EPI_ISL_3050338	EPI_ISL_3050571	EPI_ISL_2777619	EPI_ISL_3050576
EPI_ISL_2777905	EPI_ISL_3050420	EPI_ISL_3050613	EPI_ISL_2777623	EPI_ISL_3050577
EPI_ISL_2777908	EPI_ISL_3050586	EPI_ISL_3050296	EPI_ISL_2777629	EPI_ISL_3050578
EPI_ISL_2777909	EPI_ISL_3050587	EPI_ISL_3050614	EPI_ISL_2777644	EPI_ISL_3050579
EPI_ISL_2777910	EPI_ISL_3050359	EPI_ISL_3050368	EPI_ISL_2777648	EPI_ISL_3050553
EPI_ISL_2777914	EPI_ISL_3050489	EPI_ISL_3050369	EPI_ISL_2777653	EPI_ISL_3050382
EPI_ISL_2777924	EPI_ISL_3050462	EPI_ISL_3050370	EPI_ISL_2777664	EPI_ISL_3050364
EPI_ISL_2777925	EPI_ISL_3050463	EPI_ISL_3050371	EPI_ISL_2777665	EPI_ISL_3050433
EPI_ISL_2777928	EPI_ISL_3050467	EPI_ISL_3050385	EPI_ISL_2777672	EPI_ISL_3050417
EPI_ISL_2777931	EPI_ISL_3050318	EPI_ISL_3050392	EPI_ISL_2777677	EPI_ISL_3050520
EPI_ISL_2777941	EPI_ISL_3050334	EPI_ISL_3050640	EPI_ISL_2777684	EPI_ISL_3050443
EPI_ISL_2777942	EPI_ISL_3050325	EPI_ISL_3050393	EPI_ISL_2777688	EPI_ISL_3050355
EPI_ISL_2777943	EPI_ISL_3050561	EPI_ISL_3050474	EPI_ISL_2777694	EPI_ISL_3050555
EPI_ISL_2777944	EPI_ISL_3050582	EPI_ISL_3050475	EPI_ISL_2777712	EPI_ISL_3050585
EPI_ISL_2777985	EPI_ISL_3050421	EPI_ISL_3050510	EPI_ISL_2777329	EPI_ISL_3050507
EPI_ISL_2777946	EPI_ISL_3050619	EPI_ISL_3050511	EPI_ISL_2777335	EPI_ISL_3050556
EPI_ISL_2777956	EPI_ISL_3050620	EPI_ISL_3050630	EPI_ISL_2777336	EPI_ISL_3050567
EPI_ISL_2777958	EPI_ISL_3050300	EPI_ISL_3050631	EPI_ISL_2777337	EPI_ISL_3050532
EPI_ISL_2777959	EPI_ISL_3050621	EPI_ISL_3050409	EPI_ISL_2777338	EPI_ISL_3050548
EPI_ISL_2777991	EPI_ISL_3050622	EPI_ISL_3050540	EPI_ISL_2777339	EPI_ISL_3050324
EPI_ISL_2777961	EPI_ISL_3050604	EPI_ISL_3050349	EPI_ISL_2777761	EPI_ISL_3050549
EPI_ISL_2777966	EPI_ISL_3050639	EPI_ISL_3050379	EPI_ISL_2777770	EPI_ISL_3050345
EPI_ISL_2777967	EPI_ISL_3050305	EPI_ISL_3050437	EPI_ISL_2777341	EPI_ISL_3050405
EPI_ISL_3050457	EPI_ISL_3050326	EPI_ISL_3050478	EPI_ISL_2777342	EPI_ISL_3050419
EPI_ISL_3050515	EPI_ISL_3050311	EPI_ISL_3050480	EPI_ISL_2777777	EPI_ISL_3050295
EPI_ISL_3050591	EPI_ISL_3050327	EPI_ISL_3050481	EPI_ISL_2777809	EPI_ISL_3050304
EPI_ISL_3050641	EPI_ISL_3050594	EPI_ISL_3050482	EPI_ISL_2777822	EPI_ISL_3050465
EPI_ISL_3050615	EPI_ISL_3050595	EPI_ISL_3050483	EPI_ISL_2777826	EPI_ISL_3050466
EPI_ISL_3050516	EPI_ISL_3050522	EPI_ISL_3050484	EPI_ISL_2777831	EPI_ISL_3050508
EPI_ISL_3050517	EPI_ISL_3050468	EPI_ISL_3050485	EPI_ISL_2777832	EPI_ISL_3050299
EPI_ISL_3050617	EPI_ISL_3050341	EPI_ISL_3050643	EPI_ISL_2777835	EPI_ISL_3050339
EPI_ISL_3050618	EPI_ISL_3050597	EPI_ISL_3050644	EPI_ISL_2777836	EPI_ISL_3050310

EPI_ISL_3050340
EPI_ISL_3050306
EPI_ISL_3050333
EPI_ISL_3050596
EPI_ISL_3050320
EPI_ISL_3050598
EPI_ISL_3050366
EPI_ISL_3050389
EPI_ISL_3050347
EPI_ISL_3050610
EPI_ISL_3050422
EPI_ISL_3050492
EPI_ISL_3050534
EPI_ISL_3050535
EPI_ISL_3050526
EPI_ISL_3050550
EPI_ISL_3050568
EPI_ISL_3050623
EPI_ISL_3050473
EPI_ISL_3050539
EPI_ISL_3050348
EPI_ISL_3050372
EPI_ISL_3050313
EPI_ISL_3050321
EPI_ISL_3050343
EPI_ISL_3050330
EPI_ISL_3050335
EPI_ISL_3050336
EPI_ISL_3050360
EPI_ISL_3050373
EPI_ISL_3050374
EPI_ISL_3050377
EPI_ISL_3050378
EPI_ISL_3050398
EPI_ISL_3050399
EPI_ISL_3050400
EPI_ISL_3050410
EPI_ISL_3050411
EPI_ISL_3050412
EPI_ISL_3050413
EPI_ISL_3050425
EPI_ISL_3050426
EPI_ISL_3050427
EPI_ISL_3050428
EPI_ISL_3050429
EPI_ISL_3050430
EPI_ISL_3050414
EPI_ISL_3050444
EPI_ISL_3050445
EPI_ISL_3050446
EPI_ISL_3050447
EPI_ISL_3050476
EPI_ISL_3050477
EPI_ISL_3050512
EPI_ISL_3050513
EPI_ISL_3050448
EPI_ISL_3050449
EPI_ISL_3050450
EPI_ISL_3050451
EPI_ISL_3050541
EPI_ISL_3050542
EPI_ISL_3050583
EPI_ISL_3050562
EPI_ISL_3050479
EPI_ISL_3050439
EPI_ISL_3050563
EPI_ISL_3050635
EPI_ISL_3050647
EPI_ISL_3050648
EPI_ISL_3050650
EPI_ISL_3050314
EPI_ISL_3050564
EPI_ISL_3050350

We gratefully acknowledge the following Authors from the Originating laboratories responsible for obtaining the specimens, as well as the Submitting laboratories where the genome data were generated and shared via GISAID, on which this research is based.

All Submitters of data may be contacted directly via www.gisaid.org

Authors are sorted alphabetically.

Accession ID	Originating Laboratory	Submitting Laboratory	Authors
EPI_ISL_2493266	AFIP SUDESTE	Instituto Butantan	Antonio Jorge Martins; Claudia Renata dos Santos Barros; David Schlesinger; Debora Botequio Moretti; Dimas Tadeu Covas; Elaine Cristina Marqueze; Elaine Vieira Santos; Evandra Strazza Rodrigues; Heidge Fukumasu; Jayme Augusto de Souza-Neto; José Salvatore Leister Patané; Luiz Alcantara; Luiz Lehmann Coutinho; Maria Carolina Elias; Maurício Lacerda Nogueira; Rafael dos Santos Bezerra; Raul Machado Neto; Rejane Maria Tommasini Grotto; Ricardo Haddad; Sandra Coccuzzo Sampaio Vessoni; Simone Kashima; Svetoslav Nanev Slavov; Vincent Louis Viala
EPI_ISL_2494097	AFIP SUL	Instituto Butantan	Antonio Jorge Martins; Claudia Renata dos Santos Barros; David Schlesinger; Debora Botequio Moretti; Dimas Tadeu Covas; Elaine Cristina Marqueze; Elaine Vieira Santos; Evandra Strazza Rodrigues; Heidge Fukumasu; Jayme Augusto de Souza-Neto; José Salvatore Leister Patané; Luiz Alcantara; Luiz Lehmann Coutinho; Maria Carolina Elias; Maurício Lacerda Nogueira; Rafael dos Santos Bezerra; Raul Machado Neto; Rejane Maria Tommasini Grotto; Ricardo Haddad; Sandra Coccuzzo Sampaio Vessoni; Simone Kashima; Svetoslav Nanev Slavov; Vincent Louis Viala
EPI_ISL_2378739	AMBULATORIO AVIACAO	Instituto Butantan	Antonio Jorge Martins; Claudia Renata dos Santos Barros; David Schlesinger; Debora Botequio Moretti; Dimas Tadeu Covas; Elaine Cristina Marqueze; Elaine Vieira Santos; Evandra Strazza Rodrigues; Heidge Fukumasu; Jayme Augusto de Souza-Neto; José Salvatore Leister Patané; Luiz Alcantara; Luiz Lehmann Coutinho; Maria Carolina Elias; Maurício Lacerda Nogueira; Rafael dos Santos Bezerra; Raul Machado Neto; Rejane Maria Tommasini Grotto; Ricardo Haddad; Sandra Coccuzzo Sampaio Vessoni; Simone Kashima; Svetoslav Nanev Slavov; Vincent Louis Viala
EPI_ISL_1625972	Ama J Angela	Instituto Adolfo Lutz, Interdiciplinary Procedures Center, Strategic Laboratory	Caio Vinicius Dias Lopes; Claudia Regina Gonçalves; Claudio Tavares Sacchi; Erica Valessa Ramos Gomes; Karoline Rodrigues Campos; Katia Correa de Oliveira Santos; Leonardo Jose Tadeu de Araujo
EPI_ISL_1365747	Associação Fundo de Incentivo a Pesquisa	Associação Fundo de Incentivo à Pesquisa (AFIP)	Debora Ribeiro Ramadan; Erika Rodrigues de Oliveira; Juliana Nogueira Martins Rodrigues; Priscila Farias Tempaku; Sergio Tufik; Soraya Sgambatti de Andrade
EPI_ISL_2493268	BIOFAST CENTRO	Instituto Butantan	Antonio Jorge Martins; Claudia Renata dos Santos Barros; David Schlesinger; Debora Botequio Moretti; Dimas Tadeu Covas; Elaine Cristina Marqueze; Elaine Vieira Santos; Evandra Strazza Rodrigues; Heidge Fukumasu; Jayme Augusto de Souza-Neto; José Salvatore Leister Patané; Luiz Alcantara; Luiz Lehmann Coutinho; Maria Carolina Elias; Maurício Lacerda Nogueira; Rafael dos Santos Bezerra; Raul Machado Neto; Rejane Maria Tommasini Grotto; Ricardo Haddad; Sandra Coccuzzo Sampaio Vessoni; Simone Kashima; Svetoslav Nanev Slavov; Vincent Louis Viala
EPI_ISL_2698099	Biology, UFLA, Universidade Federal de Lavras	Biology, UFLA	Barcante, J.; Cherem, J.; Fernandes, G.; Luciano, P.; Melo, D.; Pylro, V.
EPI_ISL_1060888, EPI_ISL_1060890, EPI_ISL_1060898, EPI_ISL_1060899, EPI_ISL_1060911, EPI_ISL_1060912, EPI_ISL_1060913, EPI_ISL_1060915			
see above	CDL Laboratorio Santos e Vidal LTDA.	Instituto de Medicina Tropical de Sao Paulo	Brazil-UK Centre for Arbovirus Discovery Diagnosis Genomics and Epidemiology (CADDE) Genomic Network - Instituto de Medicina Tropical
EPI_ISL_2345857	CENTRO DE SAUDE DE NOVA GUATAPORANGA	Instituto Butantan / Mendelics	Antonio Jorge Martins; Claudia Renata dos Santos Barros; David Schlesinger; Debora Botequio Moretti; Dimas Tadeu Covas; Elaine Cristina Marqueze; Elaine Vieira Santos; Evandra Strazza Rodrigues; Heidge Fukumasu; Jayme Augusto de Souza-Neto; José Salvatore Leister Patané; Luiz Alcantara; Luiz Lehmann Coutinho; Maria Carolina Elias; Maurício Lacerda Nogueira; Rafael dos Santos Bezerra; Raul Machado Neto; Rejane Maria Tommasini Grotto; Ricardo Haddad; Sandra Coccuzzo Sampaio Vessoni; Simone Kashima; Svetoslav Nanev Slavov; Vincent Louis Viala
EPI_ISL_2445548, EPI_ISL_2445549, EPI_ISL_2445557	CENTRO DE SAUDE III NELCIDIO DA SILVEIRA BASTOS	Instituto Butantan	Antonio Jorge Martins; Claudia Renata dos Santos Barros; David Schlesinger; Debora Botequio Moretti; Dimas Tadeu Covas; Elaine Cristina Marqueze; Elaine Vieira Santos; Evandra Strazza Rodrigues; Heidge Fukumasu; Jayme Augusto de Souza-Neto; José Salvatore Leister Patané; Luiz Alcantara; Luiz Lehmann Coutinho; Maria Carolina Elias; Maurício Lacerda Nogueira; Rafael dos Santos Bezerra; Raul Machado Neto; Rejane Maria Tommasini Grotto; Ricardo Haddad; Sandra Coccuzzo Sampaio Vessoni; Simone Kashima; Svetoslav Nanev Slavov; Vincent Louis Viala
EPI_ISL_2209702	CENTRO DE SAUDE III NELCIDIO DA SILVEIRA BASTOS	Instituto Butantan / Mendelics	Antonio Jorge Martins; Claudia Renata dos Santos Barros; David Schlesinger; Debora Botequio Moretti; Dimas Tadeu Covas; Elaine Cristina Marqueze; Elaine Vieira Santos; Evandra Strazza Rodrigues; Heidge Fukumasu; Jayme Augusto de Souza-Neto; José Salvatore Leister Patané; Luiz Alcantara; Luiz Lehmann Coutinho; Maria Carolina Elias; Maurício Lacerda Nogueira; Rafael dos Santos Bezerra; Raul Machado Neto; Rejane Maria Tommasini Grotto; Ricardo Haddad; Sandra Coccuzzo Sampaio Vessoni; Simone Kashima; Svetoslav Nanev Slavov; Vincent Louis Viala
EPI_ISL_2378744, EPI_ISL_2378753	CS DE APARECIDA DOESTE	Instituto Butantan	Antonio Jorge Martins; Claudia Renata dos Santos Barros; David Schlesinger; Debora Botequio Moretti; Dimas Tadeu Covas; Elaine Cristina Marqueze; Elaine Vieira Santos; Evandra Strazza Rodrigues; Heidge Fukumasu; Jayme Augusto de Souza-Neto; José Salvatore Leister Patané; Luiz Alcantara; Luiz Lehmann Coutinho; Maria Carolina Elias; Maurício Lacerda Nogueira; Rafael dos Santos Bezerra; Raul Machado Neto; Rejane Maria Tommasini Grotto; Ricardo Haddad; Sandra Coccuzzo Sampaio Vessoni; Simone Kashima; Svetoslav Nanev Slavov; Vincent Louis Viala
EPI_ISL_2378743, EPI_ISL_2493829	CSII DR WASHINGTON LUIS M RODRIGUES DA SILVA PITANGUEIRAS	Instituto Butantan	Antonio Jorge Martins; Claudia Renata dos Santos Barros; David Schlesinger; Debora Botequio Moretti; Dimas Tadeu Covas; Elaine Cristina Marqueze; Elaine Vieira Santos; Evandra Strazza Rodrigues; Heidge Fukumasu; Jayme Augusto de Souza-Neto; José Salvatore Leister Patané; Luiz Alcantara; Luiz Lehmann Coutinho; Maria Carolina Elias; Maurício Lacerda Nogueira; Rafael dos Santos Bezerra; Raul Machado Neto; Rejane Maria Tommasini Grotto; Ricardo Haddad; Sandra Coccuzzo Sampaio Vessoni; Simone Kashima; Svetoslav Nanev Slavov; Vincent Louis Viala
EPI_ISL_2698015	CTvacinas	CTvacinas	A.P.; B.L.; Coelho; D.B.; Dorlass; Durigon; E.G.; E.L.; F.G.; Fernandes; Fiorini, A.; Fonseca; G.P.; H.P.; K.L.; L.M.; Lourenco; Magalhaes; Oliveira; Ometto, T.; Peixoto, R.; R.D.; Sato, H.; Scagion; Teixeira, S.; Telezynski; Thomazelli
EPI_ISL_1067729, EPI_ISL_1067733, EPI_ISL_1067734, EPI_ISL_1583694, EPI_ISL_3266109, EPI_ISL_3266112	Central Public Health Laboratory - LACEN - Bahia, Salvador, Brazil	Central Public Health Laboratory - LACEN - Bahia, Salvador, Brazil	Arabela Leal; Breno Dominguez; Felicidade Pereira; Jaqueline Gomes; Luciana Oliveira; Luiz Alcantara; Marcela Gómez; Marta Giovanetti; Patrícia Cajado; Stephane Tosta; Vagner Fonseca; Vanessa Nardy
EPI_ISL_1628371	Centro De Saude II Ibitinga	Instituto Adolfo Lutz, Interdiciplinary Procedures Center, Strategic Laboratory	Caio Vinicius Dias Lopes; Claudia Regina Gonçalves; Claudio Tavares Sacchi; Erica Valessa Ramos Gomes; Karoline Rodrigues Campos; Katia Correa de Oliveira Santos; Leonardo Jose Tadeu de Araujo
EPI_ISL_833167, EPI_ISL_833169, EPI_ISL_833170, EPI_ISL_833171, EPI_ISL_833172, EPI_ISL_833173, EPI_ISL_833174			
see above	DB Diagnosticos do Brasil	Instituto Adolfo Lutz, Interdiciplinary Procedures Center, Strategic Laboratory	Claudia Regina Gonçalves; Claudio Tavares Sacchi; Erica Valessa Ramos Gomes; Karoline Rodrigues Campos
EPI_ISL_1060876, EPI_ISL_1060900, EPI_ISL_1060902, EPI_ISL_1060904, EPI_ISL_1060914	DB Diagnosticos do Brasil	Instituto de Medicina Tropical de Sao Paulo	Brazil-UK Centre for Arbovirus Discovery Diagnosis Genomics and Epidemiology (CADDE) Genomic Network - Instituto de Medicina Tropical
EPI_ISL_804814, EPI_ISL_804819, EPI_ISL_804820, EPI_ISL_804821, EPI_ISL_804823	DB Diagnosticos do Brasil	Laboratório de Parasitologia Médica - Instituto de Medicina Tropical - Universidade de São Paulo	Andrew Rambaut; CADDE Genomic Network.; CDL; Camila A. Maia da Silva; Cecília da Cunha Camilo; DB; Darlan Candido; Erika Regina Manuli; Ester C. Sabino; Flavia Cristina Sales; HEMOAM; Ingra Morales Claro; Lucas A. Moyses Franco; Maria do Perpétuo Socorro Sampaio Carvalho; Myuki Alfaia Esashika Crispim; Nelson Abrahim Fraiji; Nelson Gaburo; Nick Loman; Nuno Faria; Oliver G. Pybus; Pamela dos Santos Andrade; Renato A. Santana; Thais de Moura Coletti
EPI_ISL_1219136	Gonçalo Moniz Institute, FIOCRUZ, Bahia	Laboratory of Respiratory Viruses and Measles, Oswaldo Cruz Institute, FIOCRUZ	Alice Sampaio Rocha; Ana Carolina Mendonca; Anna Carolina Paixao; Fernando Motta; Luciana Appolinario; Marilda Siqueira on behalf of the Fiocruz COVID-19 Genomic Surveillance Network; Paola Resende; Renata Serrano Lopes; Ricardo Khouri; Tiago Graf
EPI_ISL_2801309, EPI_ISL_3102362, EPI_ISL_3102371	HEMOCE CENTRO DE HEMATOLOGIA E HEMOTERAPIA DO CEARA	Analytical Competence Molecular Epidemiology Lab/ACME, Oswaldo Cruz Foundation, Ceara (FIOCRUZ CE)	Cleber Furtado Aksenen; Cleber Furtado Aksenen e Suzana Porto Almeida; Fabio Miyajima; Fernando Braga Stehling; Francisco Eder de Moura Lopes; Jamille Maria Mendes Bezerra; Joaquim César do Nascimento Sousa Junior; Pedro Miguel Carneiro Jeronimo; Suzana Porto Almeida e Lucas Delerino; Thais Ferreira de Oliveira; Thais de Oliveira Costa; Ticiane Cavalcante de Souza; Veridiana Pessoa Miyajima
EPI_ISL_3102212	HGCC HOSPITAL GERAL DR CESAR CALS	Analytical Competence Molecular Epidemiology Lab/ACME, Oswaldo Cruz Foundation, Ceara (FIOCRUZ CE)	Cleber Furtado Aksenen; Fabio Miyajima; Fernando Braga Stehling; Francisco Eder de Moura Lopes; Jamille Maria Mendes Bezerra; Joaquim César do Nascimento Sousa Junior; Pedro Miguel Carneiro Jeronimo; Suzana Porto Almeida e Lucas Delerino; Thais Ferreira de Oliveira; Thais de Oliveira Costa; Ticiane Cavalcante de Souza; Veridiana Pessoa Miyajima
EPI_ISL_2017283, EPI_ISL_2017337, EPI_ISL_2017383, EPI_ISL_2017397, EPI_ISL_2187881, EPI_ISL_2188009, EPI_ISL_2222908, EPI_ISL_2348613, EPI_ISL_2466453, EPI_ISL_2466463, EPI_ISL_2466466, EPI_ISL_2497448, EPI_ISL_2497454, EPI_ISL_2497456, EPI_ISL_2497459, EPI_ISL_2497466, EPI_ISL_2497468, EPI_ISL_2497471, EPI_ISL_2497472, EPI_ISL_2497473, EPI_ISL_2497474, EPI_ISL_2497475, EPI_ISL_2497476, EPI_ISL_2617597, EPI_ISL_2617598, EPI_ISL_2617599, EPI_ISL_2617600, EPI_ISL_2617601, EPI_ISL_2617602, EPI_ISL_2617603, EPI_ISL_2617604, EPI_ISL_2617605, EPI_ISL_2617606, EPI_ISL_2617607, EPI_ISL_2617608, EPI_ISL_2617611, EPI_ISL_2617613, EPI_ISL_2617614, EPI_ISL_2617615, EPI_ISL_2617616, EPI_ISL_2617617, EPI_ISL_2617618, EPI_ISL_2617619, EPI_ISL_2617620, EPI_ISL_2617621, EPI_ISL_2617622, EPI_ISL_2617623, EPI_ISL_2617624, EPI_ISL_2617625, EPI_ISL_2921534, EPI_ISL_2921536, EPI_ISL_2921537, EPI_ISL_2921546, EPI_ISL_2921548, EPI_ISL_2921549, EPI_ISL_2921551, EPI_ISL_2921553, EPI_ISL_2921558, EPI_ISL_2921561, EPI_ISL_2921562, EPI_ISL_2921563, EPI_ISL_2921575, EPI_ISL_2921579, EPI_ISL_2921581, EPI_ISL_2921582, EPI_ISL_2921585, EPI_ISL_2921593, EPI_ISL_2921596, EPI_ISL_2921600, EPI_ISL_2921601, EPI_ISL_2921607			
see above	HLAGYN - Laboratorio de Imunologia de Transplantes de Goias	HLAGYN - Laboratorio de Imunologia de Transplantes de Goias	Alessandro Leonardo Alvares Magalhaes; Daniel Ferreira de Sousa; Danielle de Paiva Rezende; Erika Lopes Rocha Batista; Fernando Antonio Vinhal dos Santos; Frederico Rodrigues Vinhal; Lucas Carlos Gomes Pereira; Paola Cristina Resende Silva; Raphael Bessa Parmigiane; Sabrina Sara Moreira Duarte
EPI_ISL_3102234, EPI_ISL_3102470	HM HOSPITAL DE MESSEJANA DR CARLOS ALBERTO STUDART GOMES	Analytical Competence	Cleber Furtado Aksenen; Fabio Miyajima; Fernando Braga Stehling; Francisco Eder de Moura Lopes; Jamille Maria Mendes Bezerra; Joaquim César do Nascimento Sousa Junior; Pedro Miguel Carneiro Jeronimo; Suzana Porto Almeida e Lucas Delerino; Thais Ferreira de Oliveira; Thais de Oliveira Costa; Ticiane Cavalcante de Souza; Veridiana Pessoa Miyajima

		Molecular Epidemiology Lab/ACME, Oswaldo Cruz Foundation, Ceara (FIOCRUZ CE)	
EPI_ISL_1966737	HOSP MUN JOSANIAS CASTANHA BRAGA	Instituto Butantan	Antonio Jorge Martins; Bianca Cechetto Carlos. Mendelics: Bibiana Santos; Claudia Renata dos Santos Barros; Cíntia Bittar; David Schlesinger. Hemocentro Ribeirão Preto: Simone Kashima; Debora Botequio Moretti; Elaine Cristina Marqueze; Elaine Vieira dos Santos; Elisangela Chicaroni Mattos; Erika Freitas; Evandra Strazza Rodrigues; Felipe Allan da Silva da Costa; Flavia Aburjaile; Fábio Sossai Possebon; Guilherme Campos; Guilherme Targino Valente; Heidge Fukumasu. USP-Botucatu: Rejane Maria Tommasini Grotto; Helena Lage Ferreira; Instituto Butantan: Dimas Tadeu Covas; Jardelina de Souza Todao Bernardino; Jayme A. Souza-Neto; Jessika Cristina Chagas Lesbon; Jorge A. Petroli Marchesi; José Salvatore Leister Patané; João Paulo Kitajima; João Pessoa Araújo Jr.; Leila Sabrina Ullmann; Loyze Paola Oliveira de Lima; Luiz Aurelio de Campos Crispin. Centro de Genômica Funcional da ESALQ: Luiz Lehmann Coutinho; Luiz Carlos Junior de Alcantara; Lívia Sacchetto; Maisa C. Pereira Parra; Maria Carolina Elias; Marta Giovanetti; Marília Moraes; Maurício Lacerda Nogueira. Prefeitura de Sao Paulo: Melissa Palmieri.; Patricia Akemi Assato; Paula Rahal; Paulo Inacio da Costa; Rafael dos Santos Bezerra; Raquel de Lello Rocha Campos Cassano. NGS Soluções Genômicas: Pilar Drummond Sampaio Corrêa Mariani. FZEA-USP Pirassununga: Mirele Daiana Poleti; Raul Machado Neto; Ricardo Augusto Brassaloti; Ricardo Haddad; Rodrigo Tocantins Calado. FAMERP-SJRP: Cecília Artico Banho; Sandra Coccuzzo Sampaio; Svetoslav Nanev Slavov; Vagner Fonseca; Vincent Louis Viala
EPI_ISL_3102224	HOSPITAL E MATERNIDADE DRA ZILDA ARNS NEUMANN	Analytical Competence Molecular Epidemiology Lab/ACME, Oswaldo Cruz Foundation, Ceara (FIOCRUZ CE)	Cleber Furtado Aksenen; Fabio Miyajima; Fernando Braga Stehling; Francisco Eder de Moura Lopes; Jamille Maria Mendes Bezerra; Joaquim César do Nascimento Sousa Junior; Pedro Miguel Carneiro Jeronimo; Suzana Porto Almeida e Lucas Delerino; Thais Ferreira de Oliveira; Thais de Oliveira Costa; Ticiane Cavalcante de Souza; Veridiana Pessoa Miyajima
EPI_ISL_2445384	HOSPITAL ESTADUAL DE MIRANDOPOLIS	Instituto Butantan	Antonio Jorge Martins; Claudia Renata dos Santos Barros; David Schlesinger; Debora Botequio Moretti; Dimas Tadeu Covas; Elaine Cristina Marqueze; Elaine Vieira Santos; Evandra Strazza Rodrigues; Heidge Fukumasu; Jayme Augusto de Souza-Neto; José Salvatore Leister Patané; Luiz Alcantara; Luiz Lehmann Coutinho; Maria Carolina Elias; Maurício Lacerda Nogueira; Rafael dos Santos Bezerra; Raul Machado Neto; Rejane Maria Tommasini Grotto; Ricardo Haddad; Sandra Coccuzzo Sampaio Vessoni; Simone Kashima; Svetoslav Nanev Slavov; Vincent Louis Viala
EPI_ISL_2801336, EPI_ISL_2801348, EPI_ISL_2801349, EPI_ISL_2801358, EPI_ISL_2801370	HOSPITAL MUNICIPAL NOSSA SENHORA DOS MILAGRES	Analytical Competence Molecular Epidemiology Lab/ACME, Oswaldo Cruz Foundation, Ceara (FIOCRUZ CE)	Cleber Furtado Aksenen e Suzana Porto Almeida; Fabio Miyajima; Fernando Braga Stehling; Francisco Eder de Moura Lopes; Jamille Maria Mendes Bezerra; Joaquim César do Nascimento Sousa Junior; Pedro Miguel Carneiro Jeronimo; Thais Ferreira de Oliveira; Thais de Oliveira Costa; Ticiane Cavalcante de Souza; Veridiana Pessoa Miyajima
EPI_ISL_3102326	HOSPITAL REGIONAL DO SERTAO CENTRAL	Analytical Competence Molecular Epidemiology Lab/ACME, Oswaldo Cruz Foundation, Ceara (FIOCRUZ CE)	Cleber Furtado Aksenen; Fabio Miyajima; Fernando Braga Stehling; Francisco Eder de Moura Lopes; Jamille Maria Mendes Bezerra; Joaquim César do Nascimento Sousa Junior; Pedro Miguel Carneiro Jeronimo; Suzana Porto Almeida e Lucas Delerino; Thais Ferreira de Oliveira; Thais de Oliveira Costa; Ticiane Cavalcante de Souza; Veridiana Pessoa Miyajima
EPI_ISL_3102250, EPI_ISL_3102251	HOSPITAL SAO JOSE DE DOENCAS INFECCIOSAS	Analytical Competence Molecular Epidemiology Lab/ACME, Oswaldo Cruz Foundation, Ceara (FIOCRUZ CE)	Cleber Furtado Aksenen; Fabio Miyajima; Fernando Braga Stehling; Francisco Eder de Moura Lopes; Jamille Maria Mendes Bezerra; Joaquim César do Nascimento Sousa Junior; Pedro Miguel Carneiro Jeronimo; Suzana Porto Almeida e Lucas Delerino; Thais Ferreira de Oliveira; Thais de Oliveira Costa; Ticiane Cavalcante de Souza; Veridiana Pessoa Miyajima
EPI_ISL_906080, EPI_ISL_906081	Hospital Beneficiencia Portuguesa	Instituto Adolfo Lutz, Interdiciplinary Procedures Center, Strategic Laboratory	Claudia Regina Gonçalves; Claudio Tavares Sacchi; Erica Valessa Ramos Gomes; Karoline Rodrigues Campos
EPI_ISL_940626, EPI_ISL_940627	Hospital Central Sao Caetano do Sul	Instituto Adolfo Lutz, Interdiciplinary Procedures Center, Strategic Laboratory	Claudia Regina Gonçalves; Claudio Tavares Sacchi; Erica Valessa Ramos Gomes; Karoline Rodrigues Campos
EPI_ISL_906075	Hospital Geral de Vila Penteado Dr Jose Pangella Sao Paulo	Instituto Adolfo Lutz, Interdiciplinary Procedures Center, Strategic Laboratory	Claudia Regina Gonçalves; Claudio Tavares Sacchi; Erica Valessa Ramos Gomes; Karoline Rodrigues Campos
EPI_ISL_1381070, EPI_ISL_1381071	Hospital Municipal Cidade Tiradentes Carmem Prudente	Instituto Adolfo Lutz, Interdiciplinary Procedures Center, Strategic Laboratory	Caio Vinicius Dias Lopes; Claudia Regina Gonçalves; Claudio Tavares Sacchi; Erica Valessa Ramos Gomes; Karoline Rodrigues Campos
EPI_ISL_940620, EPI_ISL_940623, EPI_ISL_940624	Hospital Sao Joaquim - Beneficiencia Portuguesa	Instituto Adolfo Lutz, Interdiciplinary Procedures Center, Strategic Laboratory	Claudia Regina Gonçalves; Claudio Tavares Sacchi; Erica Valessa Ramos Gomes; Karoline Rodrigues Campos
EPI_ISL_906076	Hospital Sao Luiz Sao Caetano	Instituto Adolfo Lutz, Interdiciplinary Procedures Center, Strategic Laboratory	Claudia Regina Gonçalves; Claudio Tavares Sacchi; Erica Valessa Ramos Gomes; Karoline Rodrigues Campos
EPI_ISL_2614559, EPI_ISL_2614560	IAL Presidente Prudente	Instituto Adolfo Lutz, Interdiciplinary Procedures Center, Strategic Laboratory	Caio Vinicius Dias Lopes; Claudia Regina Gonçalves; Claudio Tavares Sacchi; Erica Valessa Ramos Gomes; Karoline Rodrigues Campos; Leonardo Jose Tadeu de Araujo
EPI_ISL_2445049	INSIDE DIAGNÓSTICOS SUL PARELHEIROS	Instituto Butantan	Antonio Jorge Martins; Claudia Renata dos Santos Barros; David Schlesinger; Debora Botequio Moretti; Dimas Tadeu Covas; Elaine Cristina Marqueze; Elaine Vieira Santos; Evandra Strazza Rodrigues; Heidge Fukumasu; Jayme Augusto de Souza-Neto; José Salvatore Leister Patané; Luiz Alcantara; Luiz Lehmann Coutinho; Maria Carolina Elias; Maurício Lacerda Nogueira; Rafael dos Santos Bezerra; Raul Machado Neto; Rejane Maria Tommasini Grotto; Ricardo Haddad; Sandra Coccuzzo Sampaio Vessoni; Simone Kashima; Svetoslav Nanev Slavov; Vincent Louis Viala
EPI_ISL_2828650	Instituto Adolfo Lutz - Reginal de Sao José do Rio Preto	Instituto Adolfo Lutz, Interdiciplinary Procedures Center, Strategic Laboratory	Caio Vinicius Dias Lopes; Claudia Regina Gonçalves; Claudio Tavares Sacchi; Erica Valessa Ramos Gomes; Karoline Rodrigues Campos
EPI_ISL_906069	Instituto Adolfo Lutz - Regional de Campinas	Instituto Adolfo Lutz, Interdiciplinary Procedures Center, Strategic Laboratory	Claudia Regina Gonçalves; Claudio Tavares Sacchi; Erica Valessa Ramos Gomes; Karoline Rodrigues Campos
EPI_ISL_2003135, EPI_ISL_2003136, EPI_ISL_2003137, EPI_ISL_2828699	Instituto Adolfo Lutz - Regional de Santos	Instituto Adolfo Lutz, Interdiciplinary Procedures Center, Strategic Laboratory	Caio Vinicius Dias Lopes; Claudia Regina Gonçalves; Claudio Tavares Sacchi; Erica Valessa Ramos Gomes; Karoline Rodrigues Campos; Leonardo Jose Tadeu de Araujo
EPI_ISL_1628348, EPI_ISL_2756426, EPI_ISL_2756458, EPI_ISL_2756487, EPI_ISL_2919283	Instituto Adolfo Lutz Central	Instituto Adolfo Lutz, Interdiciplinary Procedures Center, Strategic Laboratory	Caio Vinicius Dias Lopes; Claudia Regina Gonçalves; Claudio Tavares Sacchi; Erica Valessa Ramos Gomes; Karoline Rodrigues Campos; Katia Correa de Oliveira Santos; Leonardo Jose Tadeu de Araujo
EPI_ISL_2759057	Instituto de Biologia Molecular do Paraná (LAC)	Instituto Carlos Chagas - Fiocruz	Alessandra De Melo Aguiar; Andreia Akemi Suzukawa; Andréa Rodrigues Ávila; Bruno Dallagiovanna; Dalila Zanette; Eduardo Balsanelli; Emanuel Maltempi de Souza; Fabio Passetti; Fabricio Klerynton Marchini; Fábio de Oliveira Pedrosa; Guilherme Becker; Helisson Faoro; Hellen Geremias dos Santos; Irina Nastassja Riediger; Letusa Albrecht; Lucas Blanes; Luis Gustavo Morello; Lysangela Ronalte Alves; Maria do Carmo Debur; Mauro de Medeiros Oliveira; Michelle Orane Schemberger; Paola Cristina Resende; Sheila Cristina Nardeli; Tiago Gräf; Valter Antônio de Baura
EPI_ISL_2378677, EPI_ISL_2843922, EPI_ISL_2843928, EPI_ISL_2843931, EPI_ISL_2843932, EPI_ISL_2843936, EPI_ISL_2843940, EPI_ISL_2843967, EPI_ISL_2844013, EPI_ISL_2844023, EPI_ISL_2844031, EPI_ISL_2844033, EPI_ISL_2844034, EPI_ISL_2844039, EPI_ISL_2844046, EPI_ISL_2844062, EPI_ISL_2844074, EPI_ISL_2844080, EPI_ISL_2844088, EPI_ISL_2844095, EPI_ISL_2844101, EPI_ISL_2844113, EPI_ISL_2844123, EPI_ISL_2844124, EPI_ISL_2844130, EPI_ISL_2844141, EPI_ISL_2844146, EPI_ISL_2844153, EPI_ISL_2844164, EPI_ISL_2844166, EPI_ISL_2844167, EPI_ISL_2844169, EPI_ISL_2844173, EPI_ISL_2844174, EPI_ISL_2844180, EPI_ISL_2844186, EPI_ISL_3332329			
see above	Instituto de Biotecnologia - UNESP-Botucatu-SP	Instituto de Biotecnologia - UNESP-Botucatu-SP	Cecília Artico Banho; Cíntia Bittar; Fábio Sossai Possebon; Guilherme Campos; Helena Lage Ferreira; Jorge A. Petroli Marchesi; João Pessoa Araújo Jr.; Leila Sabrina Ullmann; Lívia Sacchetto; Maisa C. Pereira Parra; Marília Moraes; Maurício L. Nogueira; Paula Rahal; Paulo Inacio da Costa
EPI_ISL_2894877, EPI_ISL_2894878, EPI_ISL_2894879, EPI_ISL_2894880, EPI_ISL_2894881	Instituto de Medicina Tropical de Sao Paulo	Instituto de Medicina Tropical de Sao Paulo	Brazil-UK Centre for Arbovirus Discovery Diagnosis Genomics and Epidemiology (CADDE) Genomic Network - Instituto de Medicina Tropical

EPI_ISL_2488805	LACEN - Laboratório Central de Saúde Pública do Amapá	Evandro Chagas Institute	A.M.; Barbagelata; E.C.; E.M.A.; Ferreira; J.A.; Junior; K.C.; L.C.; L.S.; M.C.; P.S.; Pinheiro; Santos; Silva; Sousa; Sousa Junior; W.D.C.; da Silva
EPI_ISL_918499, EPI_ISL_918500, EPI_ISL_918501, EPI_ISL_918502, EPI_ISL_918503, EPI_ISL_918504, EPI_ISL_918505, EPI_ISL_918506, EPI_ISL_918507, EPI_ISL_918508, EPI_ISL_918509, EPI_ISL_918510, EPI_ISL_918511, EPI_ISL_1261683, EPI_ISL_1261685, EPI_ISL_1261690, EPI_ISL_1261694			
see above	LACEN - Laboratório Central de Saúde Pública do Amazonas	Evandro Chagas Institute	A.M.; Barbagelata; E.C.; E.M.A.; Ferreira; J.A.; Junior; K.C.; L.C.; L.S.; M.C.; P.S.; Pinheiro; Santos; Silva; Sousa; Sousa Junior; W.D.C.; da Silva
EPI_ISL_943990	LACEN do Estado de Goias	Instituto Adolfo Lutz, Interdiciplinary Procedures Center, Strategic Laboratory	Claudia Regina Gonçalves; Claudio Tavares Sacchi; Erica Valessa Ramos Gomes; Karoline Rodrigues Campos
EPI_ISL_2919206, EPI_ISL_2919208, EPI_ISL_2919225	LACEN do Estado de Tocantins	Instituto Adolfo Lutz, Interdiciplinary Procedures Center, Strategic Laboratory	Caio Vinicius Dias Lopes; Claudia Regina Gonçalves; Claudio Tavares Sacchi; Erica Valessa Ramos Gomes; Karoline Rodrigues Campos
EPI_ISL_906071, EPI_ISL_940614, EPI_ISL_940615, EPI_ISL_940617, EPI_ISL_940618	LACEN-PI DR. Costa Alvarenga	Instituto Adolfo Lutz, Interdiciplinary Procedures Center, Strategic Laboratory	Claudia Regina Gonçalves; Claudio Tavares Sacchi; Erica Valessa Ramos Gomes; Karoline Rodrigues Campos
EPI_ISL_3046269, EPI_ISL_3046274, EPI_ISL_3046279, EPI_ISL_3046324, EPI_ISL_3046339	LACEN/PE	WallauLab on behalf of Fiocruz COVID-19 Genomic Surveillance Network	Alexandre Freitas da Silva; Cassia Docena; Constância Flávia Junqueira Ayres; Filipe Zimmer Dezordi; Gabriel Luz Wallau; Gustavo Barbosa de Lima; Lais Ceschini Machado; Lilian Caroliny Amorim Silva; Marcelo Henrique dos Santos Paiva; Matheus Filgueira Bezerra; Sinval Pinto Brandão Filho
EPI_ISL_3010763, EPI_ISL_3010767, EPI_ISL_3010782, EPI_ISL_3010810, EPI_ISL_3010869, EPI_ISL_3010894, EPI_ISL_3010899, EPI_ISL_3010913, EPI_ISL_3010928, EPI_ISL_3020990, EPI_ISL_3047724			
see above	LATE - Laboratório de Técnicas Especiais - Hospital Israelita Albert Einstein	LATE - Laboratório de Técnicas Especiais - Hospital Israelita Albert Einstein	Alexandre Hideaki Takara; Ana Paula Moreira Salles; Anelisie da Silva Santos; Deyvid Amgarten; Erick Gustavo Dorlass; Fernanda de Mello Malta; João Renato Rebello Pinho; Marcio Anunciacao Menezes; Pedro Henrique Sebe Rodrigues; Raquel Riyuzo
EPI_ISL_1213190, EPI_ISL_1213202, EPI_ISL_1213204	LBM/UFPB	Bioinformatics Laboratory / LNCC	Alessandra P Lamarca; Alexandra L Gerber; Ana Paula Melo Mariano; Ana Paula de C Guimarães; Ana Tereza R Vasconcelos; Angela Maria Guimarães Santos; Bianca Mendes Maciel; Danielle Angst Secco; Eduardo Sérgio Soares Sousa; Eloiza Helena Campana; Francisco Paulo Freire Neto; George Rego Albuquerque; Kátia Castanho Scortecci; Lucymara Fassarella Agnez Lima; Luiz G P de Almeida; Luís Cristóvão Porto; Otavio J. Brustolini; Paulo Ricardo Nascimento; Ronaldo da Silva Francisco Jr; Sandra Rocha Gadelha; Selma Maria Bezerra Jeronimo; Vinícius Pietta Perez
EPI_ISL_2983181, EPI_ISL_2983183, EPI_ISL_2983184, EPI_ISL_2983230	Laboratorio Central de Saude Publica do Estado de Goias (LACEN/GO)	Laboratory of Respiratory Viruses and Measles, Oswaldo Cruz Institute, FIOCRUZ	Agatha Cristinne Prudencio; Alice Sampaio Rocha; Ana Carolina Mendonca; Ana Flavia Mendonça; Anna Carolina Paixao; Carmen Helena Ramos; Cassiane Casanova; Elisa Cavalcante Pereira; Fernando Motta; Flavia Pereira Amorim da Silva; Ighor Leonardo Arantes Gomes; Luciana Appolinario; Luiz Augusto Pereira; Marilda Siqueira on behalf of the Fiocruz COVID-19 Genomic Surveillance Network; Paola Resende; Rafael Souza Guedes; Renata Serrano Lopes; Taina Venas; Vinicius Lemes da Silva
EPI_ISL_2196274	Laboratorio Central de Saude Publica do Estado de Minas Gerais (LACEN/MG)	Laboratory of Respiratory Viruses and Measles, Oswaldo Cruz Institute, FIOCRUZ	Alice Sampaio Rocha; Ana Carolina Mendonca; Andre Felipe Leal Bernardes; Anna Carolina Paixao; Elisa Cavalcante Pereira; Fernando Motta; Luciana Appolinario; Marilda Siqueira on behalf of the Fiocruz COVID-19 Genomic Surveillance Network; Paola Resende; Renata Serrano Lopes; Taina Venas
EPI_ISL_2645423, EPI_ISL_2645436, EPI_ISL_2863818, EPI_ISL_2863836, EPI_ISL_2863894	Laboratorio Central de Saude Publica do Estado do Para (LACEN/PA)	Laboratory of Respiratory Viruses and Measles, Oswaldo Cruz Institute, FIOCRUZ	Agatha Cristinne Prudencio Soares; Alice Sampaio Rocha; Ana Carolina Mendonca; Anna Carolina Paixao; Elisa Cavalcante Pereira; Fernando Motta; Ighor Leonardo Arantes Gomes; Luciana Appolinario; Marilda Siqueira on behalf of the Fiocruz COVID-19 Genomic Surveillance Network; Paola Resende; Renata Serrano Lopes; Taina Venas; Valnete Andrade
EPI_ISL_3982734, EPI_ISL_3982735	Laboratorio Antonello, Pelotas, Rio Grande do Sul	Hemocentro de Ribeirao Preto FMRP USP	Antonio Jorge Martins; Claudia Renata dos Santos Barros; David Schlesinger; Debora Botequio Moretti; Dimas Tadeu Covas; Elaine Cristina Marqueze; Elaine Vieira Santos; Evandra Strazza Rodrigues; Heidge Fukumasu; Jayme Augusto de Souza-Neto; José Salvatore Leister Patané; Luiz Alcantara; Luiz Lehmann Coutinho; Maria Carolina Elias; Maurício Lacerda Nogueira; Rafael dos Santos Bezerra; Raul Machado Neto; Rejane Maria Tommasini Grotto; Ricardo Haddad; Rodrigo Proto de Siqueira; Sandra Coccuzzo Sampaio Vessoni; Simone Kashima; Svetoslav Nanev Slavov; VV Cantarelli; Vincent Louis Viala
EPI_ISL_3982733	Laboratorio Antonello, Pelotas, Rio Grande do Sul	Hemocentro de Ribeirao Preto/FMRP-USP	Antonio Jorge Martins; Claudia Renata dos Santos Barros; David Schlesinger; Debora Botequio Moretti; Dimas Tadeu Covas; Elaine Cristina Marqueze; Elaine Vieira Santos; Evandra Strazza Rodrigues; Heidge Fukumasu; Jayme Augusto de Souza-Neto; José Salvatore Leister Patané; Luiz Alcantara; Luiz Lehmann Coutinho; Maria Carolina Elias; Maurício Lacerda Nogueira; Rafael dos Santos Bezerra; Raul Machado Neto; Rejane Maria Tommasini Grotto; Ricardo Haddad; Rodrigo Proto de Siqueira; Sandra Coccuzzo Sampaio Vessoni; Simone Kashima; Svetoslav Nanev Slavov; VV Cantarelli; Vincent Louis Viala
EPI_ISL_1858688, EPI_ISL_2101733, EPI_ISL_2534937, EPI_ISL_2691526, EPI_ISL_2837090, EPI_ISL_2837127, EPI_ISL_2837128, EPI_ISL_2837129, EPI_ISL_2837131, EPI_ISL_2837158			
see above	Laboratorio Central Noel Nutels	Bioinformatics Laboratory / LNCC	Alessandra P Lamarca; Alexandra L Gerber; Amilcar Tanuri; Ana Paula de C Guimaraes; Ana Paula de C Guimarães; Ana Tereza R Vasconcelos; Andrea Cony Cavalcanti; Andréa Cony Cavalcanti; Caio Luiz Pereira Ribeiro; Cassia Alves; Cintia Policarpo; Claudia Maria Braga de Mello; Cristiane Gomes da Silva; Diana Mariani; Douglas Terra Machado; Flavio Dias da Silva; Flávio Dias da Silva; Gleidson da Silva de Oliveira; Leandro Magalhaes de Souza; Leandro Magalhães de Souza; Liliane Cavalcante; Luiz G P de Almeida; Marcio Henrique de Oliveira Garcia; Mario Sergio Ribeiro; Ronaldo da Silva F Jr; Silvia Carvalho; Thais Felix Cruz
EPI_ISL_2274078, EPI_ISL_2274083	Laboratorio Central de Saude Publica do Estado Maranhao (LACEN-MA)	Laboratory of Respiratory Viruses and Measles, Oswaldo Cruz Institute, FIOCRUZ	Alice Sampaio Rocha; Ana Carolina Mendonca; Anna Carolina Paixao; Elisa Cavalcante Pereira; Fernando Motta; Lidio Gonçalves Lima Neto; Luciana Appolinario; Marilda Siqueira on behalf of the Fiocruz COVID-19 Genomic Surveillance Network; Paola Resende; Renata Serrano Lopes; Taina Venas
EPI_ISL_2536318, EPI_ISL_2536335	Laboratorio Central de Saude Publica do Estado da Paraiba (LACEN-PB)	Laboratory of Respiratory Viruses and Measles, Oswaldo Cruz Institute, FIOCRUZ	Alice Sampaio Rocha; Ana Carolina Mendonca; Anna Carolina Paixao; Dalane Loudal Florentino Teixeira; Elisa Cavalcante Pereira; Fernando Motta; Joao Felipe Bezerra; Luciana Appolinario; Marilda Siqueira on behalf of the Fiocruz COVID-19 Genomic Surveillance Network; Paola Resende; Renata Serrano Lopes; Taina Venas
EPI_ISL_2466238, EPI_ISL_2466239, EPI_ISL_2557313, EPI_ISL_2645393, EPI_ISL_2983071, EPI_ISL_2983075	Laboratorio Central de Saude Publica do Estado de Alagoas (LACEN/AL)	Laboratory of Respiratory Viruses and Measles, Oswaldo Cruz Institute, FIOCRUZ	Agatha Cristinne Prudencio; Alice Sampaio Rocha; Ana Carolina Mendonca; Anderson Brandao Leite; Anna Carolina Paixao; Elisa Cavalcante Pereira; Fernando Motta; Ighor Leonardo Arantes Gomes; Luciana Appolinario; Marilda Siqueira on behalf of the Fiocruz COVID-19 Genomic Surveillance Network; Paola Resende; Renata Serrano Lopes; Taina Venas
EPI_ISL_2983341, EPI_ISL_2983358	Laboratorio Central de Saude Publica do Estado de Santa Catarina (LACEN-SC)	Laboratory of Respiratory Viruses and Measles, Oswaldo Cruz Institute, FIOCRUZ	Alice Sampaio Rocha; Ana Carolina Mendonca; Anna Carolina Paixao; Darcita Buerger Rovaris; Elisa Cavalcante Pereira; Fernando Motta; Luciana Appolinario; Marilda Siqueira on behalf of the Fiocruz COVID-19 Genomic Surveillance Network; Paola Resende; Renata Serrano Lopes; Sandra Bianchini Fernandes; Taina Venas
EPI_ISL_2731506, EPI_ISL_2983390	Laboratorio Central de Saude Publica do Estado de Santa Catarina (LACEN/SC)	Laboratory of Respiratory Viruses and Measles, Oswaldo Cruz Institute, FIOCRUZ	Alice Sampaio Rocha; Ana Carolina Mendonca; Anna Carolina Paixao; Darcita Buerger Rovaris; Elisa Cavalcante Pereira; Fernando Motta; Luciana Appolinario; Marilda Siqueira on behalf of the Fiocruz COVID-19 Genomic Surveillance Network; Paola Resende; Renata Serrano Lopes; Sandra Bianchini Fernandes; Taina Venas
EPI_ISL_2157374, EPI_ISL_2157473, EPI_ISL_2157474, EPI_ISL_2157475, EPI_ISL_2157476, EPI_ISL_2157477, EPI_ISL_2157478, EPI_ISL_2157546, EPI_ISL_2157596			
see above	Laboratorio Central de Saude Publica do Estado de Sergipe (LACEN/SE)	Laboratory of Respiratory Viruses and Measles, Oswaldo Cruz Institute, FIOCRUZ	Alice Sampaio Rocha; Ana Carolina Mendonca; Anna Carolina Paixao; Cliomar Alves dos Santos; Elisa Cavalcante Pereira; Fernando Motta; Luciana Appolinario; Marilda Siqueira on behalf of the Fiocruz COVID-19 Genomic Surveillance Network; Paola Resende; Renata Serrano Lopes; Tainá Moreira Martins Venas
EPI_ISL_1219134	Laboratorio Central de Saude Publica do Estado do Alagoas (LACEN-AL)	Laboratory of Respiratory Viruses and Measles, Oswaldo Cruz Institute, FIOCRUZ	Alice Sampaio Rocha; Ana Carolina Mendonca; Anderson Brandao Leite; Anna Carolina Paixao; Fernando Motta; Luciana Appolinario; Marilda Siqueira on behalf of the Fiocruz COVID-19 Genomic Surveillance Network; Paola Resende; Renata Serrano Lopes
EPI_ISL_2645517, EPI_ISL_2645521, EPI_ISL_3045450, EPI_ISL_3045451, EPI_ISL_3045456, EPI_ISL_3061878	Laboratorio Central de Saude Publica do Estado do Espirito Santo (LACEN/ES)	Laboratory of Respiratory Viruses and Measles, Oswaldo Cruz Institute, FIOCRUZ	Alice Sampaio Rocha; Ana Carolina Mendonca; Anna Carolina Paixao; Eilisa Cavalcante Pereira; Elisa Cavalcante Pereira; Fernando Motta; Luciana Appolinario; Marilda Siqueira on behalf of the Fiocruz COVID-19 Genomic Surveillance Network; Paola Resende; Renata Serrano Lopes; Rodrigo Ribeiro Rodrigues; Taina Venas

EPI_ISL_2983188, EPI_ISL_2983189, EPI_ISL_2983190, EPI_ISL_2983192, EPI_ISL_2983193, EPI_ISL_2983194, EPI_ISL_2983239, EPI_ISL_2983240, EPI_ISL_2983253, EPI_ISL_2983271, EPI_ISL_2983277, EPI_ISL_2983278, EPI_ISL_2983290, EPI_ISL_2983299, EPI_ISL_2983302			
see above	Laboratorio Central de Saude Publica do Estado do Maranhao (LACEN-MA)	Laboratory of Respiratory Viruses and Measles, Oswaldo Cruz Institute, FIOCRUZ	Agatha Cristinne Prudencio; Alice Sampaio Rocha; Ana Carolina Mendonca; Anna Carolina Paixao; Elisa Cavalcante Pereira; Fernando Motta; Ighor Leonardo Arantes Gomes; Lidio Gonçalves Lima Neto; Luciana Appolinario; Marilda Siqueira on behalf of the Fiocruz COVID-19 Genomic Surveillance Network; Paola Resende; Renata Serrano Lopes; Taina Moreira Venas; Tainá Venas
EPI_ISL_1219133	Laboratorio Central de Saude Publica do Estado do Parana (LACEN-PR)	Laboratory of Respiratory Viruses and Measles, Oswaldo Cruz Institute, FIOCRUZ	Alice Sampaio Rocha; Ana Carolina Mendonca; Anna Carolina Paixao; Fernando Motta; Irina Nastassja Riediger; Luciana Appolinario; Maria do Carmo Debur; Marilda Siqueira on behalf of the Fiocruz COVID-19 Genomic Surveillance Network; Paola Resende; Renata Serrano Lopes
EPI_ISL_2661759, EPI_ISL_2661762, EPI_ISL_2661764, EPI_ISL_2661780, EPI_ISL_2982804	Laboratorio Central de Saude Publica do Estado do Rio Grande do Sul (LACEN-RS)	Laboratory of Respiratory Viruses and Measles, Oswaldo Cruz Institute, FIOCRUZ	Alice Sampaio Rocha; Ana Carolina Mendonca; Anderson Brandao Leite; Anna Carolina Paixao; Elisa Cavalcante Pereira; Fernando Motta; Luciana Appolinario; Marilda Siqueira on behalf of the Fiocruz COVID-19 Genomic Surveillance Network; Paola Resende; Renata Serrano Lopes; Richard Salvato; Taina Venas; Tatiana Schaffer Gregianini
EPI_ISL_3048782	Laboratorio Central de Saude Publica do Estado do Rio Grande do Sul (LACEN-RS)	Laboratório de Biologia Molecular da Universidade Federal de Ciências da Saúde de Porto Alegre	Adriana Seixas; Ana B. G. Veiga; Ana Paula Muterle Varela; Fabiana Quoos Mayer; Fernando Hayashi Sant'Anna; Janira Prichula; Letícia Garay Martins; Richard Steiner Salvato; Tatiana Schäffer Gregianini
EPI_ISL_2139504, EPI_ISL_2139510, EPI_ISL_2139516, EPI_ISL_2139531, EPI_ISL_2139540, EPI_ISL_2139545	Laboratorio Exame	Universidade Federal de Ciencias da Saude de Porto Alegre	Gabriel Dickin Caldana et al.; Vinícius Bonetti Franceschi
EPI_ISL_2777426	Laboratorio de Ecologia de Doencas Transmissiveis na Amazonia, Instituto Leonidas e Maria Deane - Fiocruz Amazonia	Laboratorio de Ecologia de Doencas Transmissiveis na Amazonia, Instituto Leonidas e Maria Deane - Fiocruz Amazonia	André Corado; Debora Duarte; Felipe Naveca; Fernanda Nascimento; George Silva; Karina Pessoa; Luciana Gonçalves; Maria Júlia Brandão; Matilde Mejía; Michele Jesus; Valdinete Nascimento; Victor Souza; Ágatha Costa
EPI_ISL_2008965	Laboratorio de Pesquisa em Virologia, FAMERP, SJRP	Laboratorio de Pesquisa em Virologia, FAMERP, SJRP	Cecília Artico Banho; Cíntia Bittar; Fábio Sossai Possebon; Guilherme Campos; Helena Lage Ferreira; Jorge A. Petroli Marchesi; João Pessoa Araújo Jr.; Leila Sabrina Ullmann; Lívia Sacchetto; Maisa C. Pereira Parra; Marília Moraes; Maurício L. Nogueira.; Paula Rahal; Paulo Inacio da Costa
EPI_ISL_2614091	Laboratory of Molecular Virology, Federal University of Rio de Janeito, UFRJ	Laboratory of Respiratory Viruses and Measles, Oswaldo Cruz Institute, FIOCRUZ	Alice Sampaio Rocha; Amilcar Tanuri; Ana Carolina Mendonca; Anna Carolina Paixao; Elisa Cavalcante Pereira; Fernando Motta; Luciana Appolinario; Marilda Siqueira on behalf of the Fiocruz COVID-19 Genomic Surveillance Network; Paola Resende; Renata Serrano Lopes; Taina Venas
EPI_ISL_2443582, EPI_ISL_2443587, EPI_ISL_2614319, EPI_ISL_2614320, EPI_ISL_2614321, EPI_ISL_2614322, EPI_ISL_2614323, EPI_ISL_2614324, EPI_ISL_2614325, EPI_ISL_2614326, EPI_ISL_2982741, EPI_ISL_3045541, EPI_ISL_3045542, EPI_ISL_3045543			
see above	Laboratory of Respiratory Viruses and Measles, Oswaldo Cruz Institute, FIOCRUZ	Laboratory of Respiratory Viruses and Measles, Oswaldo Cruz Institute, FIOCRUZ	Agatha Cristinne Prudencio; Alice Sampaio Rocha; Ana Carolina Mendonca; Anna Carolina Paixao; Elisa Cavalcante Pereira; Fernando Motta; Ighor Leonardo Arantes Gomes; Luciana Appolinario; Marilda Siqueira on behalf of the Fiocruz COVID-19 Genomic Surveillance Network; Paola Resende; Renata Serrano Lopes; Taina Venas
EPI_ISL_2777618, EPI_ISL_2777689	Laboratório Central de Saúde Pública do Amazonas - LACEN-AM	Laboratorio de Ecologia de Doencas Transmissiveis na Amazonia, Instituto Leonidas e Maria Deane - Fiocruz Amazonia	André Corado; Debora Duarte; Felipe Naveca; Fernanda Nascimento; George Silva; Karina Pessoa; Luciana Gonçalves; Maria Júlia Brandão; Matilde Mejía; Michele Jesus; Valdinete Nascimento; Victor Souza; Ágatha Costa
EPI_ISL_1495036	Laboratório de Biologia Integrativa	Laboratório de Biologia Integrativa	Alessandro Clayton de Souza Ferreira; Aline Brito de Lima; Carolina Moreira Voloch; Daniel Costa Queiroz; Danielle Alves Gomes Zauli; Diego Menezes Bonfim; Filipe Romero Rebello Moreira; Frederico Scott Varella Malta; Joice do Prado Silva; Lucyene Miguita Luiz; Nuno Rodrigues Faria; Paula Luize Camargos Fonseca; Rafael Marques de Souza; Renan Pedra de Souza; Renato Santana Aguiar; Rennan Garcias Moreira; Victor Cavalcanti Pardini; Victor Emmanuel Viana Geddes
EPI_ISL_2731572, EPI_ISL_2731623, EPI_ISL_2731629, EPI_ISL_2731638, EPI_ISL_2731639, EPI_ISL_2835133, EPI_ISL_2835139, EPI_ISL_2835143, EPI_ISL_2835205			
see above	Laboratório de Biotecnologia Aplicada (LBA) - Laboratório de Biologia Molecular - Hospital das Clínicas, Faculdade de Medicina de Botucatu, Departamento de Bioprocessos e Biotecnologia - Faculdade de Ciências Agronômicas, UNESP – Botucatu/SP	Laboratory of Respiratory Viruses and Measles, Oswaldo Cruz Institute, FIOCRUZ	Alice Sampaio Rocha; Ana Carolina Mendonca; Anna Carolina Paixao; Elisa Cavalcante Pereira; Felipe Allan da Silva Costa; Fernando Motta; Jayme Augusto de Souza Neto; Leonardo Nazario de Moraes; Luciana Appolinario; Marilda Siqueira on behalf of the Fiocruz COVID-19 Genomic Surveillance Network; Paola Resende; Patricia Akemi Assato; Rejane Maria Tommasini; Renata Serrano Lopes; Taina Venas
EPI_ISL_1464636, EPI_ISL_1464637, EPI_ISL_1464638	Laboratório de Virologia - UNIFESP	Laboratory of Respiratory Viruses and Measles, Oswaldo Cruz Institute, FIOCRUZ	Alice Sampaio Rocha; Ana Carolina Mendonca; Anna Carolina Paixao; Fernando Motta; Luciana Appolinario; Marilda Siqueira on behalf of the Fiocruz COVID-19 Genomic Surveillance Network; Nancy Belei; Paola Resende; Renata Serrano Lopes
EPI_ISL_2629753, EPI_ISL_2629754, EPI_ISL_2629755	Laboratório de Virologia Molecular - Universidade Federal do Rio de Janeiro	Laboratório de Virologia Molecular - Universidade Federal do Rio de Janeiro	; Alice Laschuk Herlinger; Amilcar Tanuri; André Felipe Andrade dos Santos; Carolina Moreira Voloch; Cássia Cristina Alves Gonçalves; Diana Mariani; Débora Souza Faffe; Filipe Romero Rebello Moreira; Francine Bittencourt Schiffler; Isabela de Carvalho Leitão; Marcelo Calado de Paula Tôrres; Matheus Augusto Calvano Cosentino; Mirela D'arc; Orlando da Costa Ferreira Junior; Rafael Mello Galliez; Raíssa Mirella dos Santos Cunha da Costa; Renato Santana de Aguiar; Terezinha Marta Pereira Pinto Castineiras; Thamiris dos Santos Miranda; Átila Duque Rossi
EPI_ISL_4037188	Laboratório de Virologia Molecular da Instituto Carlos Chagas da Fundação Oswaldo Cruz	Laboratório de Virologia Molecular da Instituto Carlos Chagas da Fundação Oswaldo Cruz	Antonio Ernesto Meister Luz Marques; Camila Zanluca; Claudia Nunes Duarte Santos.; Guilherme Soares; Hegger Fritsch; Luiz Carlos Junior Alcantara; Marta Giovanetti; Natalia Guimarães; Talita Adelino; Vagner Fonseca
EPI_ISL_2443689	Labortorio Central de Saude Publica do Estado de Santa Catarina (LACEN/SC)	Laboratory of Respiratory Viruses and Measles, Oswaldo Cruz Institute, FIOCRUZ	Alice Sampaio Rocha; Ana Carolina Mendonca; Anna Carolina Paixao; Darcita Buerger Rovaris; Elisa Cavalcante Pereira; Fernando Motta; Luciana Appolinario; Marilda Siqueira on behalf of the Fiocruz COVID-19 Genomic Surveillance Network; Paola Resende; Renata Serrano Lopes; Sandra Bianchini Fernandes; Taina Venas
EPI_ISL_2645508, EPI_ISL_2645509, EPI_ISL_2645511, EPI_ISL_2863595, EPI_ISL_2863598, EPI_ISL_2983228, EPI_ISL_2983229, EPI_ISL_2983416, EPI_ISL_2983417, EPI_ISL_2983421			
see above	Labortorio Central de Saude Publica do Estado do Tocantins (LACEN/TO)	Laboratory of Respiratory Viruses and Measles, Oswaldo Cruz Institute, FIOCRUZ	Alice Sampaio Rocha; Ana Carolina Mendonca; Anna Carolina Paixao; Elisa Cavalcante Pereira; Fernando Motta; Jucimaria Dantas Galvao; Luciana Appolinario; Marilda Siqueira on behalf of the Fiocruz COVID-19 Genomic Surveillance Network; Paola Resende; Renata Serrano Lopes; Taina Venas
EPI_ISL_2557362, EPI_ISL_2557374, EPI_ISL_2603451, EPI_ISL_2603453, EPI_ISL_2603462, EPI_ISL_2603463, EPI_ISL_2863859, EPI_ISL_2863864			
see above	Lboratorio Central de Saude Publica do Estado do Parana (LACEN/PR)	Laboratory of Respiratory Viruses and Measles, Oswaldo Cruz Institute, FIOCRUZ	Agatha Cristinne Prudencio Soares; Alice Sampaio Rocha; Ana Carolina Mendonca; Anna Carolina Paixao; Elisa Cavalcante Pereira; Fernando Motta; Ighor Leonardo Arantes Gomes; Irina Riediger; Luciana Appolinario; Marilda Siqueira on behalf of the Fiocruz COVID-19 Genomic Surveillance Network; Paola Resende; Renata Serrano Lopes; Taina Venas
EPI_ISL_2345261, EPI_ISL_2345276	NUCLEO DE SAUDE VILA FALCAO DE BAURU	Instituto Butantan / ESALQ-Piracicaba	Antonio Jorge Martins; Claudia Renata dos Santos Barros; David Schlesinger; Debora Botequio Moretti; Dimas Tadeu Covas; Elaine Cristina Marqueze; Elaine Vieira Santos; Evandra Strazza Rodrigues; Heidge Fukumasu; Jayme Augusto de Souza-Neto; José Salvatore Leister Patané; Luiz Alcantara; Luiz Lehmann Coutinho; Maria Carolina Elias; Maurício Lacerda Nogueira; Rafael dos Santos Bezerra; Raul Machado Neto; Rejane Maria Tommasini Grotto; Ricardo Haddad; Sandra Coccuzzo Sampaio Vessoni; Simone Kashima; Svetoslav Nanev Slavov; Vincent Louis Viala
EPI_ISL_2493828	PA DE IBITIUVA DR OTAVIO BENETTI PITANGUEIRAS	Instituto Butantan	Antonio Jorge Martins; Claudia Renata dos Santos Barros; David Schlesinger; Debora Botequio Moretti; Dimas Tadeu Covas; Elaine Cristina Marqueze; Elaine Vieira Santos; Evandra Strazza Rodrigues; Heidge Fukumasu; Jayme Augusto de Souza-Neto; José Salvatore Leister Patané; Luiz Alcantara; Luiz Lehmann Coutinho; Maria Carolina Elias; Maurício Lacerda Nogueira; Rafael dos Santos Bezerra; Raul Machado Neto; Rejane Maria Tommasini Grotto; Ricardo Haddad; Sandra Coccuzzo Sampaio Vessoni; Simone Kashima; Svetoslav Nanev Slavov; Vincent Louis Viala

EPI_ISL_2378748, EPI_ISL_2378749	PAS JOAO ANTONIO DO NASCIMENTO	Instituto Butantan	Antonio Jorge Martins; Claudia Renata dos Santos Barros; David Schlesinger; Debora Botequio Moretti; Dimas Tadeu Covas; Elaine Cristina Marqueze; Elaine Vieira Santos; Evandra Strazza Rodrigues; Heidge Fukumasu; Jayme Augusto de Souza-Neto; José Salvatore Leister Patané; Luiz Alcantara; Luiz Lehmann Coutinho; Maria Carolina Elias; Maurício Lacerda Nogueira; Rafael dos Santos Bezerra; Raul Machado Neto; Rejane Maria Tommasini Grotto; Ricardo Haddad; Sandra Coccuzzo Sampaio Vessoni; Simone Kashima; Svetoslav Nanev Slavov; Vincent Louis Viala
EPI_ISL_2378747	PROGRAMA SAUDE DA FAMILIA I IEPE	Instituto Butantan	Antonio Jorge Martins; Claudia Renata dos Santos Barros; David Schlesinger; Debora Botequio Moretti; Dimas Tadeu Covas; Elaine Cristina Marqueze; Elaine Vieira Santos; Evandra Strazza Rodrigues; Heidge Fukumasu; Jayme Augusto de Souza-Neto; José Salvatore Leister Patané; Luiz Alcantara; Luiz Lehmann Coutinho; Maria Carolina Elias; Maurício Lacerda Nogueira; Rafael dos Santos Bezerra; Raul Machado Neto; Rejane Maria Tommasini Grotto; Ricardo Haddad; Sandra Coccuzzo Sampaio Vessoni; Simone Kashima; Svetoslav Nanev Slavov; Vincent Louis Viala
EPI_ISL_1966135	PRONTO ATENDIMENTO VILA PADRE ANCHIETA	Instituto Butantan / Mendelics	Antonio Jorge Martins; Bianca Cechetto Carlos. Mendelics: Bibiana Santos; Claudia Renata dos Santos Barros; Cíntia Bittar; David Schlesinger. Hemocentro Ribeirão Preto: Simone Kashima; Debora Botequio Moretti; Elaine Cristina Marqueze; Elaine Vieira dos Santos; Elisangela Chicaroni Mattos; Erika Freitas; Evandra Strazza Rodrigues; Felipe Allan da Silva da Costa; Flavia Aburjaile; Fábio Sossai Possebon; Guilherme Campos; Guilherme Targino Valente; Heidge Fukumasu. USP-Botucatu: Rejane Maria Tommasini Grotto; Helena Lage Ferreira; Instituto Butantan: Dimas Tadeu Covas; Jardelina de Souza Todao Bernardino; Jayme A. Souza-Neto; Jessika Cristina Chagas Lesbon; Jorge A. Petroli Marchesi; José Salvatore Leister Patané; João Paulo Kitajima; João Pessoa Araújo Jr.; Leila Sabrina Ullmann; Loyze Paola Oliveira de Lima; Luiz Aurelio de Campos Crispin. Centro de Genômica Funcional da ESALQ: Luiz Lehmann Coutinho; Luiz Carlos Junior de Alcantara; Lívia Sacchetto; Maisa C. Pereira Parra; Maria Carolina Elias; Marta Giovanetti; Marília Moraes; Maurício Lacerda Nogueira. Prefeitura de Sao Paulo: Melissa Palmieri.; Patricia Akemi Assato; Paula Rahal; Paulo Inacio da Costa; Rafael dos Santos Bezerra; Raquel de Lello Rocha Campos Cassano. NGS Soluções Genômicas: Pilar Drummond Sampaio Corrêa Mariani. FZEA-USP Pirassununga: Mirele Daiana Poleti; Raul Machado Neto; Ricardo Augusto Brassaloti; Ricardo Haddad; Rodrigo Tocantins Calado. FAMERP-SJRP: Cecília Artico Banho; Sandra Coccuzzo Sampaio; Svetoslav Nanev Slavov; Vagner Fonseca; Vincent Louis Viala
EPI_ISL_2375891	Programa de Oncovirologia, Instituto Nacional de Câncer	Programa de Oncovirologia, Instituto Nacional de Câncer	Ana Cristina P. M. Pereira; Brunna M. Alves; Claudia Cicala; James Arthos; João P.B. Viola; Juliana D. Siqueira; Livia R. Goes; Marcelo A. Soares; Marianne M. Garrido
EPI_ISL_3102417	SAO CARLOS DIAGNOSTICO POR IMAGEM	Analytical Competence Molecular Epidemiology Lab/ACME, Oswaldo Cruz Foundation, Ceara (FIOCRUZ CE)	Cleber Furtado Aksenen; Fabio Miyajima; Fernando Braga Stehling; Francisco Eder de Moura Lopes; Jamille Maria Mendes Bezerra; Joaquim César do Nascimento Sousa Junior; Pedro Miguel Carneiro Jeronimo; Suzana Porto Almeida e Lucas Delerino; Thais Ferreira de Oliveira; Thais de Oliveira Costa; Ticiane Cavalcante de Souza; Veridiana Pessoa Miyajima
EPI_ISL_2444158	SAO JOSE DO RIO PRETO	Instituto Butantan / FAMERP	Antonio Jorge Martins; Claudia Renata dos Santos Barros; David Schlesinger; Debora Botequio Moretti; Dimas Tadeu Covas; Elaine Cristina Marqueze; Elaine Vieira Santos; Evandra Strazza Rodrigues; Heidge Fukumasu; Jayme Augusto de Souza-Neto; José Salvatore Leister Patané; Luiz Alcantara; Luiz Lehmann Coutinho; Maria Carolina Elias; Maurício Lacerda Nogueira; Rafael dos Santos Bezerra; Raul Machado Neto; Rejane Maria Tommasini Grotto; Ricardo Haddad; Sandra Coccuzzo Sampaio Vessoni; Simone Kashima; Svetoslav Nanev Slavov; Vincent Louis Viala
EPI_ISL_1967267, EPI_ISL_1967271, EPI_ISL_1967274	SECAO CENTRO DE DIAGNOSTICO SECEDI	Instituto Butantan / Mendelics	Antonio Jorge Martins; Bianca Cechetto Carlos. Mendelics: Bibiana Santos; Claudia Renata dos Santos Barros; Cíntia Bittar; David Schlesinger. Hemocentro Ribeirão Preto: Simone Kashima; Debora Botequio Moretti; Elaine Cristina Marqueze; Elaine Vieira dos Santos; Elisangela Chicaroni Mattos; Erika Freitas; Evandra Strazza Rodrigues; Felipe Allan da Silva da Costa; Flavia Aburjaile; Fábio Sossai Possebon; Guilherme Campos; Guilherme Targino Valente; Heidge Fukumasu. USP-Botucatu: Rejane Maria Tommasini Grotto; Helena Lage Ferreira; Instituto Butantan: Dimas Tadeu Covas; Jardelina de Souza Todao Bernardino; Jayme A. Souza-Neto; Jessika Cristina Chagas Lesbon; Jorge A. Petroli Marchesi; José Salvatore Leister Patané; João Paulo Kitajima; João Pessoa Araújo Jr.; Leila Sabrina Ullmann; Loyze Paola Oliveira de Lima; Luiz Aurelio de Campos Crispin. Centro de Genômica Funcional da ESALQ: Luiz Lehmann Coutinho; Luiz Carlos Junior de Alcantara; Lívia Sacchetto; Maisa C. Pereira Parra; Maria Carolina Elias; Marta Giovanetti; Marília Moraes; Maurício Lacerda Nogueira. Prefeitura de Sao Paulo: Melissa Palmieri.; Patricia Akemi Assato; Paula Rahal; Paulo Inacio da Costa; Rafael dos Santos Bezerra; Raquel de Lello Rocha Campos Cassano. NGS Soluções Genômicas: Pilar Drummond Sampaio Corrêa Mariani. FZEA-USP Pirassununga: Mirele Daiana Poleti; Raul Machado Neto; Ricardo Augusto Brassaloti; Ricardo Haddad; Rodrigo Tocantins Calado. FAMERP-SJRP: Cecília Artico Banho; Sandra Coccuzzo Sampaio; Svetoslav Nanev Slavov; Vagner Fonseca; Vincent Louis Viala
EPI_ISL_2345661	SECRETARIA DE SAUDE PUBLICA DE PRAIA GRANDE	Instituto Butantan / Mendelics	Antonio Jorge Martins; Claudia Renata dos Santos Barros; David Schlesinger; Debora Botequio Moretti; Dimas Tadeu Covas; Elaine Cristina Marqueze; Elaine Vieira Santos; Evandra Strazza Rodrigues; Heidge Fukumasu; Jayme Augusto de Souza-Neto; José Salvatore Leister Patané; Luiz Alcantara; Luiz Lehmann Coutinho; Maria Carolina Elias; Maurício Lacerda Nogueira; Rafael dos Santos Bezerra; Raul Machado Neto; Rejane Maria Tommasini Grotto; Ricardo Haddad; Sandra Coccuzzo Sampaio Vessoni; Simone Kashima; Svetoslav Nanev Slavov; Vincent Louis Viala
EPI_ISL_2494122	UBS DR ALFREDO DANTAS DE SOUZA UMUARAMA	Instituto Butantan	Antonio Jorge Martins; Claudia Renata dos Santos Barros; David Schlesinger; Debora Botequio Moretti; Dimas Tadeu Covas; Elaine Cristina Marqueze; Elaine Vieira Santos; Evandra Strazza Rodrigues; Heidge Fukumasu; Jayme Augusto de Souza-Neto; José Salvatore Leister Patané; Luiz Alcantara; Luiz Lehmann Coutinho; Maria Carolina Elias; Maurício Lacerda Nogueira; Rafael dos Santos Bezerra; Raul Machado Neto; Rejane Maria Tommasini Grotto; Ricardo Haddad; Sandra Coccuzzo Sampaio Vessoni; Simone Kashima; Svetoslav Nanev Slavov; Vincent Louis Viala
EPI_ISL_2378746	UBS II DE PIRAPOZINHO C SERVICO DE EACS E ESF	Instituto Butantan	Antonio Jorge Martins; Claudia Renata dos Santos Barros; David Schlesinger; Debora Botequio Moretti; Dimas Tadeu Covas; Elaine Cristina Marqueze; Elaine Vieira Santos; Evandra Strazza Rodrigues; Heidge Fukumasu; Jayme Augusto de Souza-Neto; José Salvatore Leister Patané; Luiz Alcantara; Luiz Lehmann Coutinho; Maria Carolina Elias; Maurício Lacerda Nogueira; Rafael dos Santos Bezerra; Raul Machado Neto; Rejane Maria Tommasini Grotto; Ricardo Haddad; Sandra Coccuzzo Sampaio Vessoni; Simone Kashima; Svetoslav Nanev Slavov; Vincent Louis Viala
EPI_ISL_2378750	UNIDADE DE SAUDE DA FAMILIA DE FLORIDA PAULISTA II	Instituto Butantan	Antonio Jorge Martins; Claudia Renata dos Santos Barros; David Schlesinger; Debora Botequio Moretti; Dimas Tadeu Covas; Elaine Cristina Marqueze; Elaine Vieira Santos; Evandra Strazza Rodrigues; Heidge Fukumasu; Jayme Augusto de Souza-Neto; José Salvatore Leister Patané; Luiz Alcantara; Luiz Lehmann Coutinho; Maria Carolina Elias; Maurício Lacerda Nogueira; Rafael dos Santos Bezerra; Raul Machado Neto; Rejane Maria Tommasini Grotto; Ricardo Haddad; Sandra Coccuzzo Sampaio Vessoni; Simone Kashima; Svetoslav Nanev Slavov; Vincent Louis Viala
EPI_ISL_2378742, EPI_ISL_2445486, EPI_ISL_2445487, EPI_ISL_2445488, EPI_ISL_2445489, EPI_ISL_2445490, EPI_ISL_2445491, EPI_ISL_2473784			
see above	UNIDADE DE SAUDE DR PHEBO DE OLIVEIRA ROGE FERREIRA	Instituto Butantan	Antonio Jorge Martins; Claudia Renata dos Santos Barros; David Schlesinger; Debora Botequio Moretti; Dimas Tadeu Covas; Elaine Cristina Marqueze; Elaine Vieira Santos; Evandra Strazza Rodrigues; Heidge Fukumasu; Jayme Augusto de Souza-Neto; José Salvatore Leister Patané; Luiz Alcantara; Luiz Lehmann Coutinho; Maria Carolina Elias; Maurício Lacerda Nogueira; Rafael dos Santos Bezerra; Raul Machado Neto; Rejane Maria Tommasini Grotto; Ricardo Haddad; Sandra Coccuzzo Sampaio Vessoni; Simone Kashima; Svetoslav Nanev Slavov; Vincent Louis Viala
EPI_ISL_2344818	UNIDADE MISTA DE IGUAPE	Instituto Butantan / ESALQ-Piracicaba	Antonio Jorge Martins; Claudia Renata dos Santos Barros; David Schlesinger; Debora Botequio Moretti; Dimas Tadeu Covas; Elaine Cristina Marqueze; Elaine Vieira Santos; Evandra Strazza Rodrigues; Heidge Fukumasu; Jayme Augusto de Souza-Neto; José Salvatore Leister Patané; Luiz Alcantara; Luiz Lehmann Coutinho; Maria Carolina Elias; Maurício Lacerda Nogueira; Rafael dos Santos Bezerra; Raul Machado Neto; Rejane Maria Tommasini Grotto; Ricardo Haddad; Sandra Coccuzzo Sampaio Vessoni; Simone Kashima; Svetoslav Nanev Slavov; Vincent Louis Viala
EPI_ISL_1966682	UNIDADE MISTA DE IGUAPE	Instituto Butantan / Mendelics	Antonio Jorge Martins; Bianca Cechetto Carlos. Mendelics: Bibiana Santos; Claudia Renata dos Santos Barros; Cíntia Bittar; David Schlesinger. Hemocentro Ribeirão Preto: Simone Kashima; Debora Botequio Moretti; Elaine Cristina Marqueze; Elaine Vieira dos Santos; Elisangela Chicaroni Mattos; Erika Freitas; Evandra Strazza Rodrigues; Felipe Allan da Silva da Costa; Flavia Aburjaile; Fábio Sossai Possebon; Guilherme Campos; Guilherme Targino Valente; Heidge Fukumasu. USP-Botucatu: Rejane Maria Tommasini Grotto; Helena Lage Ferreira; Instituto Butantan: Dimas Tadeu Covas; Jardelina de Souza Todao Bernardino; Jayme A. Souza-Neto; Jessika Cristina Chagas Lesbon; Jorge A. Petroli Marchesi; José Salvatore Leister Patané; João Paulo Kitajima; João Pessoa Araújo Jr.; Leila Sabrina Ullmann; Loyze Paola Oliveira de Lima; Luiz Aurelio de Campos Crispin. Centro de Genômica Funcional da ESALQ: Luiz Lehmann Coutinho; Luiz Carlos Junior de Alcantara; Lívia Sacchetto; Maisa C. Pereira Parra; Maria Carolina Elias; Marta Giovanetti; Marília Moraes; Maurício Lacerda Nogueira. Prefeitura de Sao Paulo: Melissa Palmieri.; Patricia Akemi Assato; Paula Rahal; Paulo Inacio da Costa; Rafael dos Santos Bezerra; Raquel de Lello Rocha Campos Cassano. NGS Soluções Genômicas: Pilar Drummond Sampaio Corrêa Mariani. FZEA-USP Pirassununga: Mirele Daiana Poleti; Raul Machado Neto; Ricardo Augusto Brassaloti; Ricardo Haddad; Rodrigo Tocantins Calado. FAMERP-SJRP: Cecília Artico Banho; Sandra Coccuzzo Sampaio; Svetoslav Nanev Slavov; Vagner Fonseca; Vincent Louis Viala
EPI_ISL_3102488	UNIDADE PRONTO ATENDIMENTO AUTRAN NUNES	Analytical Competence Molecular Epidemiology Lab/ACME, Oswaldo Cruz Foundation, Ceara (FIOCRUZ CE)	Cleber Furtado Aksenen; Fabio Miyajima; Fernando Braga Stehling; Francisco Eder de Moura Lopes; Jamille Maria Mendes Bezerra; Joaquim César do Nascimento Sousa Junior; Pedro Miguel Carneiro Jeronimo; Suzana Porto Almeida e Lucas Delerino; Thais Ferreira de Oliveira; Thais de Oliveira Costa; Ticiane Cavalcante de Souza; Veridiana Pessoa Miyajima
EPI_ISL_2378752	UNIDADE REFERENCIAL SUDOESTE	Instituto Butantan	Antonio Jorge Martins; Claudia Renata dos Santos Barros; David Schlesinger; Debora Botequio Moretti; Dimas Tadeu Covas; Elaine Cristina Marqueze; Elaine Vieira Santos; Evandra Strazza Rodrigues; Heidge Fukumasu; Jayme Augusto de Souza-Neto; José Salvatore Leister Patané; Luiz Alcantara; Luiz Lehmann Coutinho; Maria Carolina Elias; Maurício Lacerda Nogueira; Rafael dos Santos Bezerra; Raul Machado Neto; Rejane Maria Tommasini Grotto; Ricardo Haddad; Sandra Coccuzzo Sampaio Vessoni; Simone Kashima; Svetoslav Nanev Slavov; Vincent Louis Viala
EPI_ISL_1966342	UPA 24 HORAS CENTRO	Instituto Butantan / Mendelics	Antonio Jorge Martins; Bianca Cechetto Carlos. Mendelics: Bibiana Santos; Claudia Renata dos Santos Barros; Cíntia Bittar; David Schlesinger. Hemocentro Ribeirão Preto: Simone Kashima; Debora Botequio Moretti; Elaine Cristina Marqueze; Elaine Vieira dos Santos; Elisangela Chicaroni Mattos; Erika Freitas; Evandra Strazza Rodrigues; Felipe Allan da Silva da Costa; Flavia Aburjaile; Fábio Sossai Possebon; Guilherme Campos; Guilherme Targino Valente; Heidge Fukumasu. USP-Botucatu: Rejane Maria Tommasini Grotto; Helena Lage Ferreira; Instituto Butantan: Dimas Tadeu Covas; Jardelina de Souza Todao Bernardino; Jayme A. Souza-Neto; Jessika Cristina Chagas Lesbon; Jorge A. Petroli Marchesi; José Salvatore Leister Patané; João Paulo Kitajima; João Pessoa Araújo Jr.; Leila Sabrina Ullmann; Loyze Paola Oliveira de Lima; Luiz Aurelio de Campos Crispin. Centro de Genômica Funcional da ESALQ: Luiz Lehmann Coutinho; Luiz Carlos Junior de Alcantara; Lívia Sacchetto; Maisa C. Pereira Parra; Maria Carolina Elias; Marta Giovanetti; Marília Moraes; Maurício Lacerda Nogueira. Prefeitura de Sao Paulo: Melissa Palmieri.; Patricia Akemi Assato; Paula Rahal; Paulo Inacio da Costa; Rafael dos Santos Bezerra; Raquel de Lello Rocha Campos Cassano. NGS Soluções Genômicas: Pilar Drummond Sampaio Corrêa Mariani. FZEA-USP Pirassununga: Mirele Daiana Poleti; Raul Machado Neto; Ricardo Augusto Brassaloti; Ricardo Haddad; Rodrigo Tocantins Calado. FAMERP-SJRP: Cecília Artico Banho; Sandra Coccuzzo Sampaio; Svetoslav Nanev Slavov; Vagner Fonseca; Vincent Louis Viala
EPI_ISL_2344765	UPA 24 HORAS DR ALOISIO MUNIZ DE ANDRADE	Instituto Butantan / ESALQ-Piracicaba	Antonio Jorge Martins; Claudia Renata dos Santos Barros; David Schlesinger; Debora Botequio Moretti; Dimas Tadeu Covas; Elaine Cristina Marqueze; Elaine Vieira Santos; Evandra Strazza Rodrigues; Heidge Fukumasu; Jayme Augusto de Souza-Neto; José Salvatore Leister Patané; Luiz Alcantara; Luiz Lehmann Coutinho; Maria Carolina Elias; Maurício Lacerda Nogueira; Rafael dos Santos Bezerra; Raul Machado Neto; Rejane Maria Tommasini Grotto; Ricardo Haddad; Sandra Coccuzzo Sampaio Vessoni; Simone Kashima; Svetoslav Nanev Slavov; Vincent Louis Viala
EPI_ISL_2378737, EPI_ISL_2378740, EPI_ISL_2378741, EPI_ISL_2462322, EPI_ISL_2494116, EPI_ISL_2494124, EPI_ISL_2494125			
see above	UPA DE BEBEDOURO	Instituto Butantan	Antonio Jorge Martins; Claudia Renata dos Santos Barros; David Schlesinger; Debora Botequio Moretti; Dimas Tadeu Covas; Elaine Cristina Marqueze; Elaine Vieira Santos; Evandra Strazza Rodrigues; Heidge Fukumasu; Jayme Augusto de Souza-Neto; José Salvatore Leister Patané; Luiz Alcantara; Luiz Lehmann Coutinho; Maria Carolina Elias; Maurício Lacerda Nogueira; Rafael dos Santos Bezerra; Raul Machado Neto; Rejane Maria Tommasini Grotto; Ricardo Haddad; Sandra Coccuzzo Sampaio Vessoni; Simone Kashima; Svetoslav Nanev Slavov; Vincent Louis Viala
EPI_ISL_1966600, EPI_ISL_1967220, EPI_ISL_1967223, EPI_ISL_2170972	UPA DE BEBEDOURO	Instituto Butantan / Mendelics	Antonio Jorge Martins; Bianca Cechetto Carlos. Mendelics: Bibiana Santos; Claudia Renata dos Santos Barros; Cíntia Bittar; David Schlesinger. Hemocentro Ribeirão Preto: Simone Kashima; Debora Botequio Moretti; Elaine Cristina Marqueze; Elaine Vieira dos Santos; Elisangela Chicaroni Mattos; Erika Freitas; Evandra Strazza Rodrigues; Felipe Allan da Silva da Costa; Flavia Aburjaile; Fábio Sossai Possebon; Guilherme Campos; Guilherme Targino Valente; Heidge Fukumasu. USP-Botucatu: Rejane Maria Tommasini Grotto; Helena Lage Ferreira; Instituto Butantan: Dimas Tadeu Covas; Jardelina de Souza Todao Bernardino; Jayme A. Souza-Neto; Jessika Cristina Chagas Lesbon; Jorge A. Petroli Marchesi; José Salvatore Leister Patané; João Paulo Kitajima; João Pessoa Araújo Jr.; Leila Sabrina Ullmann; Loyze Paola Oliveira de Lima; Luiz Aurelio de Campos Crispin. Centro de Genômica Funcional da ESALQ: Luiz Lehmann Coutinho; Luiz Carlos Junior de Alcantara; Lívia Sacchetto; Maisa C. Pereira Parra; Maria Carolina Elias; Marta Giovanetti; Marília Moraes; Maurício Lacerda Nogueira. Prefeitura de Sao Paulo: Melissa Palmieri.; Patricia Akemi Assato; Paula Rahal; Paulo Inacio da Costa; Rafael dos Santos Bezerra; Raquel de Lello Rocha Campos Cassano. NGS Soluções Genômicas: Pilar Drummond Sampaio Corrêa Mariani. FZEA-USP Pirassununga: Mirele Daiana Poleti; Raul Machado Neto; Ricardo Augusto Brassaloti; Ricardo Haddad; Rodrigo Tocantins Calado. FAMERP-SJRP: Cecília Artico Banho; Sandra Coccuzzo Sampaio; Svetoslav Nanev Slavov; Vagner Fonseca; Vincent Louis Viala
EPI_ISL_2378736	UPA II JUNDIAI	Instituto Butantan	Antonio Jorge Martins; Claudia Renata dos Santos Barros; David Schlesinger; Debora Botequio Moretti; Dimas Tadeu Covas; Elaine Cristina Marqueze; Elaine Vieira Santos; Evandra Strazza Rodrigues; Heidge Fukumasu; Jayme Augusto de Souza-Neto; José Salvatore Leister Patané; Luiz Alcantara; Luiz Lehmann Coutinho; Maria Carolina Elias; Maurício Lacerda Nogueira; Rafael dos Santos Bezerra; Raul Machado Neto; Rejane Maria Tommasini Grotto; Ricardo Haddad; Sandra Coccuzzo Sampaio Vessoni; Simone Kashima; Svetoslav Nanev Slavov; Vincent Louis Viala
EPI_ISL_2378738	USAFA FORTE	Instituto Butantan	Antonio Jorge Martins; Claudia Renata dos Santos Barros; David Schlesinger; Debora Botequio Moretti; Dimas Tadeu Covas; Elaine Cristina Marqueze; Elaine Vieira Santos; Evandra Strazza Rodrigues; Heidge Fukumasu; Jayme Augusto de Souza-Neto; José Salvatore Leister Patané; Luiz Alcantara; Luiz Lehmann Coutinho; Maria Carolina Elias; Maurício Lacerda Nogueira; Rafael dos Santos Bezerra; Raul Machado Neto; Rejane Maria Tommasini Grotto; Ricardo Haddad; Sandra Coccuzzo Sampaio Vessoni; Simone Kashima; Svetoslav Nanev Slavov; Vincent Louis Viala
EPI_ISL_2378751, EPI_ISL_2445527	USF PAULISTA FERNANDOPOLIS ANTONIO PIVATO	Instituto Butantan	Antonio Jorge Martins; Claudia Renata dos Santos Barros; David Schlesinger; Debora Botequio Moretti; Dimas Tadeu Covas; Elaine Cristina Marqueze; Elaine Vieira Santos; Evandra Strazza Rodrigues; Heidge Fukumasu; Jayme Augusto de Souza-Neto; José Salvatore Leister Patané; Luiz Alcantara; Luiz Lehmann Coutinho; Maria Carolina Elias; Maurício Lacerda Nogueira; Rafael dos Santos Bezerra; Raul Machado Neto; Rejane Maria Tommasini Grotto; Ricardo Haddad; Sandra Coccuzzo Sampaio Vessoni; Simone Kashima; Svetoslav Nanev Slavov; Vincent Louis Viala
EPI_ISL_1795391, EPI_ISL_2345528	USF ROSA CRUZ	Instituto Butantan / ESALQ-Piracicaba	Antonio Jorge Martins; Bianca Cechetto Carlos. Mendelics: Bibiana Santos; Claudia Renata dos Santos Barros; David Schlesinger; David Schlesinger. Hemocentro Ribeirão Preto: Simone Kashima; Debora Botequio Moretti; Debora Botequio Moretti. Centro de Genômica Funcional da ESALQ: Luiz Lehmann Coutinho; Dimas Tadeu Covas; Elaine Cristina Marqueze; Elaine Vieira Santos; Elaine Vieira dos Santos; Elisangela Chicaroni Mattos; Erika Freitas; Evandra Strazza Rodrigues; Felipe Allan da Silva da Costa; Flavia Aburjaile; Guilherme Targino Valente; Heidge Fukumasu; Heidge Fukumasu. USP-Botucatu: Rejane Maria Tommasini Grotto; Instituto Butantan: Alexander Roberto Precioso; Jayme A. Souza-Neto; Jayme Augusto de Souza-Neto; Jessika Cristina Chagas Lesbon; José Salvatore Leister Patané; João Paulo Kitajima; Luiz Alcantara; Luiz Carlos Junior de Alcantara; Luiz Lehmann Coutinho; Maria Carolina Elias; Marta Giovanetti; Maurício Lacerda Nogueira; Patricia Akemi Assato; Rafael dos Santos Bezerra; Raquel de Lello Rocha Campos Cassano. NGS Soluções Genômicas: Pilar Drummond Sampaio Corrêa Mariani. FZEA-USP Pirassununga: Mirele Daiana Poleti; Raul Machado Neto; Rejane Maria Tommasini Grotto; Ricardo Augusto Brassaloti; Ricardo Haddad; Rodrigo Tocantins Calado.; Sandra Coccuzzo Sampaio; Sandra Coccuzzo Sampaio Vessoni; Simone Kashima; Svetoslav Nanev Slavov; Vagner Fonseca; Vincent Louis Viala
EPI_ISL_2535122, EPI_ISL_2535177, EPI_ISL_2691654, EPI_ISL_2837278, EPI_ISL_2837286	Unidade de apoio ao diagnostico da COVID - UNADIG	Bioinformatics Laboratory / LNCC	Alessandra P Lamarca; Alexandra L Gerber; Amilcar Tanuri; Ana Paula de C Guimaraes; Ana Tereza R Vasconcelos; Andrea Cony Cavalcanti; Caio Luiz Pereira Ribeiro; Cassia Alves; Cintia Policarpo; Claudia Maria Braga de Mello; Cristiane Gomes da Silva; Diana Mariani; Douglas Terra Machado; Flavio Dias da Silva; Gleidson da Silva de Oliveira; Leandro Magalhaes de Souza; Liliane Cavalcante; Luiz G P de Almeida; Marcio Henrique de Oliveira Garcia; Mario Sergio Ribeiro; Ronaldo da Silva F Jr; Silvia Carvalho
EPI_ISL_2101734	Unidade de apoio ao diagnóstico da COVID – UNADIG	Bioinformatics Laboratory / LNCC	Alessandra P Lamarca; Alexandra L Gerber; Amilcar Tanuri; Ana Paula de C Guimaraes; Ana Tereza R Vasconcelos; Andrea Cony Cavalcanti; Caio Luiz Pereira Ribeiro; Cassia Alves; Cintia Policarpo; Claudia Maria Braga de Mello; Cristiane Gomes da Silva; Diana Mariani; Douglas Terra Machado; Flavio Dias da Silva; Gleidson da Silva de Oliveira; Leandro Magalhaes de Souza; Liliane Cavalcante; Luiz G P de Almeida; Marcio Henrique de Oliveira Garcia; Mario Sergio Ribeiro; Ronaldo da Silva F Jr; Silvia Carvalho
EPI_ISL_1795342, EPI_ISL_2345608	VIGILANCIA EM SAUDE	Instituto Butantan / ESALQ-Piracicaba	Antonio Jorge Martins; Bianca Cechetto Carlos. Mendelics: Bibiana Santos; Claudia Renata dos Santos Barros; David Schlesinger; David Schlesinger. Hemocentro Ribeirão Preto: Simone Kashima; Debora Botequio Moretti; Debora Botequio Moretti. Centro de Genômica Funcional da ESALQ: Luiz Lehmann Coutinho; Dimas Tadeu Covas; Elaine Cristina Marqueze; Elaine Vieira Santos; Elaine Vieira dos Santos; Elisangela Chicaroni Mattos; Erika Freitas; Evandra Strazza Rodrigues; Felipe Allan da Silva da Costa; Flavia Aburjaile; Guilherme Targino Valente; Heidge Fukumasu; Heidge Fukumasu. USP-Botucatu: Rejane Maria Tommasini Grotto; Instituto Butantan: Alexander Roberto Precioso; Jayme A. Souza-Neto; Jayme Augusto de Souza-Neto; Jessika Cristina Chagas Lesbon; José Salvatore Leister Patané; João Paulo Kitajima; Luiz Alcantara; Luiz Carlos Junior de Alcantara; Luiz Lehmann Coutinho; Maria Carolina Elias; Marta Giovanetti; Maurício Lacerda Nogueira; Patricia Akemi Assato; Rafael dos Santos Bezerra; Raquel de Lello Rocha Campos Cassano. NGS Soluções Genômicas: Pilar Drummond Sampaio Corrêa Mariani. FZEA-USP Pirassununga: Mirele Daiana Poleti; Raul Machado Neto; Rejane Maria Tommasini Grotto; Ricardo Augusto Brassaloti; Ricardo Haddad; Rodrigo Tocantins Calado.; Sandra Coccuzzo Sampaio; Sandra Coccuzzo Sampaio Vessoni; Simone Kashima; Svetoslav Nanev Slavov; Vagner Fonseca; Vincent Louis Viala

EPI_ISL_2383034	VIGILANCIA EPIDEMIOLOGICA E CONTROLE DE VETORES PIRASSUNUN	Instituto Butantan	Antonio Jorge Martins; Claudia Renata dos Santos Barros; David Schlesinger; Debora Botequio Moretti; Dimas Tadeu Covas; Elaine Cristina Marqueze; Elaine Vieira Santos; Evandra Strazza Rodrigues; Heidge Fukumasu; Jayme Augusto de Souza-Neto; José Salvatore Leister Patané; Luiz Alcantara; Luiz Lehmann Coutinho; Maria Carolina Elias; Maurício Lacerda Nogueira; Rafael dos Santos Bezerra; Raul Machado Neto; Rejane Maria Tommasini Grotto; Ricardo Haddad; Sandra Coccuzzo Sampaio Vessoni; Simone Kashima; Svetoslav Nanev Slavov; Vincent Louis Viala
